# Comparative Genomic and Transcriptomic Analysis of *Wangiella dermatitidis*, A Major Cause of Phaeohyphomycosis and a Model Black Yeast Human Pathogen

**DOI:** 10.1534/g3.113.009241

**Published:** 2014-02-04

**Authors:** Zehua Chen, Diego A. Martinez, Sharvari Gujja, Sean M. Sykes, Qiandong Zeng, Paul J. Szaniszlo, Zheng Wang, Christina A. Cuomo

**Affiliations:** * Broad Institute of Massachusetts Institute of Technology and Harvard, Cambridge, Massachusetts 02142; † Department of Molecular Biosciences, The University of Texas at Austin, Austin, Texas 78712; ‡ Center for Bio/Molecular Science and Engineering, Naval Research Laboratory, Washington, DC 20375

**Keywords:** human pathogenic fungi, comparative genomics, RNA-Seq, cell wall biosynthesis, horizontal gene transfer

## Abstract

Black or dark brown (phaeoid) fungi cause cutaneous, subcutaneous, and systemic infections in humans. Black fungi thrive in stressful conditions such as intense light, high radiation, and very low pH. *Wangiella* (*Exophiala*) *dermatitidis* is arguably the most studied phaeoid fungal pathogen of humans. Here, we report our comparative analysis of the genome of *W. dermatitidis* and the transcriptional response to low pH stress. This revealed that *W. dermatitidis* has lost the ability to synthesize alpha-glucan, a cell wall compound many pathogenic fungi use to evade the host immune system. In contrast, *W. dermatitidis* contains a similar profile of chitin synthase genes as related fungi and strongly induces genes involved in cell wall synthesis in response to pH stress. The large portfolio of transporters may provide *W. dermatitidis* with an enhanced ability to remove harmful products as well as to survive on diverse nutrient sources. The genome encodes three independent pathways for producing melanin, an ability linked to pathogenesis; these are active during pH stress, potentially to produce a barrier to accumulated oxidative damage that might occur under stress conditions. In addition, a full set of fungal light-sensing genes is present, including as part of a carotenoid biosynthesis gene cluster. Finally, we identify a two-gene cluster involved in nucleotide sugar metabolism conserved with a subset of fungi and characterize a horizontal transfer event of this cluster between fungi and algal viruses. This work reveals how *W. dermatitidis* has adapted to stress and survives in diverse environments, including during human infections.

A diverse array of primary and secondary diseases in humans is caused by a plethora of fungi with dark brown to black (phaeoid) vegetative growth at all stages of development (Matsumoto *et al.* 1994). Traditionally, these mycoses mostly have been associated with chronic dermatotropic forms of cutaneous and subcutaneous phaeohyphomycosis, chromoblastomycosis, and black-grain eumycotic mycetoma (Matsumoto *et al.* 2011). Recently, however, an increasing frequency of systemic forms, particularly among patients with phaeohyphomycosis, has been reported in immunocompromised individuals and immunocompetent individuals (Zeng *et al.* 2007). In extreme cases, the systemic forms may manifest as neurotropic and cerebral disease with no clear evidence of initial site of infection (Chang *et al.* 2000; Sutton *et al.* 1998). Although the distributions of the known pathogenic phaeoid fungi were once believed limited to tropical and subtropical regions, today these species and their potentially pathogenic relatives are being detected worldwide in widely diverse ecological settings. No matter what the origin, whether infected human tissue, partners in lichens, saprophytes in leaf liter or on other woody materials, surfaces in the interior of the Chernobyl nuclear reactor, the extreme environments at polar regions, or bare rock at the top of mountains, the unifying characteristic of all phaeoid fungi is the deposition of 1,8-dihydroxynaphthalene melanin into its cell walls at all stages of development (Dadachova *et al.* 2007; Feng *et al.* 2001; Wheeler *et al.* 2008; Schnitzler *et al.* 1999).

Among phaeoid fungi in general and human pathogenic phaeoid fungi in particular, *Wangiella dermatitidis* (also referred to as *Exophiala dermatitidis*) is the best known species for a number of reasons. The two most important of these are that it is a paradigm for phaeohyphomycosis and it is the model of choice for the more than 100 other phaeoid fungi known to cause human disease (Matsumoto *et al.* 1994; Szaniszlo 2002, 2006). The latter is particularly important because although each phaeoid agent may cause relatively few infections, worldwide each year the phaeoid fungi collectively give rise to large numbers of cases that are notoriously troublesome, often require surgery, and frequently are associated with failed antifungal therapy and death (Matsumoto *et al.* 1994, 2011; Zeng *et al.* 2007; Chang *et al.* 2000; Chua *et al.* 2001; Filizzola *et al.* 2003). Thus, the clinical diversity, the large number of agents, and the impracticality of studying each of the many phaeoid agents of disease in great depth dictate that a model approach is necessary to gain insights into the varied biology of the ever-expanding repertoire of phaeoid fungi associated with human disease.

A recent example of the need for an overall better understanding of the phaeoid fungi is related to the discovery by the medical community of the virtual absence of important biological knowledge of the phaeoid mold *Exserohilum rostratum* (Centers for Disease Control and Prevention 2012). This usually benign and relatively unstudied fungus as of 5 November 2012 had caused more than 749 cases of phaeohyphomycotic meningitis and at least 61 deaths in 19 states in the United States (Andes and Casadevall 2013) when it was injected as a contaminant of pharmaceutically compounded steroid solutions administered for pain management. In contrast with the cutaneous and subcutaneous forms of phaeohyphomycosis, surgery is not an option for these patients; instead, they require long-term antifungal therapy that can be dangerous and often fails. Just 10 yr previously, *W. dermatitidis* itself was the cause of a similar but smaller outbreak of fungal meningitis attributable to the injection of contaminated steroids (Centers for Disease Control and Prevention 2002). Additional parallels can be extrapolated from studies of the *W. dermatitidis* model; 1,8-DHN melanin, a known virulence factor of *W. dermatitidis*, was also identified in *E. rostratum* and could similarly impart resistance to host killing and antifungal drug therapy (Paolo *et al.* 2006; Al-Abdely 2009; Andes and Casadevall 2013). In addition, multiple chitin synthases most likely are present in *E. rostratum*, which are also known to be important in the *W. dermatitidis* model for resistance to attack during infections by host immune systems and to antifungal therapeutic drugs, as well as cell wall strengthening and cell wall remodeling (Abramczyk *et al.* 2009). Additionally, the regulation of its development from a spore to a hypha is likely controlled by pathways similar to one or more of those already studied in *W. dermatitidis* (Ye and Szaniszlo 2000; Wang and Szaniszlo 2007; Liu *et al.* 2008). This last hypothesis is plausible because even though *W. dermatitidis* is usually identified as a black yeast species, it is, in the manner of *E. rostratum*, in fact a conidiogenous phaeoid mold with the additional growth characteristic of being polymorphic. The experimental examination of the variable morphology of *W. dermatitidis* has led to the discovery of numerous regulatory aspects of its well-defined blastic, apical, and isotropic growth modes that produce myriad black forms of yeast, hyphae, and sclerotic (muriform) growth, respectively (Szaniszlo 2002; Abramczyk *et al.* 2009). The results have confirmed that this one model produces most, if not all, of the many growth forms of most other phaeoid agents of disease and therefore should have developmental pathways in common with any and all of the developmentally less versatile species.

*W. dermatitidis* has been characterized as having remarkable thermotolerance, halotolerance, pH tolerance, and hydrotolerance (Zalar *et al.* 2011). Both thermotolerance and hydrotolerance are documented by the frequent isolation of *W. dermatitidis* from environments including hot tubs, saunas, steam baths, humidifiers, and dishwashers (Nishimura and Miyaji 1982; Matos *et al.* 2002; Zalar *et al.* 2011). In addition, this species was recently demonstrated to display enhanced growth induced by ionizing radiation (Dadachova *et al.* 2007; Robertson *et al.* 2012). This combination of tolerances is rarely observed in a single fungus and additionally makes *W. dermatitidis* an excellent system for studies of the extremophilic nature of fungi. Finally, it is important to note that no other phaeoid human pathogenic fungus besides *W. dermatitidis* is yet molecularly tractable and amenable to molecular transformation, targeted gene disruption, molecular gene transformation, and site-specific gene expression experiments (Abramczyk *et al.* 2009; Szaniszlo 2002).

The genome sequencing of *W. dermatitidis* provides the first opportunity to examine the large group of human-associated fungi of the order Chaetotheriales within the subclass Chaetotheriomycetidae of the Eurotiomycetes class of the Ascomycota. To expand the usefulness of the *W. dermatitidis* model, we describe here the first comparative genomic analysis of a phaeoid pathogen. We compare this genome to the most closely related sequenced fungi, including other pathogens, and find differences in gene content related to cell wall structure and secondary metabolite production. To characterize the genes involved in responding to low pH stress, we performed deep transcriptomic profiling and found that cell wall genes are significantly induced by this stress, suggesting that remodeling the cell wall is a major factor in the stress resistance in this organism.

## Materials and Methods

### Growth conditions for *W. dermatitidis*

Genomic DNA was prepared from the wild-type strain of *W. dermatitidis* 8656 (ATCC 34100, *Exophiala dermatitidis* CBS 525.76) cultured in YPD (2% peptone, 1% Bacto Yeast extract, and 2% dextrose) broth, with shaking at 200 rpm at 25° for 24 hr. For RNA-Seq experiments, wild-type cells were cultured in defined minimal medium (3 g/L NaNO_3_, 1 g/L K_2_PHO_4_, 1 g/L MgSO_4_ 0.7 H_2_O, 0.5 g/L KCl, 0.003 g/L thiamine, 5.3 g/L NH_4_Cl, 30 g/L glucose) adjusted to pH 6 and pH 2.5, respectively, with shaking at 25° for 24 hr. Total RNA was extracted with RiboPureYM-Yeast Kit (Invitrogen, Carlsbad, CA). For the light effect experiment, the wild-type and the *wdpks1* mutant with a disrupted polyketide synthase 1 gene were grown on either YPD agar or CDY (Czapek Dox plus 0.1% yeast extract) agar.

### Genome sequencing assembly and repetitive element identification

For genome sequencing, we constructed two libraries, 180 base fragment and 2-kb to 3-kb jumps, and sequenced both on the Illumina HiSequation 2000 platform. The 101-bp Illumina reads were assembled using ALLPATHS-LG (Gnerre *et al.* 2010) (build 18 May 2011, 09:01:19 R37280) with default parameters using approximately 100-fold depth of fragment reads and 50-fold depth of jumping reads. The assembly included 10 scaffolds totaling 26,350,763 bases and 238 contigs totaling 26,346,203 bases. Mitochondrial sequence was assembled in a separate 26-kb contig. Consensus *de novo* repetitive elements in the assembly were identified using Repeat Scout (Price *et al.* 2005) version 1.0.5; additional copies of consensus elements were identified in the assembly using RepeatMasker version open-3.2.8 (Smit *et al.*). Individual elements were classified based on BLAST to the NCBI nucleotide collection datasets (nr/nt) or to RepeatMasker reference sets (nucleotide and protein).

### RNA-Seq and annotation

For RNA-Seq, strand-specific libraries were constructed from polyA-purified RNA using the dUTP second strand marking method (Parkhomchuk *et al.* 2009; Levin *et al.* 2010) as previously described (Cuomo *et al.* 2012); 101-bp reads were generated on the Illumina HiSequation 2000 platform. RNA-Seq was assembled with the Inchworm component of Trinity (Grabherr *et al.* 2011) and processed with PASA (Haas *et al.* 2008) to generate a set of transcripts for gene prediction. An initial gene set was generated by using EvidenceModeler (Haas *et al.* 2008) to select the best gene call for a given locus from multiple gene prediction programs and PASA RNA-Seq transcripts as previously described (Haas *et al.* 2008, 2011). The RNA-Seq transcripts were compared to the EvidenceModeler gene set and missing genes were manually reviewed and edited when possible. Potential misannotated genes were also reviewed, including transcripts with ≥15 exons, introns ≥1000 bases, transcripts with Ns (uncertain bases), and CDS overlaps of >50 bases. Genes with similarity to repeats were identified by TPSI (http://transposonpsi.sourceforge.net/) or BLAST hits to known repeat proteins; high confidence repeats were removed from the gene set.

### Functional annotation and comparative analysis

We compared the genome of *W. dermatitidis* to those of 10 other related fungi, including *Aspergillus nidulans*, *A. niger*, *A. fumigatus*, *Coccidioides immitis, Trichophyton rubrum, Thielavia terrestris, Myceliophthora thermophila, Neurospora crassa, Saccharomyces cerevisiae*, and *Schizosaccharomyces pombe*. We first extracted protein sequences from all 11 genomes and generated functional annotations with InterProScan (Zdobnov and Apweiler 2001), Blast2GO (Conesa *et al.* 2005), and BLAST against KEGG and COG. Transporter family classifications were based on the top BLAST hit (expect less than 1e−15) to TCDB (Saier *et al.* 2009). Transmembrane regions were predicted using TMHMM (version 2.0) (Krogh *et al.* 2001) and signal peptide sequences were predicted using SignalP 4.0 (Petersen *et al.* 2011).

To build ortholog clusters, all protein sequences from the 11 fungi were compared using an all-against-all BLAST (requiring 1e−5), then clustered with the OrthoMCL2 pipeline (Li *et al.* 2003) to identify ortholog groups using default parameters. To infer a phylogeny for the 11 fungi, we first concatenated alignments of single-copy orthologs present in all 11 species; a phylogeny was estimated using RAxML (Stamatakis 2006) version 7.3.3 with 1000 bootstrap replicates and model PROTGAMMABLOSUM62. Syntenic regions were identified using DAGchainer (Haas *et al.* 2004) to compare linkage of orthologs.

To identify expansions of predicted functional domains, protein domain profiles were compiled for all 11 fungi compared using InterPro domain hits. Significant expansions of a particular InterPro domain in *W. dermatitidis* as compared to the total count in all other fungi were detected using the hypergeometric distribution using q-values (Storey and Tibshirani 2003) to correct for multiple testing (Table S1). Similarly, to identify transporter family expansions, transporter family counts in *W. dermatitidis* and the sum of the counts in other fungi were compared using Fisher exact test (which has a hypergeometric distribution), also using a q-value correction. The two largest transporter families [major facilitator superfamily (MFS) and amino acid/polyamine/organocation (APC)] were also classified based on conservation as follows: core clusters (present in each of four phylogenetic groups: *W. dermatitidis*; *Aspergillus* species; Onygenales (*T. rubum* and *C. immitis*); all other fungi analyzed); shared clusters that are present in at least two groups; and unique clusters that are only found within each group. Genes involved in functional pathways (Supporting Information, Table S2 and Table S3) were mapped by identifying orthologs of previously described gene sets for cell wall biosynthesis (De Groot *et al.* 2009), the cell wall integrity pathway (Futagami and Goto 2012; Levin 2005), the UDP–GlcNAc pathway (Milewski *et al.* 2006), the HOG pathway (Hohmann *et al.* 2007; Furukawa *et al.* 2005), the calcium/calcineurin signaling pathway (Rispail *et al.* 2009), and the pH signaling pathway (Andersen *et al.* 2009). Secondary metabolite gene clusters were identified using SMURF (Khaldi *et al.* 2010). Candidate gliotoxin biosynthetic genes in *W. dermatitidis* were identified based on ortholog mapping from the *A. fumigatus* gene cluster (Gardiner and Howlett 2005).

### Phylogenetic analysis

For GT2 and chitin synthases, proteins containing the GT2 domain were aligned with MUSCLE (Edgar 2004) and the phylogeny estimated with RAxML version 7.0.4 (Stamatakis 2006) with the PROTGAMMAWAGF model and 1000 bootstrap replicates. Phylogenies of the UNDG and chitin-synthase–like genes were inferred with RAxML version 7.3.3 with model PROTGAMMAWAG and 1000 bootstrap replicates. For UNGD/UGD classification, a maximum likelihood tree was inferred using MEGA5 (Tamura *et al.* 2011), with 1000 bootstrap replicates for UDP-N-acetylglucosamine 6-dehydrogenases (UNGD) UDP-glucose 6-dehydrogenases (UGD), and several experimentally characterized bacterial nucleotide sugar dehydrogenases. Previously classified bacterial sequences from the Integrated relational Enzyme database (http://ebi.ac.uk/intenz) (Fleischmann *et al.* 2004) were compared to fungal genes; these include one UDP-N-acetylglucosamine 6-dehydrogenase (UNGD; EC:1.1.1.136) from *Pseudomonas aeruginosa*, one UDP-N-acetyl-D-mannosamine dehydrogenase (UNMD; EC:1.1.1.336) from *Escherichia coli*, one UNMD from *Methanococcus maripaludis*, one GDP-mannose 6-dehydrogenase (GMD; EC:1.1.1.132) from *Pseudomonas aeruginosa*, and two UDP-glucose 6-dehydrogenases (UGD; EC:1.1.1.22) from *Pseudomonas aeruginosa*. Phylogenies of the UDP-glucose-6-dehydrogenase and GT1 genes were inferred using maximum likelihood with MEGA5 with 1000 bootstrap replicates.

### Differential gene expression using RNA-Seq

We used differential expression analysis scripts in the Trinity pipeline (Grabherr *et al.* 2011; Haas *et al.* 2013) to process RNA-Seq data generated from two pH conditions (pH 2.5 and pH 6), with three biological replicates from each condition. Briefly, we first extracted protein coding gene sequences from the *W. dermatitidis* genome sequence based on coordinates of gene models and added 100 bases of flanking sequence on each side to approximate UTRs. Then, the RNA-Seq reads from each of the six samples were aligned to the extracted coding sequences using bowtie (Langmead *et al.* 2009). The alignment files were used to quantify transcript abundances by RSEM (Li and Dewey 2011). Differential gene expression analysis was conducted using edgeR with TMM normalization (Robinson *et al.* 2009; Kadota *et al.* 2012) using a corrected p-value (Benjamini and Hochberg 1995) cut-off of 1e−10. Analysis of the data using MA plots did not show evidence of any apparent bias. To detect gene function enrichment under each of the two pH conditions, gene sets classified based on InterPro, COG, KEGG, and TCDB functional annotations were tested for enrichment using GSEA (Subramanian *et al.* 2005), calculating q-values to correct for multiple testing. We also tested manually curated gene sets responsible for cell wall biosynthesis (Table S2) or belonging to pathways involved in cell stress responses (Table S3).

## Results

### Generation of a highly complete genome and gene set

We generated a high-quality genome assembly that comprises a small number of scaffolds. We sequenced the genome of *W. dermatitidis* using Illumina technology and assembled with ALLPATHS. The assembly consisted of 10 scaffolds with N50 of 3.62 Mb, totaling 26.4 Mb, with only 0.02% of gap bases in scaffolds. Some scaffolds likely corresponded to complete chromosomes; the smallest two scaffolds were 7 kb and 255 kb, suggesting there are eight or fewer chromosomes ranging from approximately 1.2 to 4.2 Mb. In support of this, we identified a candidate telomeric repeat array at 10 of the scaffold ends; three of the scaffolds (1.2, 1.5, and 1.6) contained repeat arrays at both ends, as would be expected for complete chromosomes. A candidate telomeric repeat unit of “TTTAGGG” was identified that contained one additional base compared to the “TTAGGG” repeat found in telomeres of many eukaryotes including fungi. This new 7-mer repeat was present in 6 to 13 copies at the identified scaffold ends. Utilizing this highly complete assembly, a total of 9269 genes were predicted using a combination of gene prediction programs and strand-specific deep sequencing of RNA (RNA-Seq) (*Materials and Methods*). A total of 8826 genes (95.2%) were supported by RNA-Seq (FPKM ≥0.5).

*W. dermatitidis* shares the largest amount of syntenic gene blocks with other ascomycetes of the Eurotiomycetes class. An average of 3.4 Mb of *W. dermatitidis* sequence was found in syntenic regions with each of the three aspergilli examined (*Materials and Methods*), with *A. fumigatus* sharing the highest amount of syntenic regions and genes. A slightly lower total of 2.9 Mb of sequence was found in syntenic regions with the two Onygenales species examined; approximately half (53%) of the 612 genes in these syntenic regions were also found in syntenic regions shared by *W. dermatitidis* and the Eurotiales.

The assembly contained a small fraction of repetitive sequence, identified *de novo* based on sequence similarity to other regions of the genome (*Materials and Methods*). A total of 86 repeat consensus elements covered 2.9% of the genome. More than half of these sequences (64%) shared similarity with characterized transposable elements. Most of the identified transposable elements in *W. dermatitidis* were retroelements (84%), with a 3-to-1 ratio of Gypsy to Copia types. These are the most common types of LTR retroelements found in fungal genomes, and typically Gypsy elements have higher copy numbers (Muszewska *et al.* 2011).

Conservation of genes involved in mating and meiosis provides evidence for an as-yet-undetected sexual cycle (Schurko and Logsdon 2008). The MAT locus in the sequenced isolate of *W. dermatitidis* contains a HMG gene (HMPREF1120_08862) flanked by *SLA2* (HMPREF1120_08859) and *APN2* (HMPREF1120_08863), as in other Pezizomycotina ascomycetes (Lee *et al.* 2010). In addition, many of the genes defined as a core meiotic set are conserved in the genome; of 12 core meiotic genes, 9 are present in *W. dermatitidis*. The three missing genes, *DMC1*, *HOP2*, and *MND1*, are also missing from organisms with known sexual cycles, including *Caenorhabditis elegans* and *Drosophila melanogaster* (Schurko and Logsdon 2008). These three genes are present in all the Eurotiales and Onygenales compared here, suggesting a relatively recent loss of these three genes in the *W. dermatitidis* lineage.

### Gene conservation and gene family evolution

**Figure 1:**
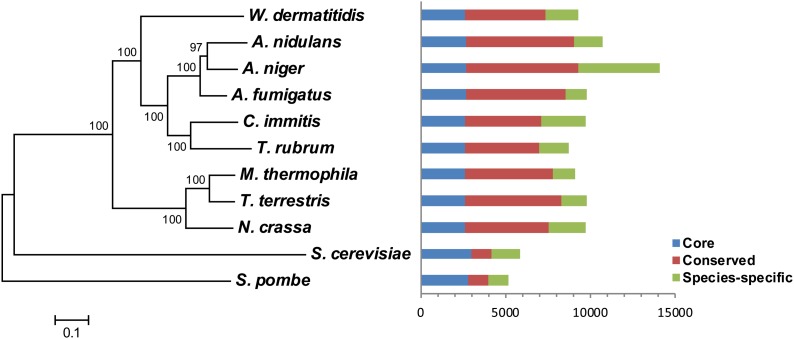
Phylogenetic relationship and gene conservation of *W. dermatitidis* and 10 compared fungi. Phylogeny (left panel) is based on concatenated MUSCLE alignments of 2013 single copy genes. Species phylogeny was inferred using RAxML (BLOSUM62 matrix and PROTGAMMA model) with 1000 bootstrap replicates. Ortholog conservation (right panel) highlights genes conserved in all species (core, blue), genes conserved in at least two species (conserved, red), and genes unique to a given species (species-specific, green).

We compared gene conservation between *W. dermatitidis* and related fungi to characterize the unique content of *W. dermatitidis*. Within the Eurotiomycetes, *W. dermatitidis* is the first sequenced representative of the black yeasts that is part of the subclass Chaetothyriomycetidae, whereas aspergilli and Onygenales are part of the Eurotiomycetidae. We compared *W. dermatitidis* to three aspergilli (*Aspergillus nidulans*, *A. niger*, and *A. fumigatus*) and two Onygenales (*Coccidioides immitis*, *Trichophyton rubrum*), as well as outgroup fungi representing thermophiles (*Thielavia terrestris*, *Myceliophthora thermophila*) and three well-studied ascomycete models (*Neurospora crassa*, *Saccharomyces cerevisiae*, *Schizosaccharomyces pombe*) (Figure 1). A total of 2626 *W. dermatitidis* genes were conserved across all 11 fungi compared, and a larger set of 4755 genes was conserved between *W. dermatitidis* and at least one other of the species examined. Despite the high divergence from the other fungi compared here, *W. dermatitidis* contained a similar number (1888) of species-specific genes as found in other fungi (Figure 1). These include 58 paralogous clusters (corresponding to 129 genes) specific to *W. dermatitidis*. Examining the species-specific genes, the most abundant protein domains identified belong to families of transporters (MFS and amino acid transporters), transcription factors, and short-chain dehydrogenases.

**Figure 2:**
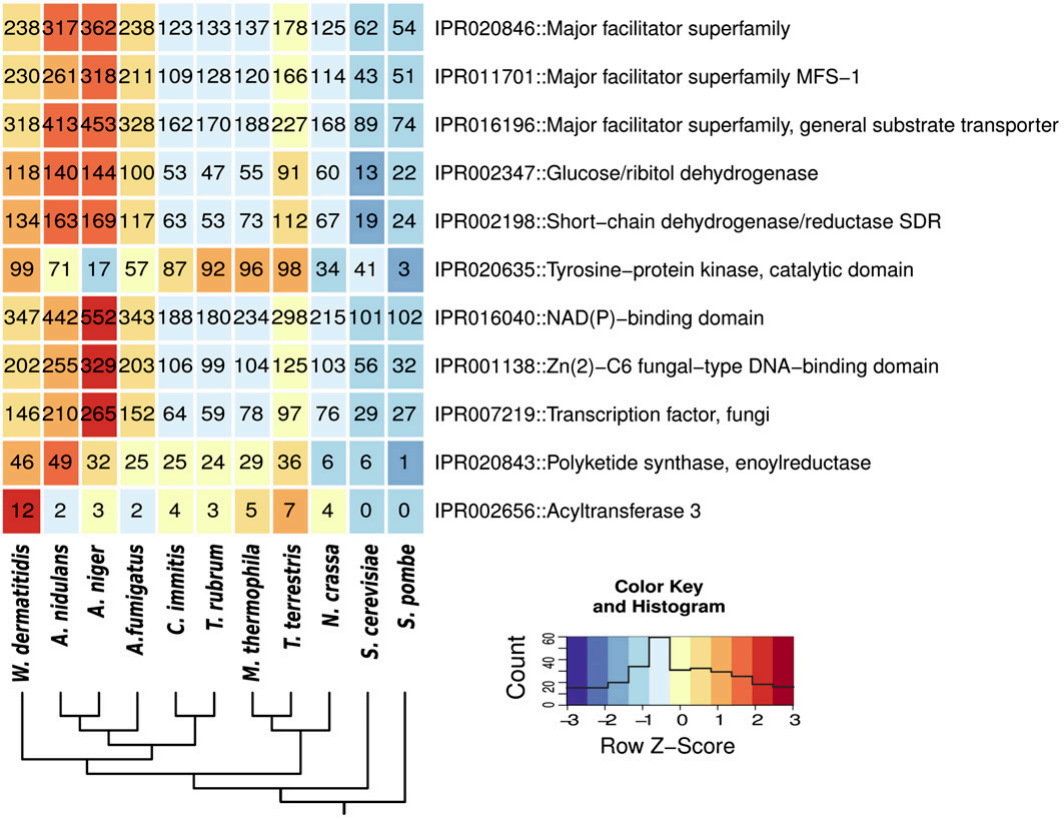
Protein domains enriched in *W. dermatitidis* in comparison to other fungi. Domain counts in *W. dermatitidis* were compared to the other 10 fungi and enriched domains were identified using the hypergeometric distribution. Domains are sorted by p-value and then grouped by category similarity. All domains shown are significantly enriched, with q-values <0.05.

To examine differences in predicted functional content, we identified protein domains enriched in *W. dermatitidis* compared to other ascomycete fungi. A total of 11 InterPro domains were enriched in the genome of *W. dermatitidis* (Figure 2). These included major facilitator type (MFS) transporters, saccharide dehydrogenases, kinases, transcription factors, enoylreductases (found in polyketide synthases and dehydrogenases), and acyltransferases. No InterPro domain families are significantly depleted in *W. dermatitidis* compared to those in the other fungi examined.

**Figure 3:**
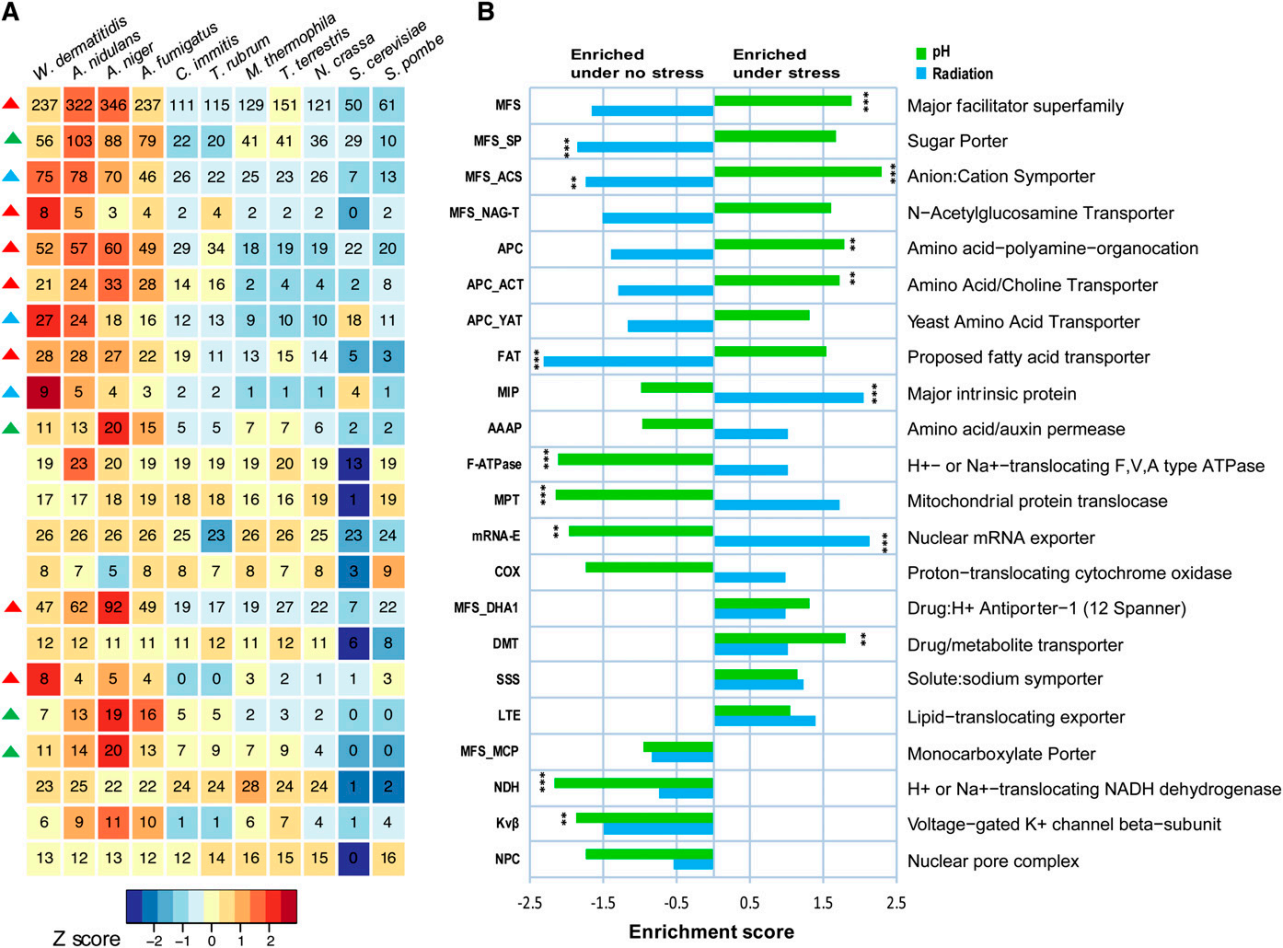
Transporter family expansion and expression enrichment under stress conditions. (A) Heatmap showing expansion of transporter families in *W. dermatitidis* compared to other fungi. Families that are significantly expanded (q-value <0.05) are marked with a triangle on the left of the heatmap as follows: red, detected in comparison between *W. dermatitidis* compared to other fungi excluding *Aspergillus* species; green, *W. dermatitidis* and aspergilli compared to all other fungi; and blue, *W. dermatitidis* compared to aspergilli. (B) Enrichment of transporter families under pH (green bars) or radiation stress (blue bars). Significant enrichments noted as **q-value <0.05; ***q-value <0.01.

To further examine transporter family expansions, we annotated transporter subfamilies in all 11 compared fungi using the curated transporter database TCDB (Saier *et al.* 2009). This analysis identified several transporter families that are significantly expanded (Fisher exact test, q-value < 0.05) in *W. dermatitidis*; these include the large MFS and amino APC transporter families, as well as sodium-dependent solute transporters, fatty acid transporters, and major intrinsic protein transporters (Figure 3). The MFS and APC transporter families are expanded in *W. dermatitidis* as well as in the three aspergilli relative to the other compared fungi (Figure 2). To examine if the expansions in *W. dermatitidis* and the *Aspergillus* species are independent, we analyzed the conservation of these two largest expanded families in *W. dermatitidis*, MFS (237 members) and APC (52 members) (*Materials and Methods*) (Figure S1). Conserved transporter clusters found in all species groups do not display expansions in *W. dermatitidis* or the aspergilli. In contrast, MFS and APC clusters conserved in a subset of species show significant copy number increases in these species (Figure S1A); transporter families shared between *W. dermatitidis* and *Aspergillus* species account for most of higher total number. For example, *W. dermatitidis* has a total of 41 MFS transporter clusters that are shared with one other fungal group, of which 33 are shared with only the aspergilli. In addition, both species contain a large number (50 in *W. dermatitidis* and 100 in the aspergilli) of species-specific transporter clusters. These comparisons suggest that both lineage-specific expansion and copies shared by only *W. dermatitidis* and the aspergilli duplications contribute to the MFS and APC transporter family expansions in these species.

A total of 12 acyltransferase proteins are found in *W. dermatitidis*, nearly twice the number as in all other analyzed fungi (Figure 2). Whereas four of the acyltransferases are conserved between *W. dermatitidis* and other fungi, the other eight copies appear species-specific. These proteins have between three and eight predicted transmembrane regions, suggesting they are likely embedded in the membrane. In eukaryotes, the function of this domain is unknown, but the membrane-embedded OatA acyltransferase from the pathogenic bacterium *Staphylococcus aureus* is suggested to provide resistance to lysozymes induced during early host defense response (Bera *et al.* 2006).

To identify genes that may be involved in pathogenesis and other unique phenotypic properties of *W. dermatitidis*, we compared the InterPro profile to other human-associated ascomycete fungi (Figure 2). Although found living on human skin, *W. dermatitidis* most commonly forms cysts or abscesses more deeply among living cells of cutaneous and subcutaneous tissues; *T. rubrum*, the cause of athlete’s foot, utilizes mainly the keratinized dead cells of the outermost surface of skin as well as the keratin of nail. To determine if there are any gene changes common to these two different types of dermal associations, we identified InterPro domains enriched in both *W. dermatitidis* and *T. rubrum*. Significant domain enrichments common to the two species include three classes of kinases: tyrosine; serine/threonine; and aminoglycoside phosphotransferases (Table S1). Major facilitator transporters and polyketide synthases are next most highly overrepresented. In addition, we found a depletion of domains involved in the degradation of plant components in *W. dermatitidis* (Table S1) similar to that found in Onygenales species (Sharpton *et al.* 2009; Desjardins *et al.* 2011; Martinez *et al.* 2012). Pectate lyase and cellulose binding domains are reduced in both fungi, although higher in number in *W. dermatitidis* than in *T. rubrum* (Table S1). The glycosyl hydrolase family 28 (GH28) involved in degrading pectin was present in 11 to 20 copies in our comparison group of aspergilli but was absent in *W. dermatitidis* as well as in *T. rubrum* and *C. immitis* (Table S1). The GH7 family involved in cellulose degradation was also absent from all three species but present in two to six copies in the other examined fungi (Table S1).

Unlike *T. rubrum* and *C. immitis*, *W. dermatitidis* does not contain a significant enrichment of any protease family genes (Table S1). Whereas GH family genes as a whole are less abundant in *T. rubrum* and *C. immitis*, these species both contain a subtilisin S8 protease expansion absent in *W. dermatitidis* (Table S1). The expanded family of subtilisins in *C. immitis*, which may play a role in keratin degradation, are fewer in number in *W. dermatitidis*; the subtilisin protease domain (IPR000209, Peptidase S8/S53, subtilisin/kexin/sedolisin) is found in only three genes in *W. dermatitidis* compared to 21 genes in *C. immitis*. Although an enhanced capacity to degrade proteins may compensate for the lack of saccharide degradation in some species (Desjardins *et al.* 2011), the substrate preference in *W. dermatitidis* appears intermediate between protease and carbohydrate specialists.

### Potential for secondary metabolite production

*W. dermatitidis* contains a subset of the secondary metabolite enzymes found in other fungi. There are only seven NRPS or NRPS-like proteins and four PKS or PKS-like proteins; no hybrid PKS-NRPS was identified. By comparison, aspergilli contain a larger portfolio of these enzymes, with an average number of proteins across eight species of 26 PKS or PKS-like, 28 NRPS or NRPS-like, 2 hybrid PKS-NRPS, and 7 DMATS (Khaldi *et al.* 2010). Of the 11 *W. dermatitidis* enzymes, 7 are widely conserved in at least 8 of the other species in our comparative set. Of the four others, three are found in all three aspergilli and one (HMPREF1120_03318) is specific to *W. dermatitidis*.

Of the nine potential NRPS or PKS clusters containing accessory genes, one NRPS (HMPREF1120_02993) cluster shares similarity with the gliotoxin cluster of *A. fumigatus*. In *W. dermatitidis*, the gliP-like NRPS is conserved along with seven of the linked genes in the *A. fumigatus* cluster, although they are highly rearranged in gene order in *W. dermatitidis* (Figure S2). This cluster is not highly expressed or regulated by pH; in contrast, a different NRPS gene (HMPREF1120_04809) had 11 of the 19 clustered genes, including the NRPS, repressed at low pH (corrected p-value <1e−10). Gliotoxin is produced by species of *Trichoderma* and *Penicillium*, in addition to species of *Aspergillus*, but has not been reported in *W. dermatitidis* or other black yeasts. The presence and expression of this cluster in *W. dermatitidis* suggest it is capable of producing gliotoxin or another similar molecule; the phytopathogen *Leptosphaeria maculans* produces a structurally similar molecule, sirodesmin, from a homologous gene cluster (Gardiner and Howlett 2005). The immunosuppressive properties of gliotoxin may explain the decreased virulence of *Aspergillus* gliP mutants (Sugui *et al.* 2007); the presence of this cluster in *W. dermatitidis* suggests it has the potential to play a similar role in this organism.

### Novel aspects of cell wall biosynthesis

Analysis of cell wall genes revealed potentially unique features of the *W. dermatitidis* cell wall. Chitin synthase genes, which belong to the CAZy glycosyl transferase GT2 family, encompass several different subfamilies involved in chitin generation. We identified all seven previously described chitin synthase genes in *W. dermatitidis* (Table S2). In addition to these seven *WdCHS* genes, we identified one novel chitin synthase-like gene (HMPREF1120_01791) with a significant (4e−23) match to the chitin synthase domain (PF03142). Based on a phylogenetic analysis of *CHS* genes, this novel *CHS*-like gene is most closely related to *CHS4* and *CHS5* but clearly separate from each group (Figure S3). This novel *CHS* has five predicted transmembrane regions, similar to the other known *CHS*. Further analysis of the conservation of this novel *CHS*-like gene is described below.

Although many of the genes involved in cell wall biosynthesis are conserved in *W. dermatitidis*, we found most of the genes involved in α-glucan metabolism are absent. Notably, we found that *W. dermatitidis* has a reduced set of proteins for 1,3-α-glucan synthesis and processing; the Ags family of 1,3-alpha glucan synthases and the Agn family of 1,3-alpha-glucanases are not present in the *W. dermatitidis* genome. In contrast, both the Ags and Agn families are present in multiple copies in *Aspergillus* species (Table S2). Another class of enzymes, alpha amylases, can perform transglycosylation or hydrolysis of glycosidic linkages; many fungi have intracellular and secreted alpha-amylases (Van Der Kaaij *et al.* 2007). A conserved intracellular alpha-amylase is present in *W. dermatitidis* (HMPREF1120_03460) and most other fungi analyzed. The secreted amylases appear more diverse; in *W. dermatitidis*, this protein (HMPREF1120_08319) does not contain a secretion signal and is missing two domains (DUF1966 and starch binding domain) that are conserved in other secreted alpha amylases. This pattern is also observed for *T. rubrum*; this species has also lost the Ags family of 1,3-alpha-glucan synthases, the Agn family of 1,3-alpha-glucanases, and the secreted alpha amylases. Only a single intracellular alpha-amylase is found in *T. rubrum*.

Proteins involved in the regulation of chitin synthase activity, the export of chitin synthases, and chitin degradation (chitinase) are all conserved, as are proteins involved in glucan synthesis and processing (Table S2), one of which has been previously described (Szaniszlo *et al.* 1985). Two chitin deacetylases are conserved; however, neither is predicted to be secreted in *W. dermatitidis*. In other species these enzymes are secreted and act to modify chitin in the cell wall. In addition, *W. dermatitidis* lacks copies of two conserved chitosanases, similar to *S. cerevisiae* and *S. pombe*. This shift in the content of cell wall proteins suggests that 1,3-α-glucan synthesis and processing in *W. dermatitidis* is likely reduced compared to that in the aspergilli and other filamentous fungi and suggest that cell wall composition may also vary for other components.

Examining the conservation of other genes involved in cell wall structure revealed expansions specific to *W. dermatitidis*. GH17 *trans*-glycosylases are highly conserved across the fungi we compared; in addition to the conserved copies, two additional paralogs are present in *W. dermatitidis* (Table S2). These extra copies could enable additional cell wall cross-linking, which may strengthen the cell wall and increase survival during stressful conditions. The GT2 glycosyl transferase family is also expanded in *W. dermatitidis*; 10 copies are present, which is significantly more than in the other fungi compared (hypergeometric test, q-value=0.11) and more than has been reported to date in any other genome. The aspergilli have the next highest count of seven GT2 genes. Phylogenetic and domain structure analysis revealed that the three GT2 genes (HMPREF1120_04699, HMPREF1120_5299, HMPREF1120_05696) contain a domain for cellulose synthase (IPR005150) (Figure S3). These proteins share sequence similarity with homologs in the three aspergilli we examined, including the celA gene (AN8444) of *A. nidulans*, which was previously suggested to play a role in β-glucan synthesis (De Groot *et al.* 2009).

Glucanases containing the GH16 module provide an example of domain shuffling and potential cell wall innovation. In *W. dermatitidis*, one GH16 gene (HMPREF1120_03145) also contains multiple LysM domains; the combination of these domains has not been previously detected. The LysM domain has recently been linked to pathogenesis in plant pathogens (De Jonge *et al.* 2010) and mycoparasites (Gruber *et al.* 2011), and it is highly expanded in dermatophytes (Martinez *et al.* 2012). In all other filamentous fungi, the LysM domain is found in combination with a GH18 domain; however, such a protein is not present in *W. dermatitidis*. Although the combination of domains found here is not necessarily pathogenesis-related, it again indicates that cell wall structure in *W. dermatitidis* is likely different, because the LysM could attach the GH16 to the cell wall, creating different linkages.

Other expanded domain classes include metabolic functions that may be involved in modifying the cell wall of *W. dermatitidis*. Glucose ribitol dehydrogenase, which breaks down sugar ends that lack alcohols, is found in high numbers in *W. dermatitidis* and aspergilli. Ribitol is found as a precursor in capsule formation in bacteria and may protect some fungi against osmotic stress (Pfyffer and Rast 1980).

### Horizontal transfer of two-gene cluster between fungi and algal viruses

**Figure 4:**
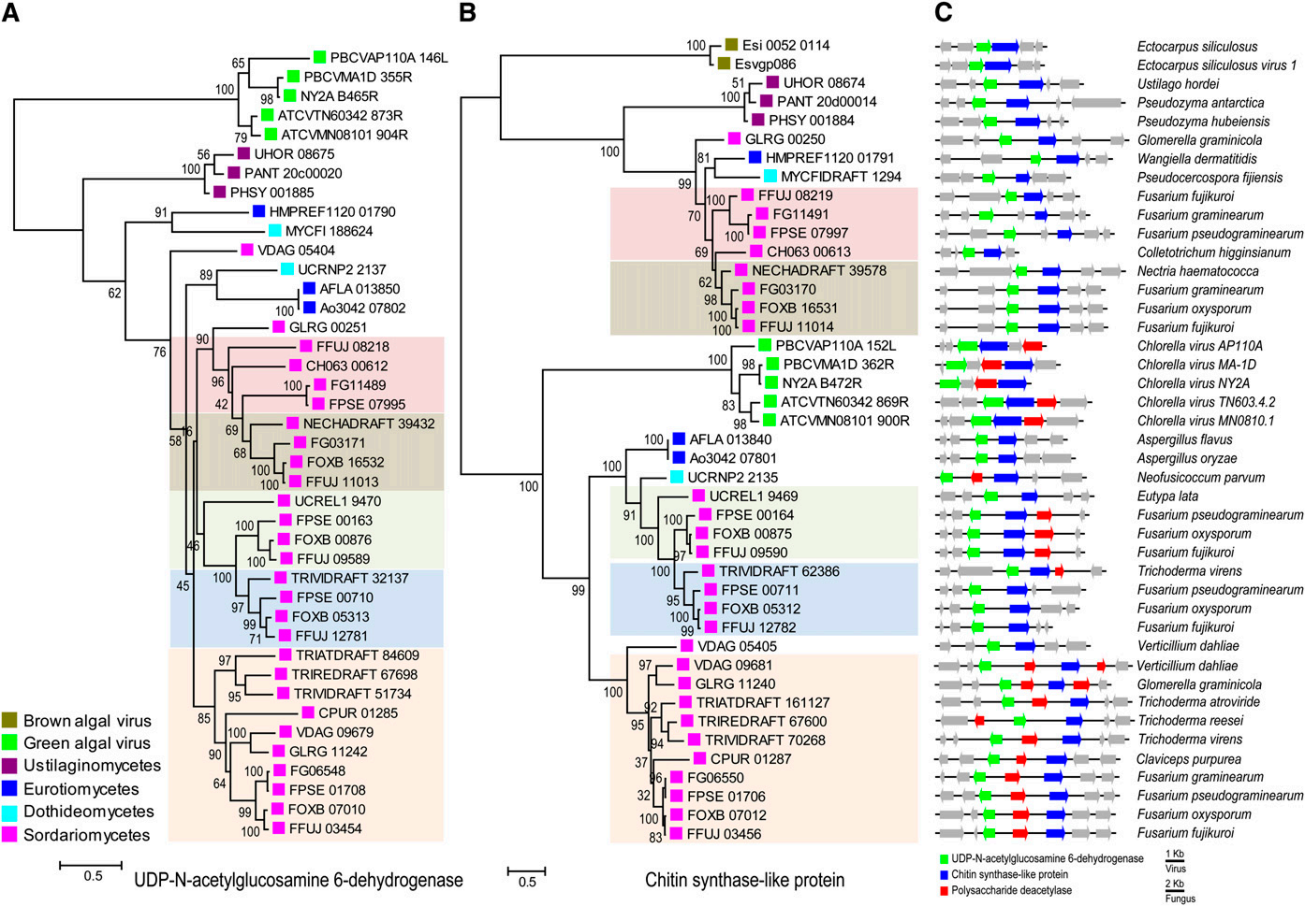
Phylogenetic relationship and linkage conservation of the UDP-N-acetyl-D-glucosamine 6-dehydrogenase (UNDG) (A) and chitin synthase-like (B) gene pair. Phylogenies (A and B) were estimated using RAxML (Stamatakis 2006) with model PROTGAMMAWAG and 1000 bootstrap replicates. Taxonomic classification is noted by the colored boxes in (A) and (B). This gene pair is mainly found in Ascomycetes, predominantly Sordariomycetes (pink box), but is also observed in Eurotiomycetes (blue), Dothidiomycetes (brown). Outside Ascomycota copies are conserved in two Basidiomycete species (both Ustillaginomycetes, maroon) and outside fungi in brown algal viruses (brown) and green algal viruses (green). Homologs of the UNDG in the brown algal viruses are too divergent for phylogenetic analysis. (C) Genome organization of the gene pair. These genes are adjacent in all genomes, including brown algal viruses, although the relative transcriptional orientation varies (arrow direction).

The novel *CHS*-like gene (CHSL) and the adjacent gene (HMPREF1120_01790), a predicted UDP-N-acetyl-D-glucosamine 6-dehydrogenase (UNGD) (Figure S4), form a two-gene cluster with an unusual conservation pattern. This gene pair may work in concert; UNGD family enzymes provide precursors for glycosyltransferase enzymes. Although this gene pair is not conserved in the other fungi we compared, both genes are frequently observed in species from the Sordariomycetes class, including *Fusarium fujikuroi*, *F. oxysporum*, and *F. graminearum*, two *Aspergillus* species (*A. flavus* and *A. oryzae*), and three Ustilagomycotina species within the Basidiomycota, including *Ustilago hordei* (Figure 4). These genes are also adjacent in these other fungal genomes, such as the gene pair FOXB_16531 and FOXB_16532 from *F. oxysporum* and UHOR_08674 and UHOR_08675 from *U. hordei*. This gene pair is present in multiple copies at unlinked loci in some Sordariomycetes; phylogenetic analysis suggests that lineage duplication events resulted in these extra copies (Figure 4). Whereas this gene pair is found in a subset of *Aspergillus* species, including *A. flavus* and *A. oryzae*, these genes are not part of a larger syntenic region shared with *W. dermatitidis*. The genes flanking this cluster are conserved within, but not between, species groups (Figure 4).

In addition to fungal species, this pair of linked genes is also found in several chlorella viruses (Figure 4), a group of large viruses infecting green algae (Jeanniard *et al.* 2013). A more distantly related gene pair is also found in the lysogenic brown algal phaeovirus EsV-1 and in the host *Ectocarpus siliculosus*, with a suggested function of either an alginate or a chitin synthase (Delaroque *et al.* 2001). These green and brown algal viruses belong to two distantly related genera of the family Phycodnaviridae. Phylogenetic analysis places both families of viral genes outside the fungal gene clade for UNGD; the CHSL proteins form two clades, each with a group of either brown or green algal viral sequences at the base. In the clade without *W. dermatitidis*, a third gene, a polysaccharide deacetylase, is part of the gene cluster in nearly all species including the green algal viral genomes (Figure 4C). This analysis suggests independent horizontal transfer of this gene cluster from two distantly related groups of viruses.

UNGDs and the related subfamily UDP-glucose 6-dehydrogenase (UGD; EC:1.1.1.22) belong to the UDP-glucose/GDP-mannose dehydrogenase family, which converts different UDP-sugar monomers to acidic forms. The UNDG class generates UDP-N-acetylglucosaminuronic acid (UDP-GlcNAcA) and the UGD class generates UDP-glucuronic acid (UDP-GlcA). We identified a candidate UGD protein in *W. dermatitidis* (HMPREF1120_03278) based on shared domain structure (NAD binding domain, a central domain and a UDP binding domain) and phylogenetic analysis (Figure S4). This UGD in *W. dermatitidis* is adjacent to a glycosyl transferase group 1 gene (GT1; HMPREF1120_03277). Searching the NCBI protein database with these two *W. dermatitidis* proteins identified conserved copies of this two-gene cluster in many Eurotiomycetes and Sordariomycetes species but in only a single Dothideomycete species. Phylogenetic trees are consistent between these paired UGDs and GT1 proteins (Figure S5), supporting a common origin of these two genes. The *A. fumigatus* ortholog, UGD1, is essential for the production of an acidic glycolipid not previously described for any fungal species (Fontaine *et al.* 2009). The conservation of orthologs of this gene in *W. dermatitidis* and other fungi suggests that they are also capable of producing acidic glycolipids.

### Conservation of iron acquisition pathways

Multiple iron transport pathways are present in the *W. dermatitidis*, enabling acquisition of iron from the host during infection. Ferroxidase and metalloreductase enzymes could provide reduced ferric iron to putative high affinity iron permeases. As in *A. fumigatus* (Haas 2012), the genes encoding the ferroxidase FetC (HMPREF1120_01590 and HMPREF1120_04510) and high-affinity iron permease FtrA (HMPREF1120_01589 and HMPREF1120_04509) are adjacent in the genome; notably, *W. dermatitidis* has two copies of this two-gene cluster. An alternative pathway for iron acquisition involves siderophores. The proteins involved in synthesis of the siderophore fusarine C (FsC) in *A. fumigatus* (Haas 2012) are all present in *W. dermatitidis*; the SidG protein capable of converting FsC to triacetylfusarine C is not conserved. For these siderophore genes, the genomic organization is only partially conserved between these species. Although the *SidD* and *SidF* genes are closely linked in both genomes (HMPREF1120_01440 and HMPREF1120_001438, respectively), the *SidA* and *SidC* genes are adjacent in *W. dermatitidis* (HMPREF1120_07635 and HMPREF1120_07636, respectively), whereas in *A. fumigatus SidI* and *SidC* are adjacent.

### Melanin biosynthesis pathways and regulation

Melanin is a significant component of the phaeoid fungal cell wall because it helps defend against diverse environmental stresses such as UV, radiation, and oxidizing agents. In pathogenic fungi, melanin has been implicated in virulence as well as defense against the host immune system (Nosanchuk and Casadevall 2003; Jacobson 2000). Three different pathways contribute to melanin production: the DHN–melanin pathway; the DOPA–melanin pathway; and the L-tyrosine degradation pathway.

The DHN–melanin biosynthetic pathway is the best characterized pathway for producing fungal melanin, a black pigment localized to cell walls (Wheeler *et al.* 2008). In aspergilli, several enzymes in this pathway are developmentally regulated because melanin is produced in conidia but not typically in hyphae (Youngchim *et al.* 2004), including a polyketide synthase (PKSP), scytalone dehydratase (ARP1), 1,3,6,8-tetrahydroxynaphthalene reductase (ARP2), an alpha/beta hydrolase (AYG1), and two multicopper oxidases (MCOs), a ferroxidase (ABR1), and a pigment MCO (ABR2) (Table 1). Homologs for all these proteins are identified in *W. dermatitidis*, including the known polyketide synthase (HMPREF1120_03173, WdPks1p) and the alpha/beta hydrolase *AYG1* (HMPREF1120_02312, WdYG1), both of which are required for melanin production in *W. dermatitidis* (Feng *et al.* 2001; Wheeler *et al.* 2008). A second candidate alpha-beta hydrolase similar to AYG1 was also identified (HMPREF_00377, WdYG2); this gene is more closely related to the *AYG1* orthologs in aspergilli. This suggests the functional copy of *AYG1* in this pathway has been recently replaced in *W. dermatitidis*. The *WdPKS1* gene is conserved in other Eurotiomycetes and in *N. crassa*; however, these represent PKSs involved in production of other secondary metabolites. Both Arp1 and Arp2 are present as a single ortholog in *W. dermatitidis*. In contrast, oxidase Abr2 and oxidase Abr1 each has multiple homologous genes in the genome (Table 1).

**Table 1:** Melanin biosynthesis pathway genes and their response to pH and radiation stress

**Gene**	***A. fumigatus****^a^*	***A. niger***	***W. dermatitidis****^b^*	**pH Shift***^c^*		**Radiation***^c^*		
				**Log_2_** **Fold Change**	**p-value**	**Log_2_** **Fold Change**	**p-value**	**Description**
**DHN–melanin pathway**								
Pks1	**Afu2g17600 (Pks1)**	An03g05440	**HMPREF1120_03173**	**2.46**	**7.13e−55**	0.53	4.12e−05	Polyketide synthase
	Afu4g00210 (EncA)	An04g09530						
	Afu4g14560	An09g05730 (FwnA)						
	Afu7g00160	An11g07310						
Ayg1	**Afu2g17550(Ayg1)**	An14g05350 (Ayg1)	HMPREF1120_00377	−0.39	8.55e−02	−0.12	5.26e−01	Abhydrolase
			**HMPREF1120_02312**	0.32	5.16e−02	0.67	7.33e−07	
Arp2	**Afu2g17560 (Arp2)**	An02g00220	**HMPREF1120_05939**	**1.25**	**9.27e−16**	0.18	1.97e−01	1,3,6,8-Tetrahydroxynaphthalene reductase
Arp1	**Afu2g17580 (Arp1)**	An08g09920	**HMPREF1120_07724**	0.74	3.10e−06	**1.27**	**8.13e−23**	Scytalone dehydratase
Abr2	**Afu2g17530 (Abr2)**	An01g13660 (McoB)	HMPREF1120_02828	**3.34**	**3.07e−84**	−0.17	2.43e−01	Fungal pigment MCO
	Afu1g15670	An01g14010 (McoA)	**HMPREF1120_05645**	**1.73**	**4.84e−29**	**−2.04**	**2.78e−54**	
		An03g03750 (McoC)						
		An04g10400 (McoO)						
		An05g02540 (McoP)						
Abr1	**Afu2g17540 (Abr1)** Afu5g03790 (FetC)	An14g05370 (BrnA) An01g11120 (McoE)	HMPREF1120_04510	−0.64	6.12e−03	−0.07	7.34e−01	Fungal ferroxidase
		An01g08960 (McoH)	**HMPREF1120_00173**	−0.48	3.80e−03	0.75	7.04e−08	
		An15g05520 (McoK)	HMPREF1120_01590	−0.41	1.78e−02	0.16	3.16e−01	
			HMPREF1120_03706	**1.14**	**8.11e−11**	0.21	1.49e−01	
			HMPREF1120_04536	1.27	1.62e−10	0.47	1.73e−03	
**DOPA–melanin pathway**								
melC2	Afu3g01070	An01g09220 (MelC2)	**HMPREF1120_05316**	0.37	2.33e−02	**−1.70**	**6.39e−41**	Tyrosinase
		An03g00280						
melO		An12g01670	HMPREF1120_07692	0.53	6.71e−03	−0.04	7.99e−01	
		An09g02980	**HMPREF1120_03345**	**2.69**	**5.35e−63**	**2.44**	**5.11e−80**	
			**HMPREF1120_04514**	**3.01**	**4.29e−77**	0.43	4.97e−03	
	Afu4g14490	An12g05810 (McoJ)	HMPREF1120_05865	0.79	1.08e−04	4.33	3.11e−03	Laccase
		An16g02020 (McoM)	HMPREF1120_00199	**2.81**	**1.65e−58**	0.06	7.28e−01	
		An11g03580 (McoD)	HMPREF1120_08116	−0.87	5.79e−08	**−1.81**	**1.76e−36**	
		An08g08450 (McoG)	HMPREF1120_08564	**4.90**	**1.38e−169**	0.42	2.40e−03	
		An05g02340 (McoF)	HMPREF1120_04578	0.89	2.29e−07	−0.82	4.58e−09	
		An01g00860 (McoN)	HMPREF1120_02754	−0.07	7.70e−01	0.52	3.27e−04	
		An18g02690 (McoI)						
**L-tyrosine degradation pathway**								
Tat	Afu2g13630	An02g05540	**HMPREF1120_02164**	−0.22	1.88e−01	**−1.96**	**5.90e−49**	Tyrosine aminotransferase
hppD	Afu2g04200	An11g02200	**HMPREF1120_05584**	**2.08**	**6.68e−40**	**−4.33**	**1.38e−208**	4-Hydroxyphenylpyruvate dioxygenase
hmgA	Afu2g04220	An11g02180	**HMPREF1120_03827**	0.12	4.99e−01	−0.16	2.76e−01	Homogentisate dioxygenase
fahA	Afu2g04230	An11g02170	**HMPREF1120_03825**	−0.14	4.29e−01	**−2.38**	**2.27e−68**	Fumarylacetoacetate hydrolase
maiA	Afu2g04240	An11g02160	**HMPREF1120_03438**	−0.15	4.25e−01	−0.89	2.78e−10	Maleylacetoacetate isomerase

a *A. fumigatus* genes that belong to the DHN–melanin gene cluster (Tsai *et al.* 1999) are in bold; other genes share sequence similarity with the DHN–melanin genes.

b *W. dermatitidis* genes that have expression level under both pH 2.5 and pH 6 higher than median expression of all genes (FPKM 29.5) are in bold.

c Log_2_ fold change and corrected p-values calculated by edgeR. Significant gene expression changes (corrected p-values <1E−10) from both pH and radiation experiments are in bold.

Despite the notable production of melanin by *W. dermatitidis*, the DHN–melanin pathway genes are not clustered in the genome (Table 1). The genes in this pathway are clustered in many other fungi, including *A. fumigatus*, *A. clavatus*, *Penicillium marneffei*, and *P. chrysogenum*, with some rearrangements in the cluster; however, the genes are not clustered in two aspergilli, *A. nidulans* and *A. niger* (Woo *et al.* 2010). Despite the lack of clustering, high transcript levels were detected for all genes in the pathway. Genes for each step of this pathway are highly transcribed under both pH conditions, with average expression of the entire pathway (FPKM 391.9 under pH 2.5 and FPKM 150.4 under pH 6) greater than the average expression of all genes (FPKM 114.8). Compared to radiation stress, low pH stress caused more extensive induction of the DHN pathway: 5 out of the 12 genes were upregulated (corrected p-value <1E−10) under pH 2.5; with elevated levels of radiation, only one gene is upregulated by the same cut-off (see *Transcriptional response to pH shift* section).

MCOs are over-represented in *W. dermatitidis*, including *ABR*-like pigment-related oxidases (see above) and laccases (Table 1). *W. dermatitidis* contains a total of 13 MCO genes. By comparison, *A. fumigatus* and *A. nidulans* have seven and eight copies, respectively, whereas *A. niger* has 16 copies. These MCO genes are classified into three subfamilies: fungal pigment MCO; fungal ferroxidase; and ascomycete laccase (Ramos *et al.* 2011). The expansion in *W. dermatitidis* involves recent paralogous duplications in all three groups (Table 1). Of the 13 MCOs in *W. dermatitidis*, 12 are predicted to be secreted. Eight are highly upregulated after a shift to low pH (corrected p-value <1E−4), suggesting that most of these copies could be functional.

In addition to DHN melanin, many fungi also produce black or brown melanins through the DOPA–melanin pathway (Langfelder *et al.* 2003) in which tyrosinases or laccases generate dopaquinone either through hydroxylation of L-tyrosine or through oxidation of DOPA. The dopaquinone can auto-oxidize and polymerize to form melanins. *W. dermatitidis* also uses the DOPA–melanin pathway for melanin production (Paolo *et al.* 2006) and contains high numbers of both laccases (described above) and tyrosinases. Of the four tyrosinase genes present in the genome, two are conserved with *Aspergillus* homologs; the other two copies appear unique to *W. dermatitidis* and are highly upregulated (more than 10-fold) under pH 2.5 stress (Table 1). Two laccases are also upregulated by more than four-fold; in combination with the high tyrosinase expression, this suggests increased production of DOPA melanin at low pH. Similar to the DHN pathway, the DOPA pathway genes are also highly expressed under both pH conditions, with average FPKMs of 276.6 under pH 2.5 and 92.1 under pH 6.

An alternative pathway to produce a third type of melanin, using the L-tyrosine degradation pathway to produce pyomelanin as a side-product (Keller *et al.* 2011; Schmaler-Ripcke *et al.* 2009), is also conserved in *W. dermatitidis* (Table 1). The two enzymes involved in this pathway are 4-hydroxyphenylpyruvate dioxygenase (hppD) and homogentisate dioxygenase (hmgA). The hppD converts 4-hydroxyphenylpyruvate to homogentisate, which can be converted to pyomelanin through oxidation and polymerization. Homogentisate can alternatively be degraded to other compounds by HmgA and two downstream enzymes, maleylacetoacetate isomerase (MaiA) and fumarylacetoacetate hydrolase (FahA). In response to low pH, we found that only hppD was significantly upregulated (more than four-fold); all other genes in this pathway did not significantly vary in transcript levels, suggesting that under low pH *W. dermatitidis* might increase pyomelanin production by selectively activating hppD in the L-tyrosine degradation pathway. In contrast to the results from pH experiments, most of the genes in this pathway were significantly downregulated under radiation stress; the hppD gene was repressed more than 20-fold in radiated cells (Table 1), suggesting *W. dermatitidis* was less capable of pyomelanin production under radiation stress.

### Light-sensing and pigment production

Fungi have multiple light sensors that respond to different light wavelengths. These include a phytochrome that senses red light, the white-collar complex that senses blue light, a cryptochrome/photolyase that responds to both blue and UVA light, and a fungal opsin that senses green light. Although these components are missing in Onygenales species including *T. rubrum* and *C. immitis* (Idnurm *et al.* 2010), the *W. dermatitidis* genome contains orthologs for each class of light sensing proteins (Table 2). Under radiation treatment, the UVA light sensor cryptochrome CryA gene (HMPREF1120_04357) is most significantly upregulated, with transcript levels increasing more than three-fold (Table 2) (Robertson *et al.* 2012).

**Table 2:** Light-sensing genes and carotenoid biosynthetic pathway genes

**Gene**	***N. crassa***	***W. dermatitidis***	**pH***^a^*		**Radiation***^a^*		
			**Log_2_** **Fold Change**	**p-value**	**Log_2_** **Fold Change**	**p-value**	**Description**
Phy-1	NCU04834	HMPREF1120_07867	−0.71	6.70E−06	−0.22	1.22E−01	Phytochrome, response to red light
Phy-2	NCU05790						
Wc-1	NCU02356	HMPREF1120_06318	−0.16	3.71E−01	0.16	2.68E−01	White-collar complex, response to blue light
Vvd	NCU03967						
Wc-2	NCU00902	HMPREF1120_04504	−0.06	7.70E−01	0.42	2.18E−03	
Phr	NCU08626	HMPREF1120_04357	−0.18	3.69E−01	**1.62**	**4.73E−29**	Cryptochrome/photolyase, response to blue and UVA light
Nop-1	NCU10055	HMPREF1120_00264	0.84	8.65E−08	0.11	4.35E−01	Fungal opsin, green light–sensing
LaeA	NCU00646	HMPREF1120_06677	−0.85	1.06E−06	**−1.09**	**2.29E−14**	Velvet complex (VelB/VeA/LaeA), coordinates development and secondary metabolism in response to light
VeA		HMPREF1120_03349	0.25	1.38E−01	−0.01	9.76E−01	
VelB	NCU02775	HMPREF1120_00072	−0.96	9.76E−10	0.18	2.12E−01	
VelC	NCU07553	HMPREF1120_05878	0.20	2.64E−01	0.09	5.80E−01	Velvet family protein with homology to VeA
		HMPREF1120_02378	1.29	3.52E−08	0.00	1.00E+00	
VosA	NCU05964	HMPREF1120_06091	0.85	5.08E−04	0.74	1.54E−04	
CarO	NCU01735	HMPREF1120_02861	0.67	2.19E−04	0.53	3.16E−03	Opsin-like protein
		HMPREF1120_06324	−0.59	1.49E−01	0.10	7.02E−01	
CarB	NCU00552	HMPREF1120_02862	−0.94	3.60E−09	**2.35**	**9.29E−71**	Phytoene dehydrogenase
CarRA	NCU00585	HMPREF1120_02863	0.23	1.88E−01	**1.98**	**7.98E−53**	Carotene cyclase
CarX		HMPREF1120_02864	0.73	1.58E−05	0.44	1.17E−03	Carotenoid oxygenase

a Log_2_ fold change and corrected p-values calculated by edgeR. Significant gene expression changes (corrected p-values <1E−10) from both pH and radiation experiments are in bold.

The velvet family of regulatory proteins (VeA, VelB, VelC, VosA) plays a key role in coordinating development and secondary metabolism in response to light. In *A. nidulans*, VeA was found to interact with the light sensor complex (FphA-LreB-LreA) (Bayram *et al.* 2008). VeA can also form the heterotrimeric velvet complex with VelB and LaeA (VelB-VeA-LaeA), which regulates development and secondary metabolism. VelB and VosA can form a heterodimer (VosA-VelB) that represses asexual development during vegetative growth or in the absence of light. Orthologs for all of these proteins are found in *W. dermatitidis* (Table 2). *W. dermatitidis* has one extra copy of the VelC; these two proteins share the velvet factor domain (PF11754), but only one copy shares orthologs with the other fungi compared in our analysis.

**Figure 5:**
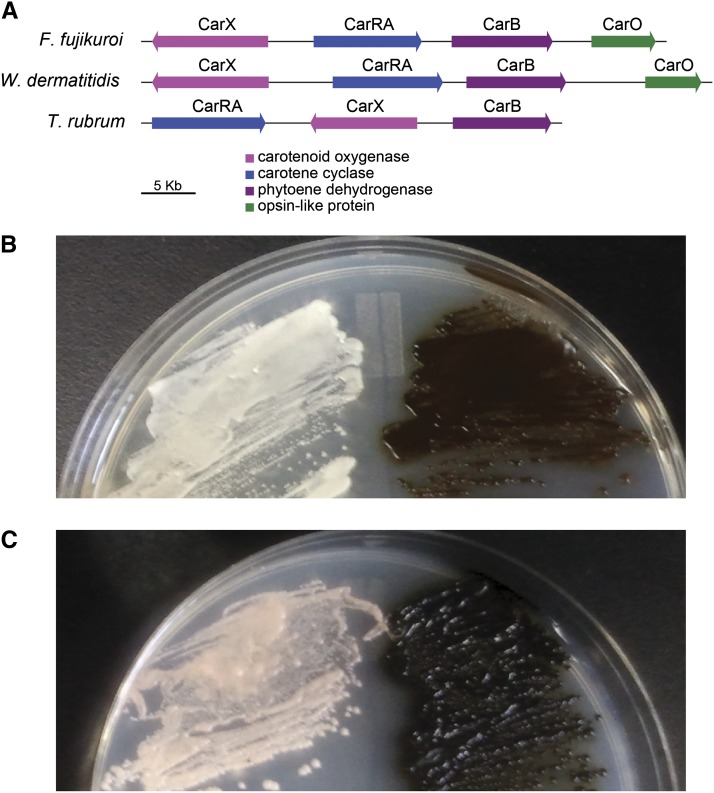
Effect of light on pigment production for *W. dermatitidis*. (A) Carotenoid biosynthesis gene cluster. All four genes are part of a single cluster in *W. dermatitidis* and in *F. fujikuroi*. The *T. rubrum* cluster does not include the CarO opsin protein, which is found at a different genomic location. (B and C) Wild-type (right sectors) and melanin defective mutant *wdpks1*Δ (left sectors) were incubated at 25° in the absence (B) or presence (C) of light for 2 weeks and grown on nutrient-limited CDY agar. Melanin (brown or black pigment) shown in the wild-type strain and carotenoid (pink pigment) shown in *wdpks1*Δ are both induced under light.

In addition to these photosensory proteins, an opsin-like protein is conserved within a carotenoid biosynthetic four-gene cluster (Table 2 and Figure 5). The carotenoid pathway genes are not always clustered; in some other species such as *F. fujikuroi*, all genes are adjacent (Thewes *et al.* 2005) but in other species only subsets are clustered. In *T. rubrum*, one locus contains three genes, with an inversion event relative to *W. dermatitidis* and *F. fujikuroi* (Figure 5); the opsin gene is found on a different scaffold in the *T. rubrum* assembly. In *W. dermatitidis*, carotenoid pigments are involved in light reception and also protect against oxidative damage (Geis and Szaniszlo 1984) and ionizing radiation (Robertson *et al.* 2012). The conservation of this cluster in *W. dermatitidis* is striking because the melanins produced by this fungus also serve similar roles. Exposure to light stimulates biosynthesis of melanin and carotenoid. Colonies of the wild-type of *W. dermatitidis* show darker pigmentation under light than those grown in the dark, and colonies of the melanin-defective mutant *wdpks1* show an increased pink color under light, indicating carotenoid production (Figure 5). The coordination of these responses would enable protection and response to light-related stimuli.

### Transcriptional response to pH shift

**Figure 6:**
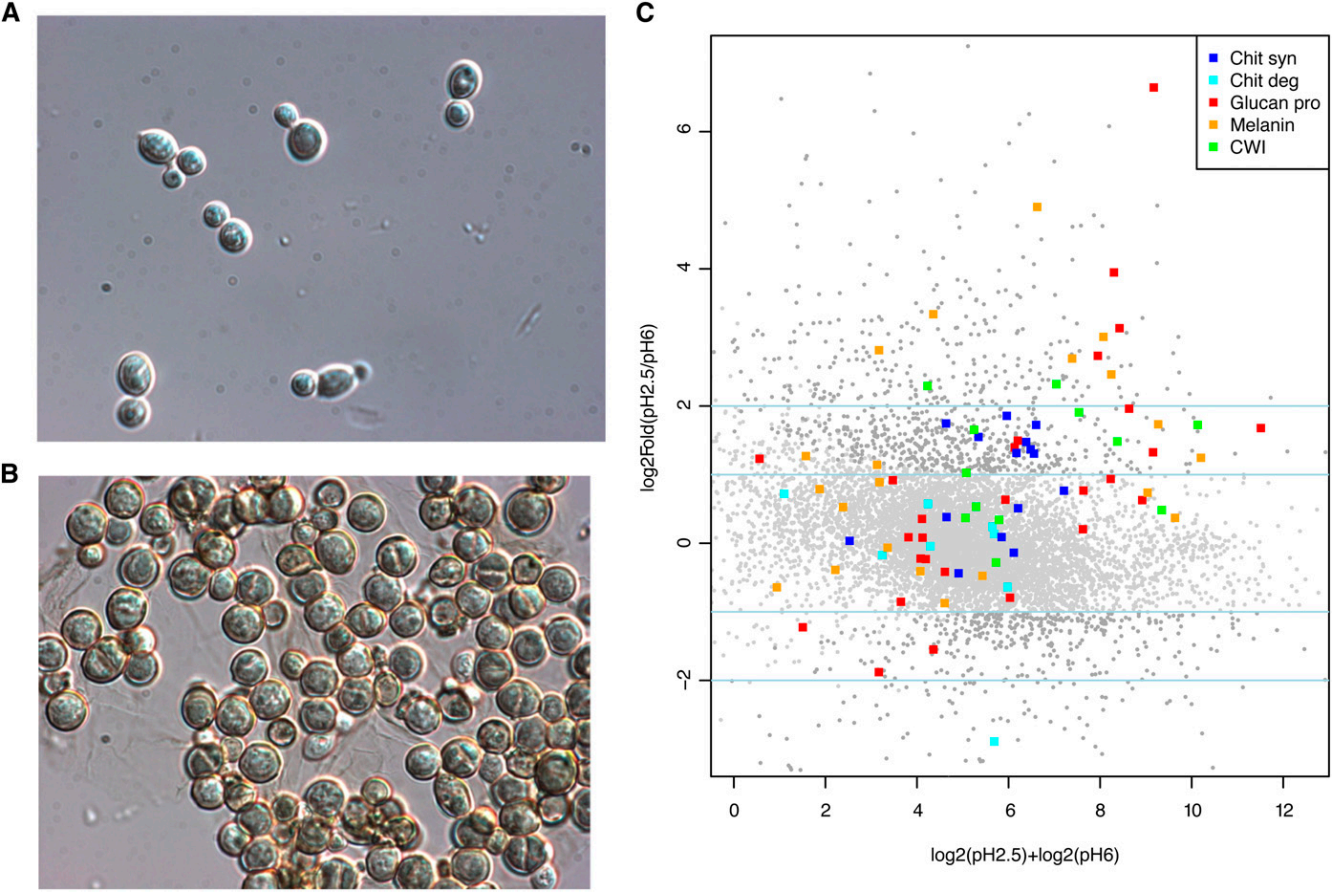
Response of *W. dermatitidis* to low pH. (A and B) Light microscopy of *W. dermatitidis* at pH 2.5 (top panel, magnification 1200×) and pH 6.5 (lower panel, magnification 1600×). (C) Functional category enrichment measured by GSEA. The x-axis of the MA-plot measures the log fold difference, and the y-axis shows log fold change between the two pH conditions. Four categories (chitin synthase, glucan processing, melanin, and cell wall integrity) of cell wall–related genes are significantly induced at low pH; one displayed category (chitin degradation) is not significantly induced.

Previous studies demonstrated that stress conditions induce morphological changes in *W. dermatitidis* cells. For example, under pH 2.5, the yeast form grows isotropically into enlarged forms that may become multicellular with unique characteristics, such as being multinucleate, having thick cell walls, and exhibiting melanin overproduction (Figure 6) (Szaniszlo *et al.* 1993). We analyzed gene expression under two pH conditions, pH 2.5 and pH 6, to examine how *W. dermatitidis* responds to acidic pH stress. For each pH condition, RNA was prepared for three biological replicates and expression levels measured using RNA-Seq. The biological replicates have highly correlated expression values, with an average Pearson correlation coefficient between replicates of 0.98 for pH 2.5 and 0.99 for pH 6. There is also high correlation between the two pH conditions, from 0.90 to 0.94, suggesting many genes are similarly expressed in both conditions. By comparing the RNA-Seq from the two pH conditions using edgeR (Robinson *et al.* 2009), a total of 1291 genes were identified as significantly differentially expressed (corrected p-value <1e−10) (*Materials and Methods*). These include 832 upregulated and 459 downregulated genes at pH 2.5 relative to pH 6.

Analysis of the differentially expressed genes suggests that the major responses to low pH are to strengthen the cell wall, to pump out cations, and to slow metabolism (Table S3 and Table S4). To identify functional enrichment under low pH stress, we ran gene set enrichment analysis (GSEA) (Subramanian *et al.* 2005) using gene sets defined by protein domains, KEGG pathways, transporter families, and other custom sets (*Materials and Methods*) (Table S3 and Table S4). Genes induced under low pH stress include several involved in cell wall functions, including cell wall biosynthesis (including chitin and glucan processing and melanin biosynthesis) but not chitin degradation (Figure 6C and Figure S6**)**. The cell wall integrity pathway, including the transmembrane sensor and sensor-transducer proteins, is highly upegulated; in contrast, none of the genes in the high osmolarity glycerol signaling pathway changes significantly in response to lowered pH (Table S4). Transporters (both MFS and APC) are also induced under low pH stress; the anion-cation symporters are most highly induced. Cytochrome p450 proteins, a diverse set of oxidizing enzymes, are also highly induced by pH and were previously noted to be highly induced by radiation stress (Robertson *et al.* 2012). Other significantly induced sets include melanin pathway genes, transcription factors, and aspartate proteases. For genes repressed under pH 2.5, enriched categories include housekeeping functions, such as mRNA export proteins. This may reflect slower growth of these cells at low pH levels.

The upregulation of cell-wall–related genes helps explain previous findings that *W. dermatitidis* cells develop thicker cell walls in low pH (Szaniszlo 2002, 2006; Szaniszlo *et al.* 1978). Most of the genes involved in chitin synthesis are significantly upregulated under low pH stress. Five out of the seven known chitin synthases are induced 2.5-fold to 3.6-fold under pH 2.5 (Table S2). In contrast, genes involved in glucan synthesis are not as significantly upregulated as chitin synthesis genes. In addition, genes involved in glucan modification are significantly upregulated under pH 2.5. For example, a glycosyl hydrolase 17 gene (HMPREF1120_08078) homologous to the *S. cerevisiae* putative glucanase *SCW11* is upregulated approximately 100-fold at pH 2.5. This supports previous results that showed that under low pH stress, cells produce thicker cell walls by upregulating cell wall biosynthesis genes, and that cells are enriched with chitin and reduced in glucans under low pH stress (Szaniszlo *et al.* 1983).

Calcium concentration plays a critical role in regulating cellular growth and polymorphism, and transporters appear induced at low pH. Both acidic stress and depletion of Ca^2+^ at neutral pH induce a transition of yeast form cells to isotropic or multicellular forms (Karuppayil and Szaniszlo 1997). In accordance with the phenotypic data, three genes encoding calcium transporters (HMPREF1120_08350, HMPREF1120_05235, and HMPREF1120_04272) are significantly upregulated under pH 2.5. These proteins belong to three different superfamilies: voltage-gated ion channels, Ca^2+^/Cation antiporter, and P-type ATPase, respectively. The increased transcription suggests that under acidic growth condition, cells actively regulate Ca^2+^ in response to low pH stress using these calcium transporters and also confirms at the molecular level that calcium is a critical factor for controlling cellular morphogenesis in *W. dermatitidis*.

At pH 2.5, cells grow isotropically and eventually produce multicellular forms (containing multiple nuclei and septa in one cell). The APSES transcription factor is a regulator of yeast–hyphal transitions and is required for hyphal formation in *W. dermatitidis* (Wang and Szaniszlo 2007). This gene (*WdSTUA*, HMPREF1120_05541) is conserved in *A. nidulans* (*stuA*, ANID_05836), *N. crassa* (*Asm-1*, NCU01414), *S. cerevisiae* (PHD1,YKL043W; SOK2,YMR016C), as well as three other genomes in our data set (*T. rubrum*, *C. immitis*, and *A. fumigatus*). At low pH, the transcript level of *WdSTUA* is significantly induced (corrected p-value=1e−7) by nearly two-fold. Fungal morphogenesis also requires motor proteins, including kinesins and myosins; seven kinesins, one dynein, and four myosins were found in the *W. dermatitidis* genome. The myosin 17 family includes two chitin synthases (Chs5p and Chs7p) with myosin head motor domains. Among these motor proteins, the kinesin-7 (KipA, HMPREF1120_04488) and the two Chs proteins with motor domains (Chs5, HMPREF1120_08776; Chs7, HMPREF1120_08777) are significantly upregulated (corrected p-value <4e−17) under low pH. Under radiation, when cell morphology does not shift to isotropic growth, most of these genes are significantly downregulated and only Kinesin-3 (*UncA*, HMPREF1120_07465) is significantly upregulated (corrected p-value=4.5e−9).

## Discussion

This analysis of the genome and transcriptional response to pH of *W. dermatitidis* is enabled by a high-quality assembly and gene set. Most of the eight large assembled scaffolds likely correspond to complete chromosomes; pulsed field gel analysis has suggested a karyotype of four chromosome bands, of which two appear to be doublets (Zheng *et al.* 2006). Supporting this, the sequence of just more than half of the ends of the eight large scaffolds consists of a newly identified telomeric repeat array. The three scaffolds with repeat arrays at both ends are likely complete chromosomes. In addition, there are a few internal gaps in this assembly. Gaps between contigs occupy only 0.02% of the total scaffold length. The deep-coverage RNA-Seq provided evidence for a highly complete and accurate gene set.

Although many other fungal genomes have been sequenced to date, this analysis represents the first detailed genomic analysis of a member of the Chaetotheriomycetidae subclass of fungi. Within the Eurotiomycetes class, distantly related fungi include aspergilli and Onygenales species, although *W. dermatitidis* and other related black fungi show a closer relationship to the fungi in some lichens (James *et al.* 2006). The sequencing of additional genomes within the Chaetotheriomycetidae and related lichen species will enable more detailed comparisons of how this group evolved in very different ecological niches.

The transcriptional response to pH has also been investigated for other fungi, including *A. niger* (Andersen *et al.* 2009) and *C. albicans* (Bensen *et al.* 2004). These studies have suggested that the pal/pacC pH signaling pathway is induced under alkaline pH conditions. Consistent with this, we found that the pal/pacC pathway in *W. dermatitidis* is not induced under pH 2.5 (Table S3), suggesting that this pathway is most likely specific for more neutral and alkaline pH response and is conserved in many fungi. In contrast, pathways that respond to low pH stress have not been well-defined. In this study, we identified several cell wall stress response pathways in *W. dermatitidis* based on ortholog mapping, including the cell wall integrity pathway (CWI), HOG signaling pathway (HOG), and Ca^2+^/calcineurin signaling pathway. We also included the pathway that synthesizes UDP–GlcNAc (from fructose-6-phosphate), the substrate for chitin synthesis. Examining the expression changes of these pathways under low pH stress highlights that the CWI pathway is the major stress response pathway induced by low pH stress; the entire pathway is induced, with only the exception of Rom2, which is slightly downregulated (Table S3). Seven out of the 12 genes are upregulated by approximately two-fold or more (corrected p-values <1E−10). The HOG pathway is also upregulated, but none of these genes has a significance level of corrected p-values <1E−10. The UDP–GlcNAc pathway is also slightly upregulated, consistent with our finding that chitin synthesis activity is upregulated under low pH (Table S2). Six out of the seven known chitin synthases, as well as genes involved in regulating chitin synthase activity, are induced, and genes involved in chitin degradation are repressed (Table S2). This suggests that *W. dermatitidis* cells increase their chitin content under low pH stress. Genes involved in beta-glucan processing and modification are also upregulated, including the GAS family of beta-1,3-glucanosyltransferases (HMPREF1120_07283, HMPREF1120_01763, and HMPREF1120_03477, orthologs of yeast GAS family) that are involved in cell wall modification and maintenance.

Whereas the genes involved in the DHN–melanin pathway are present in *W. dermatitidis*, they are not clustered in the genome as they are in many other fungi. In some species, including *A. fumigatus*, *P. marneffei*, and *Alternaria brassicicola*, the DHN–melanin pathway genes are clustered (Woo *et al.* 2010; Tsai *et al.* 1999; Kimura and Tsuge 1993). One possible explanation for the existence of dispensable metabolic pathway gene clusters is that the close linkage of pathway genes may benefit the regulation of pathway expression (Keller and Hohn 1997). However, break-up of gene clusters is not uncommon in fungi, which may reflect the lack of selection pressure required to maintain the cluster or to signal the first step in the eventual loss of the pathway (Keller and Hohn 1997). Constitutive production of the high amount of DHN–melanin throughout the life cycle of *W. dermatitidis* suggests that clustering may not be required for coordinated regulation of the genes in this pathway. However, upregulation of melanin biosynthesis genes and a number of cell wall genes under pH 2.5 indicates coordination of different pathways. Thickening cell wall makes more space available for deposition of increased melanin. This synergic effect would allow melanized fungi to survive and to adapt better to extreme environments.

In the context of the redundancy and importance of melanin for *W. dermatitidis*, the conservation of a complete set of light-sensing genes might seem paradoxical. Although surface melanin may shield some proteins from detecting light, the fact that light induces pigments such as DHN–melanin in its cell wall and carotenoid pigment in its membranes (Figure 5) suggests that light is able to penetrate these pigments and can still be sensed by the photoreceptor proteins. Alternatively, carotenoid pigments are more important at cell stages with lower melanin levels, such as during rapid growth at neutral pH. Therefore, overproduction of pigments in response to light is likely not shielding light but rather serving as scavengers to protect against oxidative damages caused by light, or is involved in processes known to require light in other fungi, such as sexual reproduction, phototropism, and conidiation (Purschwitz *et al.* 2006). Because melanin and carotenoid are examples of secondary metabolites, it will be interesting to further investigate the effect of light on other secondary metabolism pathways, such as the one for the gliotoxin-like cluster identified in this genome. A previous study demonstrated that ionizing radiation also significantly upregulated gene expression of cryptochrome CryA, a well-known photoreceptor (Robertson *et al.* 2012). This suggests that this photosensory protein may also sense radiation. The identification of a complete set of fungal photosensory genes in *W. dermatitidis* suggests that, beyond the filamentous fungus *N. crassa*, this black yeast is another excellent model organism to study fungal photobiology in addition to radiation biology.

Nucleotide sugar dehydrogenases catalyze NAD/NADP–dependent oxidation of nucleotide-linked sugar monomers to produce acidic nucleotide sugars, and different subfamilies of these enzymes recognize different nucleotide sugars. We identified UDP-glucose dehydrogenase (UGD) and UDP-N-acetyl-D-glucosamine 6-dehydrogenase (UNGD) in *W. dermatitidis* genome; each gene is adjacent to a glycosyltransferase gene (GT1 family and GT2 family, respectively). UGDs convert UDP-glucose (UDP-Glc) to UDP-glucuronic acid (UDP-GlcA) and UNGDs convert UDP-N-acetyl-D-glucosamine (UDP-GlcNAc) to UDP-N-acetyl-D-glucosaminuronic acid (UDP-GlcNAcA). In bacteria, UDP-GlcA and UDP-GlcNAcA are used for the synthesis of exopolysaccharides (EPSs) and lipopolysaccharides (LPSs) that are critical to bacterial virulence. The bacterial UNGD proteins are involved in the cell surface antigen biosynthesis process and are often located in gene clusters (Miller *et al.* 2004; Zhang *et al.* 2006) that also contain a GT family gene (Larkin and Imperiali 2009). In fungi, UGDs and UNGDs have not been well-characterized. The *A. fumigatus* UGD1 is the only one known so far that has been shown to be essential for the production of an acidic glycolipid (Fontaine *et al.* 2009). Conservation of the UGD-GT1 gene pair in many Eurotiomycetes and Sordariomycetes suggests these fungi can also produce acidic glycolipid in their cell membrane. UDP-GlcNAc is synthesized from fructose-6-phosphate through a four-gene pathway (Milewski *et al.* 2006), which is conserved in *W. dermatitidis* (Table S3). UDP-GlcNAc is the substrate for chitin synthases to make cell wall chitin; this substrate is also used for GPI-anchor biosynthesis (Kinoshita and Inoue 2000) and glycosylation of cell wall proteins (Jin 2012). However, the role of the acidic form (UDP-GlcNAcA) in fungal cells is unclear at present. Under low pH stress, six of the seven known chitin synthases are upregulated; however, the UNGD and the CHSL genes are downregulated, with similar fold change under low pH and radiation stress (Table S2), suggesting that this gene pair may not function to produce cell wall inner layer chitin. Instead, they may function to modify cell surface structures during infection.

The conservation of the UNGD-CSHL gene cluster in some fungi and algal viruses suggests horizontal transfer may have occurred between them. Previous work has shown that chlorella viruses have acquired and transferred genes involved in chitin metabolism to their algal hosts (Blanc *et al.* 2010). Although their association with fungi has not been previously suggested, the conservation of the UNGD-CSHL gene pair suggests these viruses may interact directly with these fungal species. The presence of this gene pair in two groups of Phycodnaviridae would have required independent transfer events from these two viral groups.

Analysis of the *W. dermatitidis* genome suggests a shift in cell wall composition relative to related ascomycete fungi. The absence of enzymes involved in 1,3 alpha-glucan synthesis and processing suggests this component is likely absent or highly reduced from the cell wall. This is in accord with a previous study examining cell wall polysaccharides, which noted the absence of alpha-glucan and chitosan from yeast and sclerotic bodies of *W. dermatitidis* (Geis 1981). This work also found that alpha-mannan formed the outer layer of both cell types, suggesting that these polysaccharides mediate surface interactions.

Variation in cell wall composition predicted by the genome of *W. dermatitidis* likely affects interactions with the host immune system. In *Histoplasma capsulatum* and *Magnaporthe oryzae*, α-glucan helps protect cells from detection by the innate immune system (Rappleye *et al.* 2007; Fujikawa *et al.* 2012). In *H. capsulatum*, α-glucan may help mask β-glucans from detection by the mammalian receptor dectin-1. An alternative mechanism for masking β-glucans has been shown in *A. fumigatus*, in which hydrophobins help block immune cell recognition (Aimanianda *et al.* 2009). Chitin can also block host immune recognition in *Candida albicans* (Lee *et al.* 2012; Mora-Montes *et al.* 2011). Because *W. dermatitidis* appears to have lost the capacity to produce α-glucan, these cells may rely on chitin or a similar set of hydrophobin-like proteins to help protect cell wall β-glucans from detection or may alternatively produce more α-mannans. The upregulation of cell wall components at low pH suggests similar regulation could be adaptive for growing in a human host; increased chitin could help cells avoid detection by the host immune system. Alternatively, immune detection may be compromised, and the higher capacity of *W. dermatitidis* to evade stress may enable cells to survive. The ability of *W. dermatitidis* to cause severe infections in immunocompetent hosts suggests it is able to overcome host defenses.

## Supplementary Material

Supporting Information
