# Supplementary material for: Comparative Genomic and Transcriptomic Analysis of Wangiella dermatitidis, A Major Cause of Phaeohyphomycosis and a Model Black Yeast Human Pathogen
Source: G3 (Bethesda). 2014 Feb 4;4(4):561–78. doi: 10.1534/g3.113.009241 (PMC4059230; doi:10.1534/g3.113.009241)
Supplement: Supporting Information [file supp_4_4_561__index.html]

Comparative Genomic and Transcriptomic Analysis of Wangiella dermatitidis, A Major Cause of Phaeohyphomycosis and a Model Black Yeast Human Pathogen — Supporting Information 

# Comparative Genomic and Transcriptomic Analysis of *Wangiella dermatitidis*, A Major Cause of Phaeohyphomycosis and a Model Black Yeast Human Pathogen

## Supporting Information for Chen *et al.*, 2014

**Files in this Data Supplement:**

- Supporting Information - Figures S1-S6 and Tables S1-S4 (PDF, 2 MB)
- Figure S1 - Independent expansion of MFS and APC transporter families in *W. dermatitidis* and selected aspergilli. (PDF, 540 KB)
- Figure S2 - Gliotoxin biosynthetic gene cluster in *A. fumigatus* and conservation in *W. dermatitidis*. (PDF, 438 KB)
- Figure S3 - GT2 and chitin synthase tree. (PDF, 719 KB)
- Figure S4 - Classification of nucleotide sugar dehydrogenases. (PDF, 636 KB)
- Figure S5 - Phylogenies of UDP-glucose 6-dehydrogenases (A) and glycosyl transferase family 1 (B). (PDF, 516 KB)
- Figure S6 - Enrichment of cell wall biosynthesis genes and stress response pathway genes under low pH and radiation stress. (PDF, 1 MB)
- Table S1 - Protein domain enrichment and depletion in *W. dermatitidis* compared to other fungi. (PDF, 561 KB)
- Table S2 - Cell wall genes in *W. dermatitidis* and other fungal genomes. (PDF, 645 KB)
- Table S3 - Pathways involved in cell wall stress response and pH signaling. (PDF, 407 KB)
- Table S4 - GSEA tests on gene sets defined by InterPro domain, KEGG pathway, COG, MFS and APC transporter, and cell wall and related pathway. (PDF, 421 KB)
