# Supplementary material for: Comparative Genomic and Transcriptomic Analysis of Wangiella dermatitidis, A Major Cause of Phaeohyphomycosis and a Model Black Yeast Human Pathogen
Source: G3 (Bethesda). 2014 Feb 4;4(4):561–78. doi: 10.1534/g3.113.009241 (PMC4059230; doi:10.1534/g3.113.009241)
Supplement: Supporting Information [file supp_g3.113.009241_009241SI.pdf]

G3: Genes | Genomes | Genetics

Comparative genomic and transcriptomic analysis of *Wangiella dermatitidis*, a major cause of phaeohyphomycosis and a model black yeast human pathogen

Zehua Chen[*], Diego A. Martinez[*], Sharvari Gujja[*], Sean M. Sykes[*], Qiandong Zeng[*], Paul J. Szaniszlo[§], Zheng Wang[†,1], Christina A. Cuomo[*,1]

[*]Broad Institute of MIT and Harvard, Cambridge, MA 02142. [§]The Department of Molecular Biosciences, The University of Texas at Austin, Austin, TX 78712. [†]Center for Bio/Molecular Science and Engineering, Naval Research Laboratory, Washington, D.C. 20375.

[1]**Corresponding authors:** cuomo@broadinstitute.org and zheng.wang@nrl.navy.mil

Data availability: The assembly and annotation of *Wangiella dermatitidis* are available at the NCBI nucleotide database under the accession number AFPA01000000; RNA-Seq differential expression analysis of pH stress is available at the NCBI GEO database under record GSE51646.

DOI: 10.1534/g3.113.009241

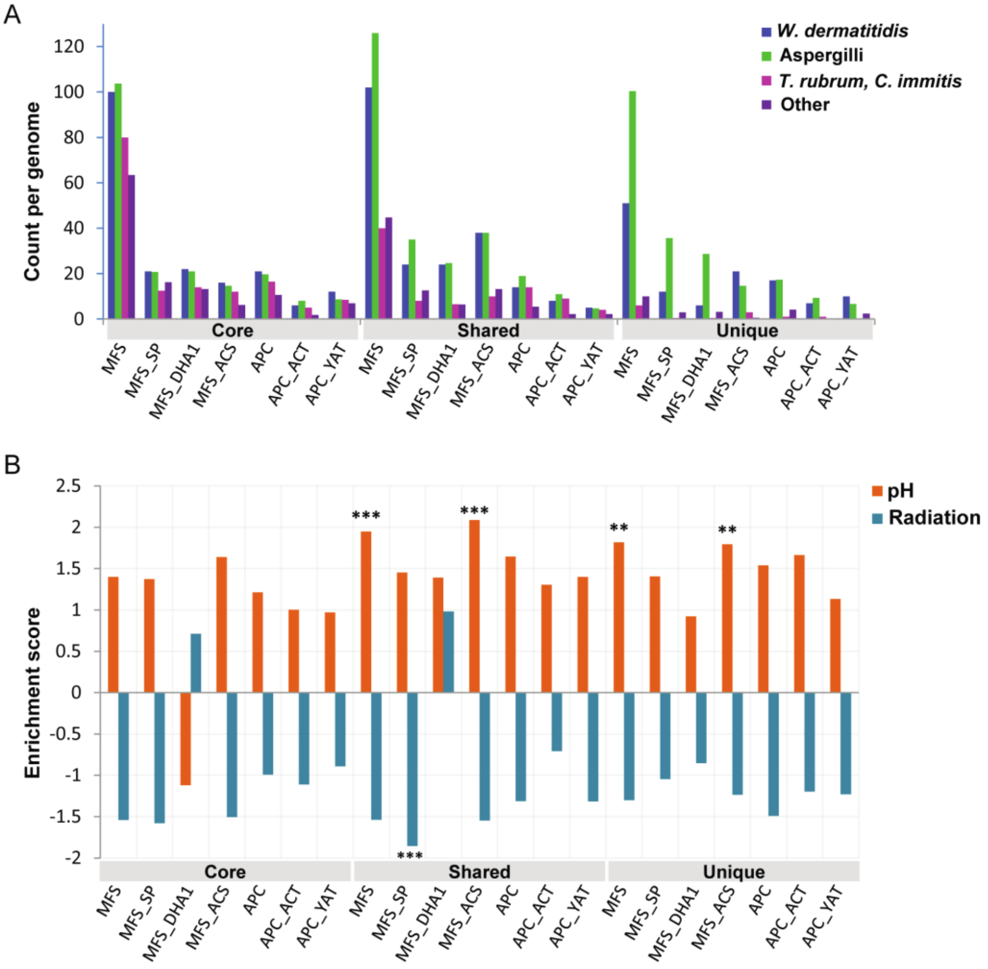

Figure S1 Independent expansion of MFS and APC transporter families in *W. dermatitidis* and selected aspergilli. (**A**) Average number of genes per genome for different category of MFS and APC families (Core families are the ortholog clusters shared by all four fungal groups; Shared, present in at least two out of the four fungal groups; Unique, unique to each group, including species-specific paralogous clusters and unclustered genes). (**B**) Enrichment of different category of MFS and APC transporters under different stress conditions (low pH or radiation). A positive normalized enrichment score (NES) indicates enrichment under stress conditions (pH 2.5 or with radiation), and a negative score indicates enrichment under normal conditions (pH 6 or no radiation). Significant enrichments noted with **: q-value < 0.05; ***: q-value < 0.01.

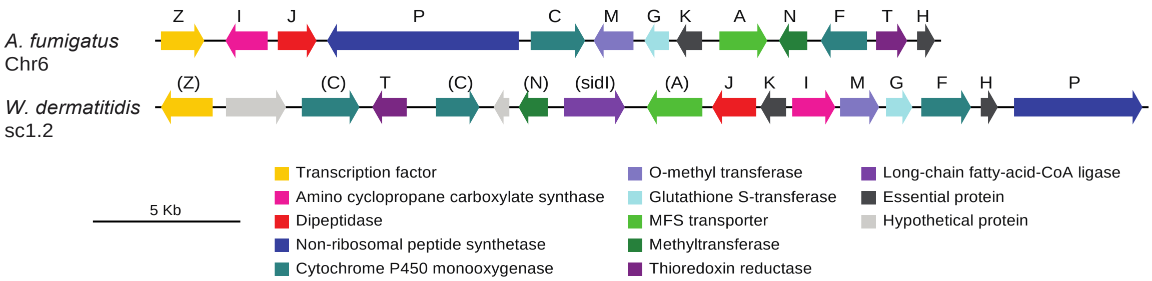

Figure S2 Gliotoxin biosynthetic gene cluster in *A. fumigatus* and conservation in *W. dermatitidis*. Gene symbols above each gene indicate the corresponding orthologs; symbols in parentheses were not identified as single copy orthologs of the corresponding *A. fumigatus* genes, but are part of the gene cluster and share sequence similarity. Gene identifiers for *W. dermatitidis* range from HMPREF1120_02977 (GliZ) to HMPREF1120_02933 (GliP).

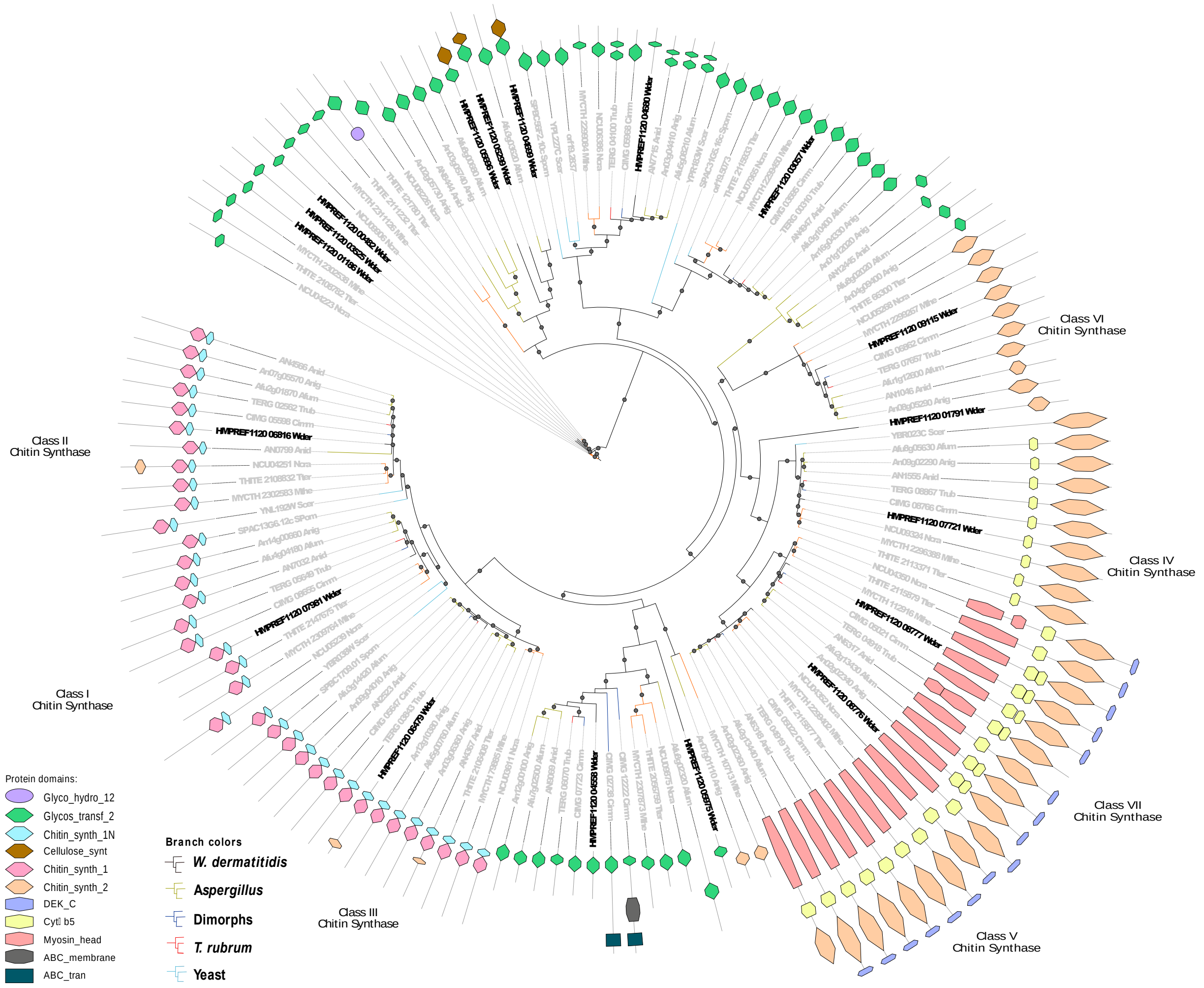

Figure S3 GT2 and chitin synthase tree. Phylogenetic tree of chitin synthase genes and other genes with the GT2 domain. Branch colors indicate species, where black denotes *W. dermatitidis* (bold text labels), blue *C. immitis*, yellow aspergilli, orange Pezizomycotina (*N. crassa*, *M. thermophilia*, *T. terrestris*) and red *T. rubrum*. The phylogeny was estimated with RAxML with the PROTGAMMAWAGF model and 1,000 bootstrap replicates. Nodes with a dot are supported by at least 75% of bootstrap replicates.

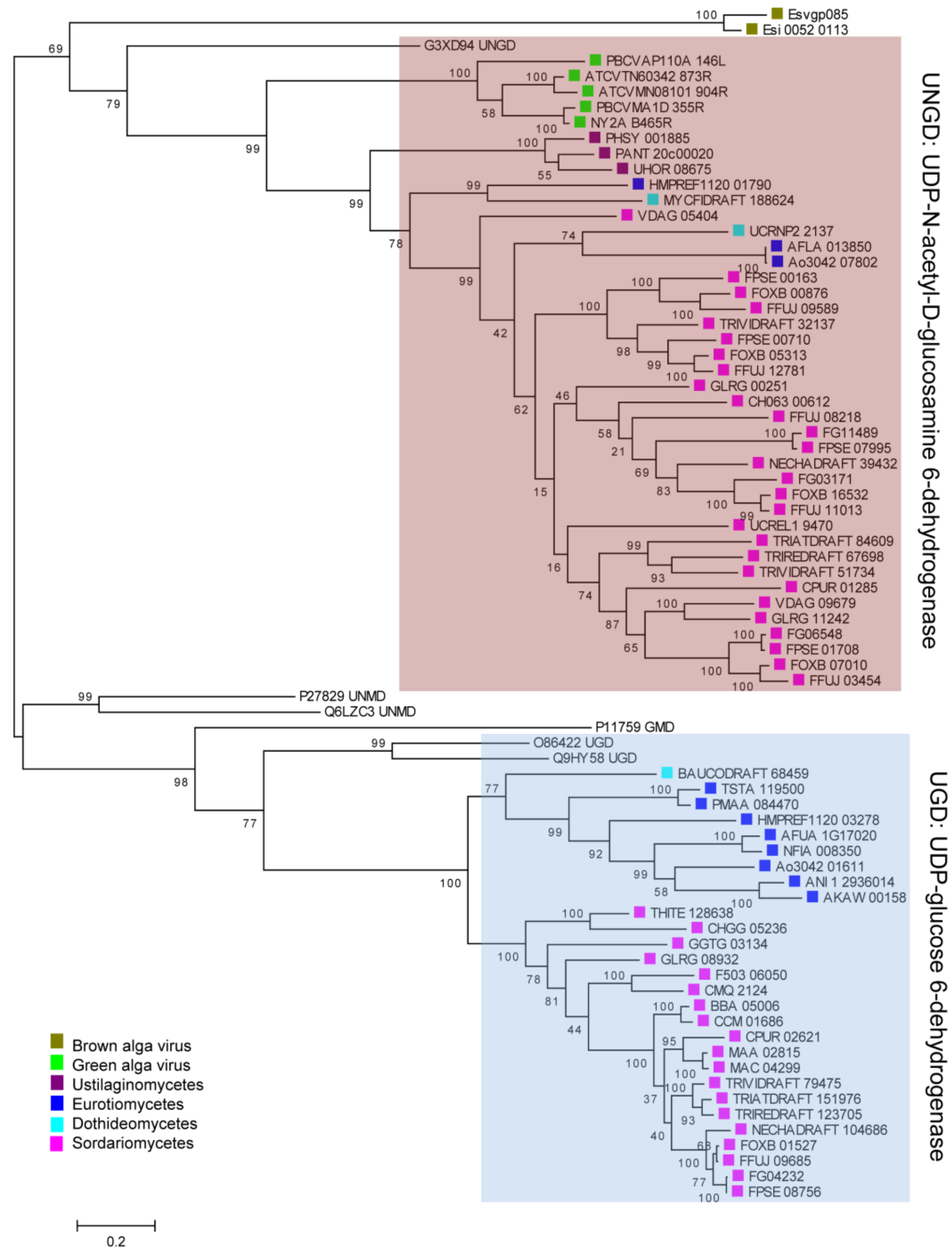

Figure S4 Classification of nucleotide sugar dehydrogenases. A phylogeny was inferred using maximum likelihood with MEGA5, performing 1,000 bootstrap replicates, for UDP-N-acetylglucosamine 6-dehydrogenases (UNGD), UDP-glucose 6-dehydrogenases (UGD), and several experimentally characterized bacterial nucleotide sugar dehydrogenases.

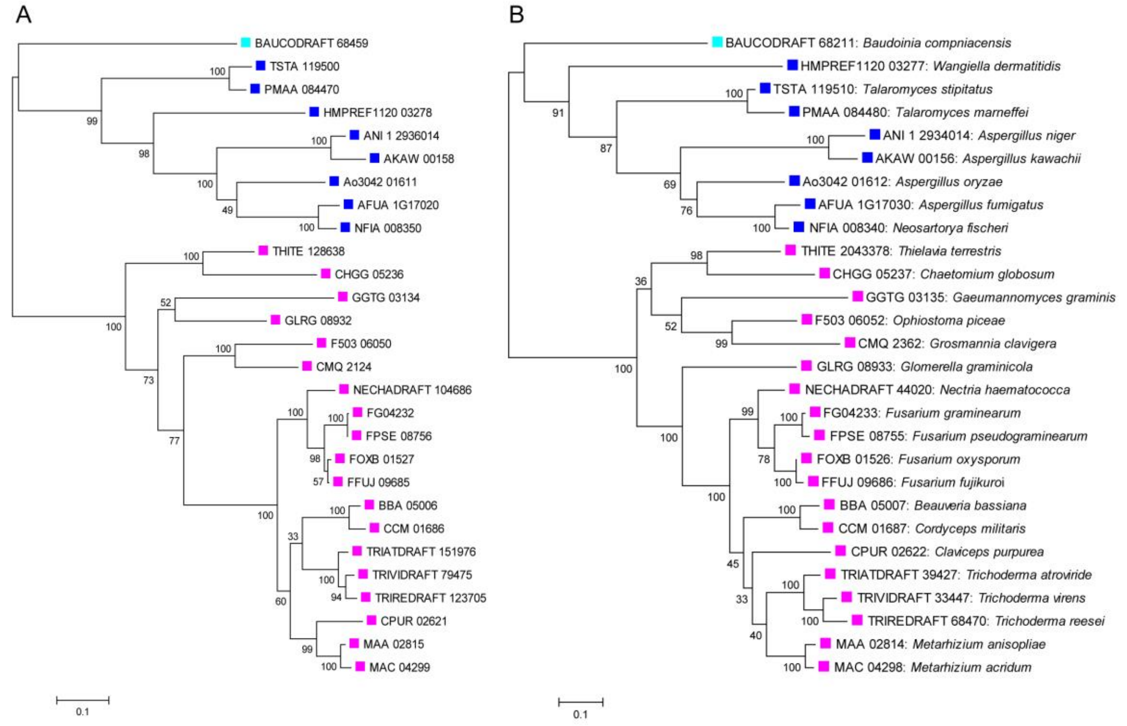

Figure S5 Phylogenies of UDP-glucose 6-dehydrogenases (**A**) and glycosyl transferase family 1 (**B**). The trees were inferred using maximum likelihood with MEGA5, performing 1,000 bootstrap replicates. Pairs of genes from each panel (**A, B**) are adjacent each fungal genome. The colored box icons indicate species groups as follows: Cyan: Dothideomycetes (Doth); Blue: Eurotiomycetes (Euro); Pink: Sordariomycetes (Sord).

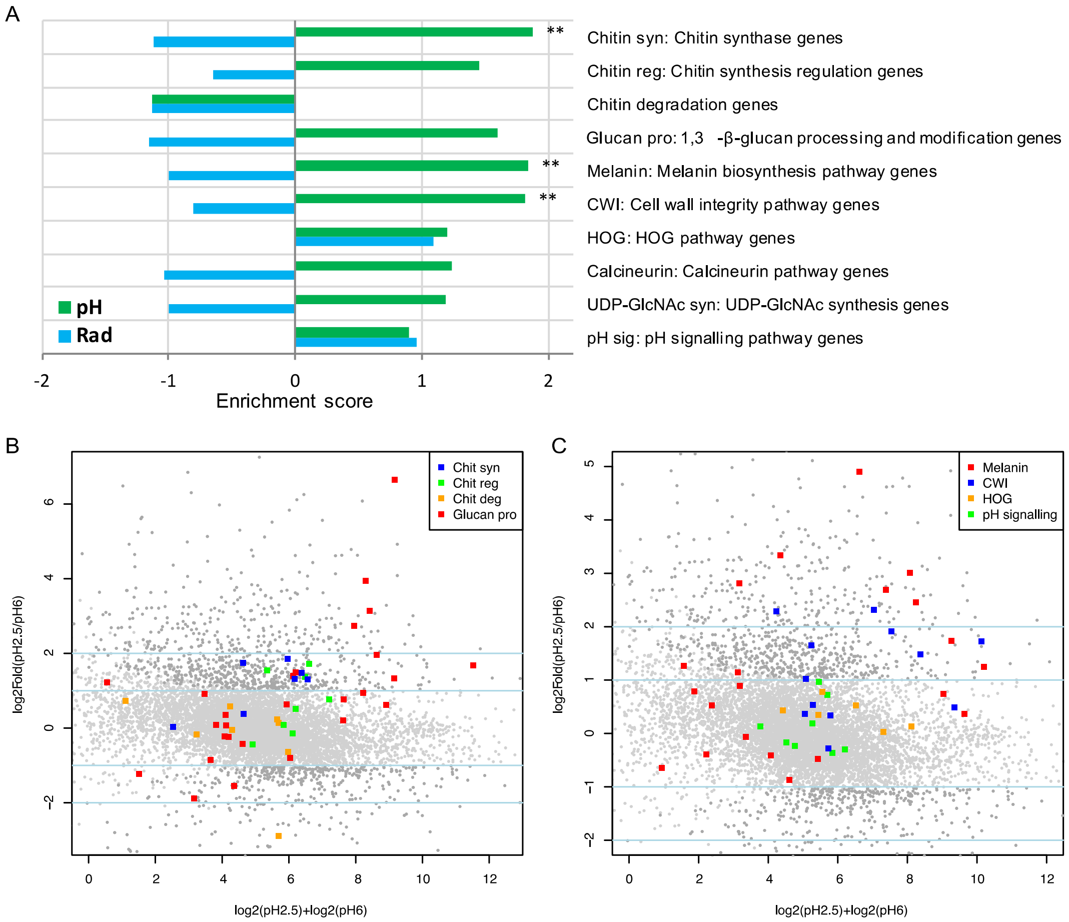

Figure S6 Enrichment of cell wall biosynthesis genes and stress response pathway genes under low pH and radiation stress. (**A**) GSEA enrichment score of different categories of genes or pathways. ** indicates significant enrichment with q-value < 0.05. (**B**) Distribution of different categories of cell wall biosynthesis genes on a MA plot, with light grey and dark grey dots representing the full gene set (dark grey indicates q-value < 1e-10). (**C**) Distribution of different stress response pathway genes on a MA plot.

Table S1 Protein domain enrichment and depletion in *W. dermatitidis* compared to other fungi.

Interpro Domain	Interpro Domain Counts per Genome											Enrichment and depletion in *W. dermatitidis* vs. all others				Enrichment and depletion in *W. dermatitidis* vs. *C. immitis*				Enrichment and depletion in *W. dermatitidis* and T. rubrum vs. all others			
	W. dermatitidis	*A. nidulans*	*A. niger*	*A. fumigatus*	*C. immitis*	*T. rubrum*	*M. thermophila*	*T. terrestris*	*N. crassa*	*S. cerevisiae*	*S. pombe*	Enrich pvalue	Enrich qvalue	Deplete pvalue	Deplete qvalue	Enrich pvalue	Enrich qvalue	Deplete pvalue	Deplete qvalue	Enrich pvalue	Enrich qvalue	Deplete pvalue	Deplete qvalue
**IPR domains enriched or depleted (pvalue < 0.05)**																							
IPR011701::Major facilitator superfamily MFS-1	230	261	318	211	109	128	120	166	114	43	51	2.1E-08	4.8E-05	1.0E+00	1.0E+00	6.5E-09	1.3E-05	1.0E+00	1.0E+00	2.1E-04	1.4E-01	1.0E+00	1.0E+00
IPR020635::Tyrosine-protein kinase, catalytic domain	99	71	17	57	87	92	96	98	34	41	3	6.1E-06	2.4E-03	1.0E+00	1.0E+00	4.6E-01	1.0E+00	6.6E-01	1.0E+00	5.5E-12	1.5E-08	1.0E+00	1.0E+00
IPR020843::Polyketide synthase, enoylreductase	46	49	32	25	25	24	29	36	6	6	1	5.2E-05	1.3E-02	1.0E+00	1.0E+00	2.5E-02	1.0E+00	9.9E-01	1.0E+00	3.8E-04	2.1E-01	1.0E+00	1.0E+00
IPR002290::Serine/threonine-protein kinase domain	117	95	70	87	144	136	124	138	76	87	73	1.6E-01	3.1E-01	8.8E-01	1.0E+00	9.9E-01	1.0E+00	1.0E-02	1.0E+00	5.0E-05	4.4E-02	1.0E+00	1.0E+00
IPR002575::Aminoglycoside phosphotransferase	22	7	29	15	62	51	26	42	3	1	0	6.2E-01	3.1E-01	5.6E-01	1.0E+00	1.0E+00	1.0E+00	2.4E-06	7.6E-03	2.5E-06	3.4E-03	1.0E+00	1.0E+00
IPR010730::Heterokaryon incompatibility	10	12	32	7	2	3	13	28	62	0	0	9.4E-01	3.6E-01	1.8E-01	1.0E+00	2.9E-02	1.0E+00	1.0E+00	1.0E+00	1.0E+00	4.3E-01	1.3E-03	5.0E-01
IPR017853::Glycoside hydrolase, catalytic core	65	136	121	126	37	43	84	93	82	26	28	9.4E-01	3.6E-01	1.0E-01	1.0E+00	1.5E-02	1.0E+00	1.0E+00	1.0E+00	1.0E+00	4.3E-01	4.4E-04	2.1E-01
IPR000992::Stress-induced protein SRP1/TIP1	0	0	0	0	0	0	0	0	0	32	0	9.5E-01	3.6E-01	4.7E-02	1.0E+00	1.0E+00	1.0E+00	1.0E+00	1.0E+00	1.0E+00	4.3E-01	2.5E-03	8.0E-01
IPR005197::Glycoside hydrolase, family 71	0	5	7	8	1	0	3	1	6	0	2	9.5E-01	3.6E-01	4.7E-02	1.0E+00	5.3E-01	1.0E+00	4.7E-01	1.0E+00	1.0E+00	4.3E-01	2.5E-03	8.0E-01
IPR013781::Glycoside hydrolase, subgroup, catalytic core	45	93	82	92	29	34	61	68	62	25	22	9.5E-01	3.6E-01	8.1E-02	1.0E+00	9.5E-02	1.0E+00	9.6E-01	1.0E+00	1.0E+00	4.3E-01	2.6E-03	8.0E-01
IPR006626::Parallel beta-helix repeat	0	9	12	11	0	0	1	2	3	1	0	9.7E-01	3.7E-01	2.7E-02	1.0E+00	1.0E+00	1.0E+00	1.0E+00	1.0E+00	1.0E+00	4.3E-01	8.4E-04	3.5E-01
IPR004906::Pogo transposase / Cenp-B / PDC2, subgroup, DNA-binding HTH domain	0	4	1	19	2	0	2	2	3	1	3	9.8E-01	3.7E-01	2.3E-02	1.0E+00	7.7E-01	1.0E+00	2.3E-01	1.0E+00	1.0E+00	4.3E-01	5.8E-04	2.6E-01
IPR007889::Helix-turn-helix, Psq	0	0	6	30	0	0	2	1	0	0	0	9.8E-01	3.7E-01	1.9E-02	1.0E+00	1.0E+00	1.0E+00	1.0E+00	1.0E+00	1.0E+00	4.3E-01	4.1E-04	2.1E-01
IPR000743::Glycoside hydrolase, family 28	0	11	20	12	0	0	2	7	2	1	0	9.9E-01	3.7E-01	6.2E-03	1.0E+00	1.0E+00	1.0E+00	1.0E+00	1.0E+00	1.0E+00	4.3E-01	4.6E-05	3.2E-02
IPR000254::Cellulose-binding domain, fungal	1	6	8	17	0	0	16	25	21	0	1	1.0E+00	3.7E-01	8.0E-03	1.0E+00	5.3E-01	1.0E+00	1.0E+00	1.0E+00	1.0E+00	4.3E-01	6.3E-06	5.6E-03
IPR000477::Reverse transcriptase	0	4	11	24	1	1	21	41	2	5	14	1.0E+00	3.7E-01	6.6E-06	4.1E-02	5.3E-01	1.0E+00	4.7E-01	1.0E+00	1.0E+00	4.3E-01	2.3E-08	1.5E-04
IPR000845::Nucleoside phosphorylase domain	2	23	34	10	3	2	5	1	4	2	2	1.0E+00	3.7E-01	3.7E-02	1.0E+00	8.4E-01	1.0E+00	7.8E-01	1.0E+00	1.0E+00	4.3E-01	1.5E-03	5.5E-01
IPR001584::Integrase, catalytic core	0	0	16	4	0	0	3	4	0	44	11	1.0E+00	3.7E-01	3.5E-04	7.6E-01	1.0E+00	1.0E+00	1.0E+00	1.0E+00	1.0E+00	4.3E-01	1.6E-07	4.3E-04
IPR004875::DDE superfamily endonuclease, CENP-B-like	0	3	14	49	2	1	1	0	0	1	3	1.0E+00	3.7E-01	5.1E-04	7.6E-01	7.7E-01	1.0E+00	2.3E-01	1.0E+00	1.0E+00	4.3E-01	5.1E-05	3.2E-02
IPR005103::Glycoside hydrolase, family 61	1	10	7	7	0	0	22	18	14	0	0	1.0E+00	3.7E-01	2.5E-02	1.0E+00	5.3E-01	1.0E+00	1.0E+00	1.0E+00	1.0E+00	4.3E-01	8.1E-05	4.6E-02
IPR006600::Pogo transposase / Cenp-B / PDC2, DNA-binding HTH domain	0	4	6	42	2	0	2	2	3	1	3	1.0E+00	3.7E-01	1.2E-03	1.0E+00	7.7E-01	1.0E+00	2.3E-01	1.0E+00	1.0E+00	4.3E-01	1.7E-06	1.8E-03
IPR011050::Pectin lyase fold/virulence factor	3	36	32	34	1	1	16	16	12	2	0	1.0E+00	3.7E-01	1.9E-03	1.0E+00	3.5E-01	1.0E+00	1.0E+00	1.0E+00	1.0E+00	4.3E-01	2.1E-07	4.3E-04
IPR012334::Pectin lyase fold	3	35	32	34	1	1	15	17	12	1	0	1.0E+00	3.7E-01	2.1E-03	1.0E+00	3.5E-01	1.0E+00	1.0E+00	1.0E+00	1.0E+00	4.3E-01	2.8E-07	4.4E-04
IPR013103::Reverse transcriptase, RNA-dependent DNA polymerase	0	1	16	0	0	0	1	2	0	42	0	1.0E+00	3.7E-01	2.9E-03	1.0E+00	1.0E+00	1.0E+00	1.0E+00	1.0E+00	1.0E+00	4.3E-01	1.1E-05	8.3E-03
IPR015820::Retrotransposon Ty1 A, N-terminal	0	0	0	0	0	0	0	0	0	80	0	1.0E+00	3.7E-01	6.1E-04	7.6E-01	1.0E+00	1.0E+00	1.0E+00	1.0E+00	1.0E+00	4.3E-01	4.9E-07	6.1E-04
**Glycoside hydrolase family**																							
IPR000933::Glycoside hydrolase, family 29	0	0	1	0	0	0	0	0	0	0	0	8.8E-02	3.1E-01	9.1E-01	1.0E+00	1.0E+00	1.0E+00	1.0E+00	1.0E+00	1.7E-01	3.6E-01	8.3E-01	1.0E+00
IPR016286::Glycoside hydrolase, family 29, bacteria/metazoa/fungi	0	0	1	0	0	0	0	0	0	0	0	8.8E-02	3.1E-01	9.1E-01	1.0E+00	1.0E+00	1.0E+00	1.0E+00	1.0E+00	1.7E-01	3.6E-01	8.3E-01	1.0E+00
IPR013529::Glycoside hydrolase, family 42, N-terminal	1	0	0	1	0	0	0	0	0	0	0	1.7E-01	3.1E-01	1.0E+00	1.0E+00	5.3E-01	1.0E+00	1.0E+00	1.0E+00	3.0E-01	3.6E-01	1.0E+00	1.0E+00
IPR016282::Glycoside hydrolase, family 5, endoglucanase B	0	1	0	1	0	0	0	0	0	0	0	1.7E-01	3.1E-01	8.3E-01	1.0E+00	1.0E+00	1.0E+00	1.0E+00	1.0E+00	3.0E-01	3.6E-01	7.0E-01	1.0E+00
IPR018087::Glycoside hydrolase, family 5, conserved site	3	0	1	2	1	0	0	3	0	4	1	2.3E-01	3.1E-01	9.8E-01	1.0E+00	3.5E-01	1.0E+00	1.0E+00	1.0E+00	6.3E-01	3.6E-01	8.0E-01	1.0E+00
IPR000757::Glycoside hydrolase, family 16	12	12	10	11	6	7	14	13	15	3	1	2.4E-01	3.1E-01	9.1E-01	1.0E+00	1.7E-01	1.0E+00	9.7E-01	1.0E+00	4.1E-01	3.6E-01	7.7E-01	1.0E+00
IPR005089::Glycoside hydrolase, family 18, carbohydrate-binding	0	0	0	1	0	0	1	0	0	1	0	2.4E-01	3.1E-01	7.6E-01	1.0E+00	1.0E+00	1.0E+00	1.0E+00	1.0E+00	4.2E-01	3.6E-01	5.8E-01	1.0E+00
IPR006215::Glycoside hydrolase, melibiase	1	0	0	1	0	0	0	0	0	0	1	2.4E-01	3.1E-01	1.0E+00	1.0E+00	5.3E-01	1.0E+00	1.0E+00	1.0E+00	4.2E-01	3.6E-01	1.0E+00	1.0E+00
IPR011330::Glycoside hydrolase/deacetylase, beta/alpha-barrel	9	11	10	9	5	6	7	7	7	3	2	2.5E-01	3.1E-01	9.2E-01	1.0E+00	2.7E-01	1.0E+00	9.6E-01	1.0E+00	3.1E-01	3.6E-01	8.6E-01	1.0E+00
IPR000490::Glycoside hydrolase, family 17	4	1	3	2	3	2	3	2	3	4	1	3.1E-01	3.1E-01	9.4E-01	1.0E+00	5.6E-01	1.0E+00	9.2E-01	1.0E+00	4.4E-01	3.6E-01	8.5E-01	1.0E+00
IPR005194::Glycoside hydrolase, family 65, C-terminal	0	1	0	1	1	0	0	0	0	1	0	3.1E-01	3.1E-01	6.9E-01	1.0E+00	5.3E-01	1.0E+00	4.7E-01	1.0E+00	5.2E-01	3.6E-01	4.8E-01	1.0E+00
IPR000726::Glycoside hydrolase, family 19, catalytic	0	0	0	0	2	1	1	1	0	0	0	3.7E-01	3.1E-01	6.3E-01	1.0E+00	7.7E-01	1.0E+00	2.3E-01	1.0E+00	6.0E-01	3.6E-01	9.6E-01	1.0E+00
IPR000805::Glycoside hydrolase, family 26	0	3	1	0	0	0	1	0	0	0	0	3.7E-01	3.1E-01	6.3E-01	1.0E+00	1.0E+00	1.0E+00	1.0E+00	1.0E+00	6.0E-01	3.6E-01	4.0E-01	1.0E+00
IPR002196::Glycoside hydrolase, family 24	0	1	0	0	0	2	1	1	0	0	0	3.7E-01	3.1E-01	6.3E-01	1.0E+00	1.0E+00	1.0E+00	1.0E+00	1.0E+00	2.0E-01	3.6E-01	1.0E+00	1.0E+00
IPR008270::Glycoside hydrolase, family 25, active site	0	2	0	0	0	0	1	1	1	0	0	3.7E-01	3.1E-01	6.3E-01	1.0E+00	1.0E+00	1.0E+00	1.0E+00	1.0E+00	6.0E-01	3.6E-01	4.0E-01	1.0E+00
IPR001360::Glycoside hydrolase, family 1	2	3	3	5	0	0	1	1	1	0	0	4.2E-01	3.1E-01	9.5E-01	1.0E+00	2.8E-01	1.0E+00	1.0E+00	1.0E+00	7.7E-01	3.7E-01	7.3E-01	1.0E+00
IPR000334::Glycoside hydrolase, family 45	0	1	0	1	0	0	1	2	1	0	0	4.3E-01	3.1E-01	5.7E-01	1.0E+00	1.0E+00	1.0E+00	1.0E+00	1.0E+00	6.6E-01	3.6E-01	3.4E-01	1.0E+00
IPR005154::Glycoside hydrolase, family 67, alpha glucuronidase, N-terminal	1	1	1	1	0	0	1	0	1	0	0	4.3E-01	3.1E-01	9.9E-01	1.0E+00	5.3E-01	1.0E+00	1.0E+00	1.0E+00	6.6E-01	3.6E-01	9.4E-01	1.0E+00
IPR011099::Glycosyl hydrolase 67, C-terminal	1	1	1	1	0	0	1	0	1	0	0	4.3E-01	3.1E-01	9.9E-01	1.0E+00	5.3E-01	1.0E+00	1.0E+00	1.0E+00	6.6E-01	3.6E-01	9.4E-01	1.0E+00
IPR011100::Glycosyl hydrolase 67 middle	1	1	1	1	0	0	1	0	1	0	0	4.3E-01	3.1E-01	9.9E-01	1.0E+00	5.3E-01	1.0E+00	1.0E+00	1.0E+00	6.6E-01	3.6E-01	9.4E-01	1.0E+00
IPR011395::Glycoside hydrolase, family 67, alpha glucuronidase	1	1	1	1	0	0	1	0	1	0	0	4.3E-01	3.1E-01	9.9E-01	1.0E+00	5.3E-01	1.0E+00	1.0E+00	1.0E+00	6.6E-01	3.6E-01	9.4E-01	1.0E+00
IPR022790::Endoglucanase H/Glycosyl hydrolase family 26	0	3	1	0	0	0	1	0	1	0	0	4.3E-01	3.1E-01	5.7E-01	1.0E+00	1.0E+00	1.0E+00	1.0E+00	1.0E+00	6.6E-01	3.6E-01	3.4E-01	1.0E+00
IPR000322::Glycoside hydrolase, family 31	6	10	7	7	2	2	6	8	5	1	4	4.5E-01	3.1E-01	8.3E-01	1.0E+00	1.8E-01	1.0E+00	9.9E-01	1.0E+00	8.2E-01	3.8E-01	4.3E-01	1.0E+00
IPR002594::Glycoside hydrolase, family 12	2	1	4	4	0	0	1	3	2	0	0	4.5E-01	3.1E-01	9.4E-01	1.0E+00	2.8E-01	1.0E+00	1.0E+00	1.0E+00	8.0E-01	3.8E-01	6.9E-01	1.0E+00
IPR006589::Glycosyl hydrolase, family 13, subfamily, catalytic domain	8	11	10	10	4	2	7	4	9	7	8	4.5E-01	3.1E-01	8.0E-01	1.0E+00	2.5E-01	1.0E+00	9.7E-01	1.0E+00	9.0E-01	4.1E-01	2.6E-01	1.0E+00
IPR001540::Glycoside hydrolase, family 20	2	2	3	2	3	2	1	1	1	0	0	4.8E-01	3.1E-01	9.3E-01	1.0E+00	8.4E-01	1.0E+00	7.8E-01	1.0E+00	3.5E-01	3.6E-01	9.4E-01	1.0E+00
IPR002252::Glycoside hydrolase, family 36	1	3	1	2	0	0	0	0	0	0	0	4.8E-01	3.1E-01	9.8E-01	1.0E+00	5.3E-01	1.0E+00	1.0E+00	1.0E+00	7.2E-01	3.6E-01	9.1E-01	1.0E+00
IPR006046::Glycoside hydrolase, family 13	0	3	2	1	0	0	0	1	0	0	0	4.8E-01	3.1E-01	5.2E-01	1.0E+00	1.0E+00	1.0E+00	1.0E+00	1.0E+00	7.2E-01	3.6E-01	2.8E-01	1.0E+00
IPR011683::Glycosyl hydrolase 53	0	1	2	1	0	0	1	1	1	0	0	4.8E-01	3.1E-01	5.2E-01	1.0E+00	1.0E+00	1.0E+00	1.0E+00	1.0E+00	7.2E-01	3.6E-01	2.8E-01	1.0E+00
IPR015883::Glycoside hydrolase, family 20, catalytic core	2	2	3	2	3	2	1	1	1	0	0	4.8E-01	3.1E-01	9.3E-01	1.0E+00	8.4E-01	1.0E+00	7.8E-01	1.0E+00	3.5E-01	3.6E-01	9.4E-01	1.0E+00
IPR018053::Glycoside hydrolase, family 32, active site	0	1	1	2	0	0	0	0	0	1	2	4.8E-01	3.1E-01	5.2E-01	1.0E+00	1.0E+00	1.0E+00	1.0E+00	1.0E+00	7.2E-01	3.6E-01	2.8E-01	1.0E+00
IPR019801::Glycoside hydrolase, family 35, conserved site	1	0	2	2	0	0	0	1	1	0	0	4.8E-01	3.1E-01	9.8E-01	1.0E+00	5.3E-01	1.0E+00	1.0E+00	1.0E+00	7.2E-01	3.6E-01	9.1E-01	1.0E+00
IPR004199::Glycoside hydrolase, family 42, domain 5	1	2	0	1	0	0	1	1	2	0	0	5.2E-01	3.1E-01	9.7E-01	1.0E+00	5.3E-01	1.0E+00	1.0E+00	1.0E+00	7.7E-01	3.7E-01	8.7E-01	1.0E+00
IPR005195::Glycoside hydrolase, family 65, central catalytic	1	1	1	1	1	1	0	0	0	1	0	5.2E-01	3.1E-01	9.7E-01	1.0E+00	7.7E-01	1.0E+00	1.0E+00	1.0E+00	3.9E-01	3.6E-01	9.7E-01	1.0E+00
IPR005196::Glycoside hydrolase, family 65, N-terminal	1	1	1	1	1	1	0	0	0	1	0	5.2E-01	3.1E-01	9.7E-01	1.0E+00	7.7E-01	1.0E+00	1.0E+00	1.0E+00	3.9E-01	3.6E-01	9.7E-01	1.0E+00
IPR014718::Glycoside hydrolase-type carbohydrate-binding, subgroup	4	5	3	4	3	3	5	4	6	4	1	5.5E-01	3.1E-01	8.1E-01	1.0E+00	5.6E-01	1.0E+00	9.2E-01	1.0E+00	6.1E-01	3.6E-01	7.0E-01	1.0E+00
IPR001139::Glycoside hydrolase, family 30	1	0	1	1	0	0	2	3	1	0	0	5.6E-01	3.1E-01	9.6E-01	1.0E+00	5.3E-01	1.0E+00	1.0E+00	1.0E+00	8.0E-01	3.8E-01	8.2E-01	1.0E+00
IPR004888::Glycoside hydrolase, family 63	2	2	2	1	1	1	2	3	3	1	1	5.6E-01	3.1E-01	8.9E-01	1.0E+00	5.4E-01	1.0E+00	1.0E+00	1.0E+00	7.0E-01	3.6E-01	7.4E-01	1.0E+00
IPR005193::Glycoside hydrolase, family 62, arabinosidase	0	2	1	2	0	0	2	2	0	0	0	5.6E-01	3.1E-01	4.4E-01	1.0E+00	1.0E+00	1.0E+00	1.0E+00	1.0E+00	8.0E-01	3.8E-01	2.0E-01	1.0E+00
IPR002053::Glycoside hydrolase, family 25	0	3	0	3	0	1	1	1	1	0	0	6.0E-01	3.1E-01	4.0E-01	1.0E+00	1.0E+00	1.0E+00	1.0E+00	1.0E+00	8.4E-01	3.9E-01	7.8E-01	1.0E+00
IPR018077::Glycoside hydrolase, family 25 subgroup	0	3	0	3	0	1	1	1	1	0	0	6.0E-01	3.1E-01	4.0E-01	1.0E+00	1.0E+00	1.0E+00	1.0E+00	1.0E+00	8.4E-01	3.9E-01	7.8E-01	1.0E+00
IPR018120::Glycoside hydrolase, family 1, active site	1	1	2	3	0	0	1	1	1	0	0	6.0E-01	3.1E-01	9.5E-01	1.0E+00	5.3E-01	1.0E+00	1.0E+00	1.0E+00	8.4E-01	3.9E-01	7.8E-01	1.0E+00
IPR011013::Glycoside hydrolase-type carbohydrate-binding	11	20	14	15	6	7	13	14	13	6	6	6.1E-01	3.1E-01	6.3E-01	1.0E+00	2.2E-01	1.0E+00	9.6E-01	1.0E+00	8.4E-01	3.9E-01	3.0E-01	1.0E+00
IPR000602::Glycoside hydrolase, family 38, core	1	1	1	1	1	1	1	1	1	1	1	6.7E-01	3.1E-01	9.2E-01	1.0E+00	7.7E-01	1.0E+00	1.0E+00	1.0E+00	6.2E-01	3.6E-01	8.8E-01	1.0E+00
IPR004193::Glycoside hydrolase, family 13, N-terminal	1	1	1	1	1	1	1	1	2	1	0	6.7E-01	3.1E-01	9.2E-01	1.0E+00	7.7E-01	1.0E+00	1.0E+00	1.0E+00	6.2E-01	3.6E-01	8.8E-01	1.0E+00
IPR011682::Glycosyl hydrolases 38, C-terminal	1	1	1	1	1	1	1	1	1	1	1	6.7E-01	3.1E-01	9.2E-01	1.0E+00	7.7E-01	1.0E+00	1.0E+00	1.0E+00	6.2E-01	3.6E-01	8.8E-01	1.0E+00
IPR015341::Glycoside hydrolase, family 38, central domain	1	1	1	1	1	1	1	1	1	1	1	6.7E-01	3.1E-01	9.2E-01	1.0E+00	7.7E-01	1.0E+00	1.0E+00	1.0E+00	6.2E-01	3.6E-01	8.8E-01	1.0E+00
IPR016840::Glycoside hydrolase, family 43, endo-1, 5-alpha-L-arabinosidase	0	4	3	2	1	0	1	1	0	0	0	6.7E-01	3.1E-01	3.3E-01	1.0E+00	5.3E-01	1.0E+00	4.7E-01	1.0E+00	8.9E-01	4.0E-01	1.1E-01	1.0E+00
IPR000111::Glycoside hydrolase, clan GH-D	2	6	7	7	0	0	2	1	0	0	1	6.8E-01	3.1E-01	8.1E-01	1.0E+00	2.8E-01	1.0E+00	1.0E+00	1.0E+00	9.5E-01	4.2E-01	3.5E-01	1.0E+00
IPR001524::Glycoside hydrolase, family 6, conserved site	0	2	2	1	0	0	3	2	3	0	0	7.0E-01	3.1E-01	3.0E-01	1.0E+00	1.0E+00	1.0E+00	1.0E+00	1.0E+00	9.1E-01	4.1E-01	9.4E-02	1.0E+00
IPR006047::Glycosyl hydrolase, family 13, catalytic domain	8	12	17	15	4	2	7	4	10	9	12	7.0E-01	3.1E-01	5.7E-01	1.0E+00	2.5E-01	1.0E+00	9.7E-01	1.0E+00	9.8E-01	4.3E-01	6.3E-02	1.0E+00
IPR017168::Glycoside hydrolase, family 16, CRH1, predicted	2	3	3	3	2	0	3	3	3	2	0	7.0E-01	3.1E-01	7.9E-01	1.0E+00	7.2E-01	1.0E+00	9.2E-01	1.0E+00	9.5E-01	4.3E-01	3.2E-01	1.0E+00
IPR005198::Glycoside hydrolase, family 76	6	7	11	8	3	3	9	9	10	2	4	7.1E-01	3.1E-01	6.0E-01	1.0E+00	3.1E-01	1.0E+00	9.7E-01	1.0E+00	9.2E-01	4.2E-01	2.2E-01	1.0E+00
IPR013189::Glycosyl hydrolase family 32, C-terminal	1	2	5	2	0	0	0	1	1	1	1	7.3E-01	3.1E-01	8.8E-01	1.0E+00	5.3E-01	1.0E+00	1.0E+00	1.0E+00	9.2E-01	4.1E-01	5.8E-01	1.0E+00
IPR000165::Glycoside hydrolase, family 15	0	2	1	3	1	1	1	2	1	1	1	7.5E-01	3.1E-01	2.5E-01	1.0E+00	5.3E-01	1.0E+00	4.7E-01	1.0E+00	9.3E-01	4.2E-01	5.3E-01	1.0E+00
IPR005200::Glycoside hydrolase, family 81	1	1	1	1	1	1	1	1	1	2	2	7.5E-01	3.1E-01	8.6E-01	1.0E+00	7.7E-01	1.0E+00	1.0E+00	1.0E+00	7.4E-01	3.6E-01	7.7E-01	1.0E+00
IPR006101::Glycoside hydrolase, family 2	1	4	1	1	0	0	3	3	2	0	0	7.5E-01	3.1E-01	8.6E-01	1.0E+00	5.3E-01	1.0E+00	1.0E+00	1.0E+00	9.3E-01	4.2E-01	5.3E-01	1.0E+00
IPR013780::Glycosyl hydrolase, family 13, all-beta	8	11	18	18	3	2	10	8	10	8	9	7.5E-01	3.1E-01	5.1E-01	1.0E+00	1.5E-01	1.0E+00	9.9E-01	1.0E+00	9.9E-01	4.3E-01	4.2E-02	1.0E+00
IPR001661::Glycoside hydrolase, family 37	1	1	1	1	1	1	2	2	2	2	1	7.7E-01	3.1E-01	8.4E-01	1.0E+00	7.7E-01	1.0E+00	1.0E+00	1.0E+00	7.7E-01	3.7E-01	7.3E-01	1.0E+00
IPR018232::Glycoside hydrolase, family 37, conserved site	1	1	1	1	1	1	2	2	2	2	1	7.7E-01	3.1E-01	8.4E-01	1.0E+00	7.7E-01	1.0E+00	1.0E+00	1.0E+00	7.7E-01	3.7E-01	7.3E-01	1.0E+00
IPR001382::Glycoside hydrolase, family 47	5	8	5	6	7	6	9	10	9	3	2	7.8E-01	3.1E-01	5.3E-01	1.0E+00	8.5E-01	1.0E+00	5.4E-01	1.0E+00	6.9E-01	3.6E-01	5.6E-01	1.0E+00
IPR001362::Glycoside hydrolase, family 32	1	2	5	4	0	0	0	1	1	1	2	7.9E-01	3.2E-01	8.1E-01	1.0E+00	5.3E-01	1.0E+00	1.0E+00	1.0E+00	9.5E-01	4.3E-01	4.5E-01	1.0E+00
IPR002241::Glycoside hydrolase, family 27	1	3	5	5	0	0	2	1	0	0	1	8.1E-01	3.2E-01	7.9E-01	1.0E+00	5.3E-01	1.0E+00	1.0E+00	1.0E+00	9.6E-01	4.3E-01	4.1E-01	1.0E+00
IPR018208::Glycoside hydrolase, family 11, active site	0	2	3	3	0	0	6	2	2	0	0	8.1E-01	3.2E-01	1.9E-01	1.0E+00	1.0E+00	1.0E+00	1.0E+00	1.0E+00	9.6E-01	4.3E-01	3.8E-02	1.0E+00
IPR001944::Glycoside hydrolase, family 35	1	4	5	4	0	0	1	2	2	0	0	8.3E-01	3.2E-01	7.7E-01	1.0E+00	5.3E-01	1.0E+00	1.0E+00	1.0E+00	9.7E-01	4.3E-01	3.7E-01	1.0E+00
IPR002044::Glycoside hydrolase, carbohydrate-binding	0	4	1	4	1	1	2	3	3	0	0	8.3E-01	3.2E-01	1.7E-01	1.0E+00	5.3E-01	1.0E+00	4.7E-01	1.0E+00	9.7E-01	4.3E-01	3.7E-01	1.0E+00
IPR010905::Glycosyl hydrolase, family 88	1	6	3	5	0	0	1	1	2	0	0	8.3E-01	3.2E-01	7.7E-01	1.0E+00	5.3E-01	1.0E+00	1.0E+00	1.0E+00	9.7E-01	4.3E-01	3.7E-01	1.0E+00
IPR013148::Glycosyl hydrolases family 32, N-terminal	1	2	5	4	0	0	0	1	3	1	2	8.3E-01	3.2E-01	7.7E-01	1.0E+00	5.3E-01	1.0E+00	1.0E+00	1.0E+00	9.7E-01	4.3E-01	3.7E-01	1.0E+00
IPR019800::Glycoside hydrolase, family 3, active site	2	7	3	5	2	2	3	4	4	0	1	8.3E-01	3.2E-01	6.3E-01	1.0E+00	7.2E-01	1.0E+00	9.2E-01	1.0E+00	8.6E-01	3.9E-01	4.6E-01	1.0E+00
IPR002772::Glycoside hydrolase, family 3, C-terminal	6	19	15	16	3	5	10	11	8	0	1	8.6E-01	3.3E-01	3.8E-01	1.0E+00	3.1E-01	1.0E+00	9.7E-01	1.0E+00	9.4E-01	4.2E-01	1.7E-01	1.0E+00
IPR001000::Glycoside hydrolase, family 10	1	3	1	4	0	0	4	6	4	0	0	8.8E-01	3.4E-01	6.7E-01	1.0E+00	5.3E-01	1.0E+00	1.0E+00	1.0E+00	9.8E-01	4.3E-01	2.4E-01	1.0E+00
IPR011613::Glycoside hydrolase 15-related	1	2	2	5	1	1	2	3	2	1	2	8.8E-01	3.4E-01	6.7E-01	1.0E+00	7.7E-01	1.0E+00	1.0E+00	1.0E+00	9.1E-01	4.1E-01	4.5E-01	1.0E+00
IPR001137::Glycoside hydrolase, family 11	0	2	4	3	0	0	8	5	2	0	0	8.9E-01	3.4E-01	1.1E-01	1.0E+00	1.0E+00	1.0E+00	1.0E+00	1.0E+00	9.9E-01	4.3E-01	1.3E-02	1.0E+00
IPR001547::Glycoside hydrolase, family 5	5	15	10	13	2	3	9	13	6	4	3	8.9E-01	3.4E-01	3.6E-01	1.0E+00	2.7E-01	1.0E+00	9.9E-01	1.0E+00	9.8E-01	4.3E-01	7.7E-02	1.0E+00
IPR001764::Glycoside hydrolase, family 3, N-terminal	7	21	17	18	5	6	12	14	10	0	1	8.9E-01	3.4E-01	3.1E-01	1.0E+00	4.6E-01	1.0E+00	9.0E-01	1.0E+00	9.5E-01	4.3E-01	1.3E-01	1.0E+00
IPR013319::Glycoside hydrolase, family 11/12, catalytic domain	2	3	8	7	0	0	9	8	4	0	0	8.9E-01	3.4E-01	5.0E-01	1.0E+00	2.8E-01	1.0E+00	1.0E+00	1.0E+00	9.9E-01	4.3E-01	7.3E-02	1.0E+00
IPR001722::Glycoside hydrolase, family 7	0	3	2	4	0	0	6	5	5	0	0	9.0E-01	3.5E-01	9.9E-02	1.0E+00	1.0E+00	1.0E+00	1.0E+00	1.0E+00	9.9E-01	4.3E-01	1.1E-02	1.0E+00
IPR006104::Glycoside hydrolase, family 2, carbohydrate-binding	2	9	5	5	1	2	7	7	5	0	0	9.0E-01	3.5E-01	4.7E-01	1.0E+00	5.4E-01	1.0E+00	1.0E+00	1.0E+00	9.4E-01	4.2E-01	2.6E-01	1.0E+00
IPR006710::Glycoside hydrolase, family 43	4	17	10	17	1	1	11	8	5	0	0	9.0E-01	3.5E-01	3.5E-01	1.0E+00	2.2E-01	1.0E+00	1.0E+00	1.0E+00	1.0E+00	4.3E-01	2.8E-02	1.0E+00
IPR012939::Glycosyl hydrolase 92	0	5	5	5	1	2	2	2	2	0	0	9.0E-01	3.5E-01	9.9E-02	1.0E+00	5.3E-01	1.0E+00	4.7E-01	1.0E+00	9.4E-01	4.2E-01	3.8E-01	1.0E+00
IPR001579::Glycoside hydrolase, chitinase active site	5	16	11	15	7	11	5	12	7	2	0	9.3E-01	3.6E-01	2.5E-01	1.0E+00	8.5E-01	1.0E+00	5.4E-01	1.0E+00	5.4E-01	3.6E-01	6.7E-01	1.0E+00
IPR006102::Glycoside hydrolase, family 2, immunoglobulin-like beta-sandw ich	2	10	6	6	1	2	7	8	5	0	0	9.3E-01	3.6E-01	3.8E-01	1.0E+00	5.4E-01	1.0E+00	1.0E+00	1.0E+00	9.7E-01	4.3E-01	1.7E-01	1.0E+00
IPR006103::Glycoside hydrolase, family 2, TIM barrel	1	7	3	2	1	1	5	6	3	0	0	9.3E-01	3.6E-01	5.2E-01	1.0E+00	7.7E-01	1.0E+00	1.0E+00	1.0E+00	9.7E-01	4.3E-01	2.7E-01	1.0E+00
IPR013812::Glycoside hydrolase, family 2/20, immunoglobulin-like beta-sandw ich domain	2	10	6	6	1	2	7	8	5	0	0	9.3E-01	3.6E-01	3.8E-01	1.0E+00	5.4E-01	1.0E+00	1.0E+00	1.0E+00	9.7E-01	4.3E-01	1.7E-01	1.0E+00
IPR017853::Glycoside hydrolase, catalytic core	65	136	121	126	37	43	84	93	82	26	28	9.4E-01	3.6E-01	1.0E-01	1.0E+00	1.5E-02	1.0E+00	1.0E+00	1.0E+00	1.0E+00	4.3E-01	4.4E-04	2.1E-01
IPR005197::Glycoside hydrolase, family 71	0	5	7	8	1	0	3	1	6	0	2	9.5E-01	3.6E-01	4.7E-02	1.0E+00	5.3E-01	1.0E+00	4.7E-01	1.0E+00	1.0E+00	4.3E-01	2.5E-03	8.0E-01
IPR013781::Glycoside hydrolase, subgroup, catalytic core	45	93	82	92	29	34	61	68	62	25	22	9.5E-01	3.6E-01	8.1E-02	1.0E+00	9.5E-02	1.0E+00	9.6E-01	1.0E+00	1.0E+00	4.3E-01	2.6E-03	8.0E-01
IPR001223::Glycoside hydrolase, family 18, catalytic domain	6	20	14	18	9	14	10	15	12	1	0	9.6E-01	3.7E-01	1.5E-01	1.0E+00	8.9E-01	1.0E+00	4.2E-01	1.0E+00	5.6E-01	3.6E-01	6.3E-01	1.0E+00
IPR000743::Glycoside hydrolase, family 28	0	11	20	12	0	0	2	7	2	1	0	9.9E-01	3.7E-01	6.2E-03	1.0E+00	1.0E+00	1.0E+00	1.0E+00	1.0E+00	1.0E+00	4.3E-01	4.6E-05	3.2E-02
IPR002037::Glycoside hydrolase, family 8	0	0	0	0	0	0	0	0	0	0	0	1.0E+00	3.7E-01	1.0E+00	1.0E+00	1.0E+00	1.0E+00	1.0E+00	1.0E+00	1.0E+00	4.3E-01	1.0E+00	1.0E+00
IPR005103::Glycoside hydrolase, family 61	1	10	7	7	0	0	22	18	14	0	0	1.0E+00	3.7E-01	2.5E-02	1.0E+00	5.3E-01	1.0E+00	1.0E+00	1.0E+00	1.0E+00	4.3E-01	8.1E-05	4.6E-02
IPR005201::Glycoside hydrolase, family 85	0	0	0	0	0	0	0	0	0	0	0	1.0E+00	3.7E-01	1.0E+00	1.0E+00	1.0E+00	1.0E+00	1.0E+00	1.0E+00	1.0E+00	4.3E-01	1.0E+00	1.0E+00

Peptidase family																							
IPR008257::Peptidase M19, renal dipeptidase	4	1	1	2	2	3	1	1	1	0	2	6.8E-02	3.1E-01	1.0E+00	1.0E+00	3.9E-01	1.0E+00	9.8E-01	1.0E+00	2.0E-02	3.6E-01	1.0E+00	1.0E+00
IPR005079::Peptidase C45, acyl-coenzyme A:6-aminopenicillanic acid acyl-transferase	3	2	1	1	1	2	1	1	0	0	0	8.3E-02	3.1E-01	1.0E+00	1.0E+00	3.5E-01	1.0E+00	1.0E+00	1.0E+00	3.6E-02	3.6E-01	1.0E+00	1.0E+00
IPR000819::Peptidase M17, leucyl aminopeptidase, C-terminal	0	0	0	0	0	0	0	0	0	0	1	8.8E-02	3.1E-01	9.1E-01	1.0E+00	1.0E+00	1.0E+00	1.0E+00	1.0E+00	1.7E-01	3.6E-01	8.3E-01	1.0E+00
IPR001314::Peptidase S1A, chymotrypsin	0	1	0	0	0	0	0	0	0	0	0	8.8E-02	3.1E-01	9.1E-01	1.0E+00	1.0E+00	1.0E+00	1.0E+00	1.0E+00	1.7E-01	3.6E-01	8.3E-01	1.0E+00
IPR001818::Peptidase M10, metallopeptidase	0	1	0	0	0	0	0	0	0	0	0	8.8E-02	3.1E-01	9.1E-01	1.0E+00	1.0E+00	1.0E+00	1.0E+00	1.0E+00	1.7E-01	3.6E-01	8.3E-01	1.0E+00
IPR002138::Peptidase C14, caspase non-catalytic subunit p10	0	0	0	0	0	1	0	0	0	0	0	8.8E-02	3.1E-01	9.1E-01	1.0E+00	1.0E+00	1.0E+00	1.0E+00	1.0E+00	1.7E-01	3.6E-01	1.0E+00	1.0E+00
IPR005944::Peptidase S33, proline iminopeptidase 1	0	0	0	0	0	1	0	0	0	0	0	8.8E-02	3.1E-01	9.1E-01	1.0E+00	1.0E+00	1.0E+00	1.0E+00	1.0E+00	1.7E-01	3.6E-01	1.0E+00	1.0E+00
IPR008283::Peptidase M17, leucyl aminopeptidase, N-terminal	0	0	0	0	0	0	0	0	0	0	1	8.8E-02	3.1E-01	9.1E-01	1.0E+00	1.0E+00	1.0E+00	1.0E+00	1.0E+00	1.7E-01	3.6E-01	8.3E-01	1.0E+00
IPR009090::D-aminopeptidase, middle/C-terminal	0	0	1	0	0	0	0	0	0	0	0	8.8E-02	3.1E-01	9.1E-01	1.0E+00	1.0E+00	1.0E+00	1.0E+00	1.0E+00	1.7E-01	3.6E-01	8.3E-01	1.0E+00
IPR011356::Peptidase M17	0	0	0	0	0	0	0	0	0	0	1	8.8E-02	3.1E-01	9.1E-01	1.0E+00	1.0E+00	1.0E+00	1.0E+00	1.0E+00	1.7E-01	3.6E-01	8.3E-01	1.0E+00
IPR011697::Peptidase C26	0	0	0	0	0	0	0	0	0	0	1	8.8E-02	3.1E-01	9.1E-01	1.0E+00	1.0E+00	1.0E+00	1.0E+00	1.0E+00	1.7E-01	3.6E-01	8.3E-01	1.0E+00
IPR012857::Peptidase S12, aminopeptidase DmpB, domain C	0	0	1	0	0	0	0	0	0	0	0	8.8E-02	3.1E-01	9.1E-01	1.0E+00	1.0E+00	1.0E+00	1.0E+00	1.0E+00	1.7E-01	3.6E-01	8.3E-01	1.0E+00
IPR022229::Peptidase S8A, tripeptidyl peptidase II	0	0	0	0	0	0	0	0	0	0	1	8.8E-02	3.1E-01	9.1E-01	1.0E+00	1.0E+00	1.0E+00	1.0E+00	1.0E+00	1.7E-01	3.6E-01	8.3E-01	1.0E+00
IPR002933::Peptidase M20	12	14	15	12	6	5	5	4	6	3	3	9.8E-02	3.1E-01	9.7E-01	1.0E+00	1.7E-01	1.0E+00	9.7E-01	1.0E+00	3.2E-01	3.6E-01	8.4E-01	1.0E+00
IPR011650::Peptidase M20, dimerisation	12	14	16	12	7	5	6	5	6	3	3	1.2E-01	3.1E-01	9.6E-01	1.0E+00	2.4E-01	1.0E+00	9.5E-01	1.0E+00	3.9E-01	3.6E-01	7.9E-01	1.0E+00
IPR000180::Peptidase M19, renal dipeptidase, active site	0	0	0	0	2	0	0	0	0	0	0	1.7E-01	3.1E-01	8.3E-01	1.0E+00	7.7E-01	1.0E+00	2.3E-01	1.0E+00	3.0E-01	3.6E-01	7.0E-01	1.0E+00
IPR000668::Peptidase C1A, papain C-terminal	0	0	0	0	0	0	1	1	0	0	0	1.7E-01	3.1E-01	8.3E-01	1.0E+00	1.0E+00	1.0E+00	1.0E+00	1.0E+00	3.0E-01	3.6E-01	7.0E-01	1.0E+00
IPR005321::Peptidase S58, DmpA	0	1	0	1	0	0	0	0	0	0	0	1.7E-01	3.1E-01	8.3E-01	1.0E+00	1.0E+00	1.0E+00	1.0E+00	1.0E+00	3.0E-01	3.6E-01	7.0E-01	1.0E+00
IPR005945::Peptidase S33, tricorn interacting factor 1	0	0	1	0	0	0	0	1	0	0	0	1.7E-01	3.1E-01	8.3E-01	1.0E+00	1.0E+00	1.0E+00	1.0E+00	1.0E+00	3.0E-01	3.6E-01	7.0E-01	1.0E+00
IPR006026::Peptidase, metallopeptidase	0	0	2	0	0	0	0	0	0	0	0	1.7E-01	3.1E-01	8.3E-01	1.0E+00	1.0E+00	1.0E+00	1.0E+00	1.0E+00	3.0E-01	3.6E-01	7.0E-01	1.0E+00
IPR007921::Cysteine, histidine-dependent amidohydrolase/peptidase	0	0	0	0	0	0	1	1	0	0	0	1.7E-01	3.1E-01	8.3E-01	1.0E+00	1.0E+00	1.0E+00	1.0E+00	1.0E+00	3.0E-01	3.6E-01	7.0E-01	1.0E+00
IPR012962::Peptidase M54, archaemetzincin	0	1	0	0	0	0	0	1	0	0	0	1.7E-01	3.1E-01	8.3E-01	1.0E+00	1.0E+00	1.0E+00	1.0E+00	1.0E+00	3.0E-01	3.6E-01	7.0E-01	1.0E+00
IPR012985::Peptidase S64, Ssy5	0	0	0	0	0	0	0	0	0	1	0	1.7E-01	3.1E-01	8.3E-01	1.0E+00	1.0E+00	1.0E+00	1.0E+00	1.0E+00	3.0E-01	3.6E-01	7.0E-01	1.0E+00
IPR013128::Peptidase C1A, papain	0	0	0	0	0	0	1	1	0	0	0	1.7E-01	3.1E-01	8.3E-01	1.0E+00	1.0E+00	1.0E+00	1.0E+00	1.0E+00	3.0E-01	3.6E-01	7.0E-01	1.0E+00
IPR013856::Peptidase M4, thermolysin	0	0	1	0	0	0	0	0	1	0	0	1.7E-01	3.1E-01	8.3E-01	1.0E+00	1.0E+00	1.0E+00	1.0E+00	1.0E+00	3.0E-01	3.6E-01	7.0E-01	1.0E+00
IPR010168::Peptidase M20D, amidohydrolase	4	4	6	4	2	0	2	2	2	0	0	1.9E-01	3.1E-01	9.8E-01	1.0E+00	3.9E-01	1.0E+00	9.8E-01	1.0E+00	6.5E-01	3.6E-01	7.4E-01	1.0E+00
IPR017439::Peptidase M20D, mername-AA028/carboxypeptidase Ss1	2	2	2	2	1	0	0	0	0	0	0	1.9E-01	3.1E-01	9.9E-01	1.0E+00	5.4E-01	1.0E+00	1.0E+00	1.0E+00	4.6E-01	3.6E-01	9.5E-01	1.0E+00
IPR008256::Peptidase S1B, glutamyl endopeptidase I	0	1	0	0	1	1	0	0	0	0	0	2.4E-01	3.1E-01	7.6E-01	1.0E+00	5.3E-01	1.0E+00	4.7E-01	1.0E+00	4.2E-01	3.6E-01	1.0E+00	1.0E+00
IPR008757::Peptidase M6-like, domain	0	0	0	0	1	1	0	1	0	0	0	2.4E-01	3.1E-01	7.6E-01	1.0E+00	5.3E-01	1.0E+00	4.7E-01	1.0E+00	4.2E-01	3.6E-01	1.0E+00	1.0E+00
IPR010435::Peptidase S8A, DUF1034 C-terminal	0	0	0	0	0	0	1	1	1	0	0	2.4E-01	3.1E-01	7.6E-01	1.0E+00	1.0E+00	1.0E+00	1.0E+00	1.0E+00	4.2E-01	3.6E-01	5.8E-01	1.0E+00
IPR019533::Peptidase S26, conserved region	1	0	0	0	0	0	1	1	0	0	0	2.4E-01	3.1E-01	1.0E+00	1.0E+00	5.3E-01	1.0E+00	1.0E+00	1.0E+00	4.2E-01	3.6E-01	1.0E+00	1.0E+00
IPR000169::Peptidase, cysteine peptidase active site	2	1	2	0	0	0	1	1	2	1	0	2.5E-01	3.1E-01	9.9E-01	1.0E+00	2.8E-01	1.0E+00	1.0E+00	1.0E+00	5.7E-01	3.6E-01	9.1E-01	1.0E+00
IPR000383::Peptidase S9/S15	3	4	7	1	0	0	1	2	2	0	0	2.6E-01	3.1E-01	9.7E-01	1.0E+00	1.5E-01	1.0E+00	1.0E+00	1.0E+00	6.7E-01	3.6E-01	7.7E-01	1.0E+00
IPR000718::Peptidase M13, neprilysin	1	1	0	1	0	0	0	1	0	0	0	3.1E-01	3.1E-01	1.0E+00	1.0E+00	5.3E-01	1.0E+00	1.0E+00	1.0E+00	5.2E-01	3.6E-01	9.8E-01	1.0E+00
IPR002870::Peptidase M12B, propeptide	1	0	1	0	0	0	1	1	0	0	0	3.1E-01	3.1E-01	1.0E+00	1.0E+00	5.3E-01	1.0E+00	1.0E+00	1.0E+00	5.2E-01	3.6E-01	9.8E-01	1.0E+00
IPR008753::Peptidase M13	1	1	0	1	0	0	0	1	0	0	0	3.1E-01	3.1E-01	1.0E+00	1.0E+00	5.3E-01	1.0E+00	1.0E+00	1.0E+00	5.2E-01	3.6E-01	9.8E-01	1.0E+00
IPR014766::Carboxypeptidase, regulatory domain	0	0	1	1	0	0	1	0	1	0	0	3.1E-01	3.1E-01	6.9E-01	1.0E+00	1.0E+00	1.0E+00	1.0E+00	1.0E+00	5.2E-01	3.6E-01	4.8E-01	1.0E+00
IPR018114::Peptidase S1/S6, chymotrypsin/Hap, active site	0	1	0	0	1	1	0	0	1	0	0	3.1E-01	3.1E-01	6.9E-01	1.0E+00	5.3E-01	1.0E+00	4.7E-01	1.0E+00	5.2E-01	3.6E-01	9.8E-01	1.0E+00
IPR018497::Peptidase M13, neprilysin, C-terminal	1	1	0	1	0	0	0	1	0	0	0	3.1E-01	3.1E-01	1.0E+00	1.0E+00	5.3E-01	1.0E+00	1.0E+00	1.0E+00	5.2E-01	3.6E-01	9.8E-01	1.0E+00
IPR019758::Peptidase S26A, signal peptidase I, conserved site	0	1	0	0	0	0	0	0	0	2	0	3.1E-01	3.1E-01	6.9E-01	1.0E+00	1.0E+00	1.0E+00	1.0E+00	1.0E+00	5.2E-01	3.6E-01	4.8E-01	1.0E+00
IPR012338::Beta-lactamase-type transpeptidase fold	7	8	11	6	5	6	6	7	6	0	0	3.2E-01	3.1E-01	9.0E-01	1.0E+00	4.6E-01	1.0E+00	9.0E-01	1.0E+00	2.4E-01	3.6E-01	9.1E-01	1.0E+00
IPR002410::Peptidase S33, prolyl aminopeptidase	2	1	2	0	1	2	1	3	1	0	0	3.5E-01	3.1E-01	9.7E-01	1.0E+00	5.4E-01	1.0E+00	1.0E+00	1.0E+00	1.9E-01	3.6E-01	9.8E-01	1.0E+00
IPR000816::Peptidase C15, pyroglutamyl peptidase I	1	1	0	0	1	1	1	0	0	0	0	3.7E-01	3.1E-01	9.9E-01	1.0E+00	7.7E-01	1.0E+00	1.0E+00	1.0E+00	2.0E-01	3.6E-01	1.0E+00	1.0E+00
IPR001570::Peptidase M4, thermolysin C-terminal	0	0	1	1	0	2	0	0	1	0	0	3.7E-01	3.1E-01	6.3E-01	1.0E+00	1.0E+00	1.0E+00	1.0E+00	1.0E+00	2.0E-01	3.6E-01	1.0E+00	1.0E+00
IPR001995::Peptidase A2A, retrovirus, catalytic	1	2	0	0	0	0	0	0	1	1	0	3.7E-01	3.1E-01	9.9E-01	1.0E+00	5.3E-01	1.0E+00	1.0E+00	1.0E+00	6.0E-01	3.6E-01	9.6E-01	1.0E+00
IPR017860::Peptidase M22, glycoprotease, conserved site	2	1	1	1	1	1	2	1	1	2	1	4.2E-01	3.1E-01	9.5E-01	1.0E+00	5.4E-01	1.0E+00	1.0E+00	1.0E+00	5.1E-01	3.6E-01	8.9E-01	1.0E+00
IPR001563::Peptidase S10, serine carboxypeptidase	8	5	12	11	10	11	5	5	4	3	3	4.3E-01	3.1E-01	8.2E-01	1.0E+00	8.2E-01	1.0E+00	5.1E-01	1.0E+00	7.0E-02	3.6E-01	9.8E-01	1.0E+00
IPR003146::Proteinase inhibitor, carboxypeptidase propeptide	0	0	0	0	2	2	1	0	1	0	0	4.3E-01	3.1E-01	5.7E-01	1.0E+00	7.7E-01	1.0E+00	2.3E-01	1.0E+00	2.6E-01	3.6E-01	9.9E-01	1.0E+00
IPR021860::Peptidase S12, Pab87-related, C-terminal	0	0	3	0	0	2	1	0	0	0	0	4.3E-01	3.1E-01	5.7E-01	1.0E+00	1.0E+00	1.0E+00	1.0E+00	1.0E+00	2.6E-01	3.6E-01	9.9E-01	1.0E+00
IPR005317::Peptidase M49, dipeptidyl-peptidase III	2	3	2	2	1	1	1	2	1	1	0	4.5E-01	3.1E-01	9.4E-01	1.0E+00	5.4E-01	1.0E+00	1.0E+00	1.0E+00	5.5E-01	3.6E-01	8.6E-01	1.0E+00
IPR013595::Peptidase S33, tripeptidyl-peptidase C-terminal	2	2	4	1	0	1	3	3	1	0	0	4.5E-01	3.1E-01	9.4E-01	1.0E+00	2.8E-01	1.0E+00	1.0E+00	1.0E+00	5.5E-01	3.6E-01	8.6E-01	1.0E+00
IPR013736::Peptidase S15/CocE/NonD, C-terminal	2	3	6	1	0	0	1	2	2	0	0	4.5E-01	3.1E-01	9.4E-01	1.0E+00	2.8E-01	1.0E+00	1.0E+00	1.0E+00	8.0E-01	3.8E-01	6.9E-01	1.0E+00
IPR017144::Peptidase M20D, amidohydrolase, predicted	2	2	4	2	1	0	2	2	2	0	0	4.5E-01	3.1E-01	9.4E-01	1.0E+00	5.4E-01	1.0E+00	1.0E+00	1.0E+00	8.0E-01	3.8E-01	6.9E-01	1.0E+00
IPR002471::Peptidase S9, serine active site	0	0	0	0	1	2	0	0	1	1	1	4.8E-01	3.1E-01	5.2E-01	1.0E+00	5.3E-01	1.0E+00	4.7E-01	1.0E+00	3.3E-01	3.6E-01	9.8E-01	1.0E+00
IPR004134::Peptidase C1B, bleomycin hydrolase	1	1	1	0	0	0	1	0	1	1	0	4.8E-01	3.1E-01	9.8E-01	1.0E+00	5.3E-01	1.0E+00	1.0E+00	1.0E+00	7.2E-01	3.6E-01	9.1E-01	1.0E+00
IPR005151::Peptidase S41	1	0	0	1	1	3	0	0	1	0	0	4.8E-01	3.1E-01	9.8E-01	1.0E+00	7.7E-01	1.0E+00	1.0E+00	1.0E+00	1.7E-02	3.6E-01	1.0E+00	1.0E+00
IPR018349::Peptidase M24A, methionine aminopeptidase, subfamily 2, binding site	2	2	2	2	2	2	1	1	1	1	1	4.8E-01	3.1E-01	9.3E-01	1.0E+00	7.2E-01	1.0E+00	9.2E-01	1.0E+00	3.5E-01	3.6E-01	9.4E-01	1.0E+00
IPR019757::Peptidase S26A, signal peptidase I, lysine active site	0	0	0	0	1	0	1	1	1	1	1	4.8E-01	3.1E-01	5.2E-01	1.0E+00	5.3E-01	1.0E+00	4.7E-01	1.0E+00	7.2E-01	3.6E-01	2.8E-01	1.0E+00
IPR003111::Peptidase S16, lon N-terminal	3	3	3	3	3	4	2	2	2	1	2	5.0E-01	3.1E-01	8.8E-01	1.0E+00	7.0E-01	1.0E+00	8.6E-01	1.0E+00	2.2E-01	3.6E-01	9.5E-01	1.0E+00
IPR019400::Peptidase C65, otubain	1	1	0	1	1	1	1	1	1	0	0	5.2E-01	3.1E-01	9.7E-01	1.0E+00	7.7E-01	1.0E+00	1.0E+00	1.0E+00	3.9E-01	3.6E-01	9.7E-01	1.0E+00
IPR001578::Peptidase C12, ubiquitin carboxyl-terminal hydrolase 1	4	4	5	4	4	5	4	5	3	1	2	5.3E-01	3.1E-01	8.2E-01	1.0E+00	6.9E-01	1.0E+00	8.2E-01	1.0E+00	2.8E-01	3.6E-01	9.1E-01	1.0E+00
IPR018202::Peptidase S10, serine carboxypeptidase, active site	6	5	10	9	8	9	3	4	4	3	2	5.5E-01	3.1E-01	7.6E-01	1.0E+00	8.4E-01	1.0E+00	5.3E-01	1.0E+00	1.4E-01	3.6E-01	9.6E-01	1.0E+00
IPR001907::Peptidase S14, ClpP	1	1	1	1	1	1	1	1	1	0	0	5.6E-01	3.1E-01	9.6E-01	1.0E+00	7.7E-01	1.0E+00	1.0E+00	1.0E+00	4.6E-01	3.6E-01	9.5E-01	1.0E+00
IPR002468::Peptidase M24A, methionine aminopeptidase, subfamily 2	2	2	2	3	2	2	2	2	1	1	1	5.6E-01	3.1E-01	8.9E-01	1.0E+00	7.2E-01	1.0E+00	9.2E-01	1.0E+00	4.7E-01	3.6E-01	8.8E-01	1.0E+00
IPR004815::Peptidase S16, ATP-dependent protease La	2	2	2	2	2	2	2	2	2	1	1	5.6E-01	3.1E-01	8.9E-01	1.0E+00	7.2E-01	1.0E+00	9.2E-01	1.0E+00	4.7E-01	3.6E-01	8.8E-01	1.0E+00
IPR011096::Propeptide, peptidase M4/M36	0	0	1	1	2	5	0	0	0	0	0	5.6E-01	3.1E-01	4.4E-01	1.0E+00	7.7E-01	1.0E+00	2.3E-01	1.0E+00	8.8E-03	3.6E-01	1.0E+00	1.0E+00
IPR016125::Peptidase C15, pyroglutamyl peptidase I-like	1	1	1	1	1	1	1	1	1	0	0	5.6E-01	3.1E-01	9.6E-01	1.0E+00	7.7E-01	1.0E+00	1.0E+00	1.0E+00	4.6E-01	3.6E-01	9.5E-01	1.0E+00
IPR018215::Peptidase S14, ClpP, active site	1	1	1	1	1	1	1	1	1	0	0	5.6E-01	3.1E-01	9.6E-01	1.0E+00	7.7E-01	1.0E+00	1.0E+00	1.0E+00	4.6E-01	3.6E-01	9.5E-01	1.0E+00
IPR019103::Peptidase aspartic, eukaryotic predicted	0	1	1	1	1	1	0	0	1	1	1	5.6E-01	3.1E-01	4.4E-01	1.0E+00	5.3E-01	1.0E+00	4.7E-01	1.0E+00	8.0E-01	3.8E-01	8.2E-01	1.0E+00
IPR022683::Peptidase C2, calpain, domain III	1	1	1	1	1	1	1	1	1	0	0	5.6E-01	3.1E-01	9.6E-01	1.0E+00	7.7E-01	1.0E+00	1.0E+00	1.0E+00	4.6E-01	3.6E-01	9.5E-01	1.0E+00
IPR000223::Peptidase S26A, signal peptidase I	2	2	2	2	2	1	2	1	2	2	2	5.9E-01	3.1E-01	8.8E-01	1.0E+00	7.2E-01	1.0E+00	9.2E-01	1.0E+00	7.3E-01	3.6E-01	7.0E-01	1.0E+00
IPR000246::Peptidase T2, asparaginase 2	2	2	1	2	2	2	3	3	3	0	1	5.9E-01	3.1E-01	8.8E-01	1.0E+00	7.2E-01	1.0E+00	9.2E-01	1.0E+00	5.1E-01	3.6E-01	8.5E-01	1.0E+00
IPR001394::Peptidase C19, ubiquitin carboxyl-terminal hydrolase 2	17	17	17	17	18	17	15	15	15	18	16	5.9E-01	3.1E-01	6.1E-01	1.0E+00	7.4E-01	1.0E+00	5.1E-01	1.0E+00	4.4E-01	3.6E-01	7.0E-01	1.0E+00
IPR001915::Peptidase M48	2	2	2	2	1	1	2	2	2	2	2	5.9E-01	3.1E-01	8.8E-01	1.0E+00	5.4E-01	1.0E+00	1.0E+00	1.0E+00	7.3E-01	3.6E-01	7.0E-01	1.0E+00
IPR001930::Peptidase M1, Alanine aminopeptidase/Leukotriene A4 hydrolase	3	3	3	2	3	2	3	3	3	4	2	5.9E-01	3.1E-01	8.2E-01	1.0E+00	7.0E-01	1.0E+00	8.6E-01	1.0E+00	6.9E-01	3.6E-01	6.7E-01	1.0E+00
IPR008269::Peptidase S16, Lon C-terminal	2	2	2	2	2	2	2	2	2	1	1	5.9E-01	3.1E-01	8.8E-01	1.0E+00	7.2E-01	1.0E+00	9.2E-01	1.0E+00	5.1E-01	3.6E-01	8.5E-01	1.0E+00
IPR008758::Peptidase S28	2	2	3	3	3	2	3	2	2	0	0	5.9E-01	3.1E-01	8.8E-01	1.0E+00	8.4E-01	1.0E+00	7.8E-01	1.0E+00	5.1E-01	3.6E-01	8.5E-01	1.0E+00
IPR001254::Peptidase S1/S6, chymotrypsin/Hap	0	2	1	1	2	2	0	0	1	0	0	6.0E-01	3.1E-01	4.0E-01	1.0E+00	7.7E-01	1.0E+00	2.3E-01	1.0E+00	5.1E-01	3.6E-01	9.3E-01	1.0E+00
IPR001842::Peptidase M36, fungalysin	0	0	1	1	2	6	0	0	0	0	0	6.0E-01	3.1E-01	4.0E-01	1.0E+00	7.7E-01	1.0E+00	2.3E-01	1.0E+00	2.4E-03	3.6E-01	1.0E+00	1.0E+00
IPR008442::Propeptide, carboxypeptidase Y	1	1	1	1	1	0	1	1	1	1	0	6.0E-01	3.1E-01	9.5E-01	1.0E+00	7.7E-01	1.0E+00	1.0E+00	1.0E+00	8.4E-01	3.9E-01	7.8E-01	1.0E+00
IPR012462::Peptidase C78, ubiquitin fold modifier-specific peptidase 1/ 2	1	1	1	1	1	1	1	1	1	0	1	6.0E-01	3.1E-01	9.5E-01	1.0E+00	7.7E-01	1.0E+00	1.0E+00	1.0E+00	5.1E-01	3.6E-01	9.3E-01	1.0E+00
IPR015211::Peptidase M1, leukotriene A4 hydrolase, aminopeptidase C-terminal	1	1	1	0	1	0	1	1	1	1	1	6.0E-01	3.1E-01	9.5E-01	1.0E+00	7.7E-01	1.0E+00	1.0E+00	1.0E+00	8.4E-01	3.9E-01	7.8E-01	1.0E+00
IPR022682::Peptidase C2, calpain, large subunit, domain III	1	1	1	1	1	1	1	1	1	0	0	6.0E-01	3.1E-01	9.5E-01	1.0E+00	7.7E-01	1.0E+00	1.0E+00	1.0E+00	5.1E-01	3.6E-01	9.3E-01	1.0E+00
IPR022684::Peptidase C2, calpain family	1	2	1	1	1	1	1	1	1	0	0	6.0E-01	3.1E-01	9.5E-01	1.0E+00	7.7E-01	1.0E+00	1.0E+00	1.0E+00	5.1E-01	3.6E-01	9.3E-01	1.0E+00
IPR000243::Peptidase T1A, proteasome beta-subunit	3	3	3	3	3	3	3	3	3	3	2	6.1E-01	3.1E-01	8.1E-01	1.0E+00	7.0E-01	1.0E+00	8.6E-01	1.0E+00	5.4E-01	3.6E-01	7.8E-01	1.0E+00
IPR001714::Peptidase M24, methionine aminopeptidase	3	4	4	5	3	2	3	3	2	2	2	6.1E-01	3.1E-01	8.1E-01	1.0E+00	7.0E-01	1.0E+00	8.6E-01	1.0E+00	7.1E-01	3.6E-01	6.4E-01	1.0E+00
IPR001300::Peptidase C2, calpain, catalytic domain	2	2	2	2	1	1	3	4	4	1	0	6.2E-01	3.1E-01	8.6E-01	1.0E+00	5.4E-01	1.0E+00	1.0E+00	1.0E+00	7.6E-01	3.7E-01	6.7E-01	1.0E+00
IPR014782::Peptidase M1, membrane alanine aminopeptidase, N-terminal	3	3	3	2	3	2	3	3	3	4	3	6.3E-01	3.1E-01	7.9E-01	1.0E+00	7.0E-01	1.0E+00	8.6E-01	1.0E+00	7.4E-01	3.6E-01	6.1E-01	1.0E+00
IPR000905::Peptidase M22, glycoprotease	2	2	2	2	2	2	2	2	2	2	2	6.4E-01	3.1E-01	8.4E-01	1.0E+00	7.2E-01	1.0E+00	9.2E-01	1.0E+00	5.8E-01	3.6E-01	8.0E-01	1.0E+00
IPR001940::Peptidase S1C, HrtA/DegP2/Q/S	1	1	1	1	1	1	1	1	1	1	1	6.4E-01	3.1E-01	9.3E-01	1.0E+00	7.7E-01	1.0E+00	1.0E+00	1.0E+00	5.7E-01	3.6E-01	9.1E-01	1.0E+00
IPR002610::Peptidase S54, rhomboid	2	2	2	2	2	2	2	2	3	1	2	6.4E-01	3.1E-01	8.4E-01	1.0E+00	7.2E-01	1.0E+00	9.2E-01	1.0E+00	5.8E-01	3.6E-01	8.0E-01	1.0E+00
IPR006639::Peptidase A22, presenilin signal peptide	1	1	1	1	1	1	1	1	1	1	1	6.4E-01	3.1E-01	9.3E-01	1.0E+00	7.7E-01	1.0E+00	1.0E+00	1.0E+00	5.7E-01	3.6E-01	9.1E-01	1.0E+00
IPR007369::Peptidase A22B, signal peptide peptidase	1	1	1	1	1	1	1	1	1	1	1	6.4E-01	3.1E-01	9.3E-01	1.0E+00	7.7E-01	1.0E+00	1.0E+00	1.0E+00	5.7E-01	3.6E-01	9.1E-01	1.0E+00
IPR008754::Peptidase M43, pregnancy-associated plasma-A	1	2	1	1	1	2	1	0	2	0	0	6.4E-01	3.1E-01	9.3E-01	1.0E+00	7.7E-01	1.0E+00	1.0E+00	1.0E+00	2.7E-01	3.6E-01	9.8E-01	1.0E+00
IPR009542::Microsomal signal peptidase 12kDa subunit	1	1	1	1	1	1	1	1	1	1	1	6.4E-01	3.1E-01	9.3E-01	1.0E+00	7.7E-01	1.0E+00	1.0E+00	1.0E+00	5.7E-01	3.6E-01	9.1E-01	1.0E+00
IPR011600::Peptidase C14, caspase catalytic	2	4	3	2	1	2	2	2	3	1	1	6.4E-01	3.1E-01	8.4E-01	1.0E+00	5.4E-01	1.0E+00	1.0E+00	1.0E+00	5.8E-01	3.6E-01	8.0E-01	1.0E+00
IPR016295::Proteasome endopeptidase complex, beta subunit	1	1	1	1	1	1	0	1	1	1	1	6.4E-01	3.1E-01	9.3E-01	1.0E+00	7.7E-01	1.0E+00	1.0E+00	1.0E+00	5.7E-01	3.6E-01	9.1E-01	1.0E+00
IPR017861::Peptidase M22, glycoprotease, subgroup	2	2	2	2	2	2	2	2	2	2	2	6.4E-01	3.1E-01	8.4E-01	1.0E+00	7.2E-01	1.0E+00	9.2E-01	1.0E+00	5.8E-01	3.6E-01	8.0E-01	1.0E+00
IPR015927::Peptidase S24/S26A/S26B/S26C	3	3	3	3	4	3	3	3	3	3	3	6.5E-01	3.1E-01	7.7E-01	1.0E+00	8.1E-01	1.0E+00	7.3E-01	1.0E+00	5.9E-01	3.6E-01	7.4E-01	1.0E+00
IPR022764::Peptidase S54, rhomboid domain	3	3	3	3	3	3	3	3	4	2	4	6.5E-01	3.1E-01	7.7E-01	1.0E+00	7.0E-01	1.0E+00	8.6E-01	1.0E+00	5.9E-01	3.6E-01	7.4E-01	1.0E+00
IPR001567::Peptidase M3A/M3B, thimet/oligopeptidase F	2	1	4	2	2	2	2	3	2	2	1	6.6E-01	3.1E-01	8.3E-01	1.0E+00	7.2E-01	1.0E+00	9.2E-01	1.0E+00	6.2E-01	3.6E-01	7.7E-01	1.0E+00
IPR001948::Peptidase M18	2	3	2	2	2	2	2	2	2	2	1	6.6E-01	3.1E-01	8.3E-01	1.0E+00	7.2E-01	1.0E+00	9.2E-01	1.0E+00	6.2E-01	3.6E-01	7.7E-01	1.0E+00
IPR018200::Peptidase C19, ubiquitin carboxyl-terminal hydrolase 2, conserved site	12	11	11	12	14	13	11	10	11	16	14	6.6E-01	3.1E-01	5.6E-01	1.0E+00	8.0E-01	1.0E+00	4.7E-01	1.0E+00	5.0E-01	3.6E-01	6.7E-01	1.0E+00
IPR001096::Peptidase C13, legumain	1	1	1	1	1	1	1	1	1	1	1	6.7E-01	3.1E-01	9.2E-01	1.0E+00	7.7E-01	1.0E+00	1.0E+00	1.0E+00	6.2E-01	3.6E-01	8.8E-01	1.0E+00
IPR001590::Peptidase M12B, ADAM/reprolysin	1	1	2	2	1	1	1	1	1	0	1	6.7E-01	3.1E-01	9.2E-01	1.0E+00	7.7E-01	1.0E+00	1.0E+00	1.0E+00	6.2E-01	3.6E-01	8.8E-01	1.0E+00
IPR005078::Peptidase C54	1	1	1	1	1	1	1	1	1	1	1	6.7E-01	3.1E-01	9.2E-01	1.0E+00	7.7E-01	1.0E+00	1.0E+00	1.0E+00	6.2E-01	3.6E-01	8.8E-01	1.0E+00
IPR005314::Peptidase C50, separase	1	1	1	1	1	1	1	1	1	1	1	6.7E-01	3.1E-01	9.2E-01	1.0E+00	7.7E-01	1.0E+00	1.0E+00	1.0E+00	6.2E-01	3.6E-01	8.8E-01	1.0E+00
IPR007653::Signal peptidase 22kDa subunit	1	1	1	1	1	1	1	1	1	1	1	6.7E-01	3.1E-01	9.2E-01	1.0E+00	7.7E-01	1.0E+00	1.0E+00	1.0E+00	6.2E-01	3.6E-01	8.8E-01	1.0E+00
IPR007863::Peptidase M16, C-terminal	6	6	7	6	6	6	6	6	6	8	6	6.7E-01	3.1E-01	6.4E-01	1.0E+00	6.8E-01	1.0E+00	7.5E-01	1.0E+00	6.2E-01	3.6E-01	6.2E-01	1.0E+00
IPR008268::Peptidase S16, active site	1	1	1	1	1	1	1	1	1	1	1	6.7E-01	3.1E-01	9.2E-01	1.0E+00	7.7E-01	1.0E+00	1.0E+00	1.0E+00	6.2E-01	3.6E-01	8.8E-01	1.0E+00
IPR009582::Signal peptidase complex subunit 2	1	1	1	1	1	1	1	1	1	1	1	6.7E-01	3.1E-01	9.2E-01	1.0E+00	7.7E-01	1.0E+00	1.0E+00	1.0E+00	6.2E-01	3.6E-01	8.8E-01	1.0E+00
IPR011765::Peptidase M16, N-terminal	6	6	7	6	5	6	6	6	6	8	6	6.7E-01	3.1E-01	6.4E-01	1.0E+00	5.7E-01	1.0E+00	8.5E-01	1.0E+00	6.2E-01	3.6E-01	6.2E-01	1.0E+00
IPR013578::Peptidase M16C associated	1	1	1	1	1	1	1	1	1	1	1	6.7E-01	3.1E-01	9.2E-01	1.0E+00	7.7E-01	1.0E+00	1.0E+00	1.0E+00	6.2E-01	3.6E-01	8.8E-01	1.0E+00
IPR016526::Peptidase M49, dipeptidyl-peptidase 3, eukaryotic	1	1	1	2	1	1	1	1	1	1	0	6.7E-01	3.1E-01	9.2E-01	1.0E+00	7.7E-01	1.0E+00	1.0E+00	1.0E+00	6.2E-01	3.6E-01	8.8E-01	1.0E+00
IPR017141::Peptidase M20, carboxypeptidase S	1	2	1	1	0	0	1	1	1	1	1	6.7E-01	3.1E-01	9.2E-01	1.0E+00	5.3E-01	1.0E+00	1.0E+00	1.0E+00	8.9E-01	4.0E-01	6.8E-01	1.0E+00
IPR019165::Peptidase M76, ATP23	1	1	1	1	1	1	1	1	1	1	1	6.7E-01	3.1E-01	9.2E-01	1.0E+00	7.7E-01	1.0E+00	1.0E+00	1.0E+00	6.2E-01	3.6E-01	8.8E-01	1.0E+00
IPR022495::Kinase-associated endopeptidase 1, Bud32	1	1	1	1	1	1	1	1	1	1	1	6.7E-01	3.1E-01	9.2E-01	1.0E+00	7.7E-01	1.0E+00	1.0E+00	1.0E+00	6.2E-01	3.6E-01	8.8E-01	1.0E+00
IPR000101::Gamma-glutamyltranspeptidase	2	2	4	2	2	3	2	2	2	1	2	6.8E-01	3.1E-01	8.1E-01	1.0E+00	7.2E-01	1.0E+00	9.2E-01	1.0E+00	4.4E-01	3.6E-01	8.7E-01	1.0E+00
IPR000642::Peptidase M41	2	2	2	2	2	2	2	2	2	3	2	6.8E-01	3.1E-01	8.1E-01	1.0E+00	7.2E-01	1.0E+00	9.2E-01	1.0E+00	6.5E-01	3.6E-01	7.4E-01	1.0E+00
IPR001984::Peptidase S16, Lon protease, C-terminal	2	2	2	2	2	2	3	2	4	1	1	6.8E-01	3.1E-01	8.1E-01	1.0E+00	7.2E-01	1.0E+00	9.2E-01	1.0E+00	6.5E-01	3.6E-01	7.4E-01	1.0E+00
IPR005936::Peptidase M41, FtsH	2	2	2	2	2	2	2	2	2	3	2	6.8E-01	3.1E-01	8.1E-01	1.0E+00	7.2E-01	1.0E+00	9.2E-01	1.0E+00	6.5E-01	3.6E-01	7.4E-01	1.0E+00
IPR011237::Peptidase M16, core	6	6	7	6	6	6	6	6	6	8	6	6.8E-01	3.1E-01	6.3E-01	1.0E+00	6.8E-01	1.0E+00	7.5E-01	1.0E+00	6.4E-01	3.6E-01	6.0E-01	1.0E+00
IPR001431::Peptidase M16, zinc-binding site	2	2	2	2	2	2	2	2	2	5	3	7.0E-01	3.1E-01	7.9E-01	1.0E+00	7.2E-01	1.0E+00	9.2E-01	1.0E+00	6.8E-01	3.6E-01	7.1E-01	1.0E+00
IPR001733::Peptidase S26B, eukaryotic signal peptidase	1	1	1	1	2	1	1	1	1	1	1	7.0E-01	3.1E-01	9.0E-01	1.0E+00	8.9E-01	1.0E+00	8.5E-01	1.0E+00	6.6E-01	3.6E-01	8.4E-01	1.0E+00
IPR006615::Peptidase C19, ubiquitin-specific peptidase, DUSP domain	1	1	1	1	1	1	1	1	1	1	2	7.0E-01	3.1E-01	9.0E-01	1.0E+00	7.7E-01	1.0E+00	1.0E+00	1.0E+00	6.6E-01	3.6E-01	8.4E-01	1.0E+00
IPR021917::Uncharacterised protein family, zinc metallopeptidase-like	1	1	1	1	1	1	2	1	1	1	1	7.0E-01	3.1E-01	9.0E-01	1.0E+00	7.7E-01	1.0E+00	1.0E+00	1.0E+00	6.6E-01	3.6E-01	8.4E-01	1.0E+00
IPR011249::Metalloenzyme, LuxS/M16 peptidase-like, metal-binding	6	6	7	7	6	6	7	6	6	8	6	7.1E-01	3.1E-01	6.0E-01	1.0E+00	6.8E-01	1.0E+00	7.5E-01	1.0E+00	6.8E-01	3.6E-01	5.6E-01	1.0E+00
IPR003653::Peptidase C48, SUMO/Sentrin/Ubl1	3	4	3	3	6	3	3	4	4	2	4	7.3E-01	3.1E-01	6.9E-01	1.0E+00	9.3E-01	1.0E+00	4.4E-01	1.0E+00	7.2E-01	3.6E-01	6.0E-01	1.0E+00
IPR011546::Peptidase M41, FtsH extracellular	1	1	1	1	1	1	1	1	1	2	1	7.3E-01	3.1E-01	8.8E-01	1.0E+00	7.7E-01	1.0E+00	1.0E+00	1.0E+00	7.0E-01	3.6E-01	8.1E-01	1.0E+00
IPR016117::Peptidase S58 DmpA/arginine biosynthesis protein ArgJ	1	2	1	2	1	1	1	1	1	1	1	7.3E-01	3.1E-01	8.8E-01	1.0E+00	7.7E-01	1.0E+00	1.0E+00	1.0E+00	7.0E-01	3.6E-01	8.1E-01	1.0E+00
IPR000250::Peptidase G1, eqolisin	0	2	4	2	0	0	2	4	1	0	0	7.5E-01	3.1E-01	2.5E-01	1.0E+00	1.0E+00	1.0E+00	1.0E+00	1.0E+00	9.3E-01	4.2E-01	6.6E-02	1.0E+00

IPR007484::Peptidase M28	6	7	7	7	9	11	9	7	8	5	3	7.6E-01	3.1E-01	5.3E-01	1.0E+00	8.9E-01	1.0E+00	4.2E-01	1.0E+00	2.2E-01	3.6E-01	9.1E-01	1.0E+00
IPR002467::Peptidase M24A, methionine aminopeptidase, subfamily 1	1	3	2	2	1	1	1	1	1	1	1	7.7E-01	3.1E-01	8.4E-01	1.0E+00	7.7E-01	1.0E+00	1.0E+00	1.0E+00	7.7E-01	3.7E-01	7.3E-01	1.0E+00
IPR007230::Peptidase S59, nucleoporin	1	1	1	1	1	1	1	1	1	3	2	7.7E-01	3.1E-01	8.4E-01	1.0E+00	7.7E-01	1.0E+00	1.0E+00	1.0E+00	7.7E-01	3.7E-01	7.3E-01	1.0E+00
IPR007865::Peptidase M24B, X-Pro dipeptidase/aminopeptidase P N-terminal	2	3	3	3	3	3	3	3	3	2	1	7.7E-01	3.1E-01	7.1E-01	1.0E+00	8.4E-01	1.0E+00	7.8E-01	1.0E+00	6.0E-01	3.6E-01	7.5E-01	1.0E+00
IPR009007::Peptidase aspartic, catalytic	11	10	14	8	6	9	18	26	19	10	2	7.7E-01	3.1E-01	4.4E-01	1.0E+00	2.2E-01	1.0E+00	9.6E-01	1.0E+00	8.8E-01	4.0E-01	2.4E-01	1.0E+00
IPR019756::Peptidase S26A, signal peptidase I, serine active site	1	1	1	1	2	1	1	1	1	3	0	7.7E-01	3.1E-01	8.4E-01	1.0E+00	8.9E-01	1.0E+00	8.5E-01	1.0E+00	7.7E-01	3.7E-01	7.3E-01	1.0E+00
IPR000994::Peptidase M24, structural domain	8	12	12	11	9	9	9	9	9	7	8	7.8E-01	3.1E-01	4.7E-01	1.0E+00	7.6E-01	1.0E+00	6.1E-01	1.0E+00	7.0E-01	3.6E-01	5.0E-01	1.0E+00
IPR001375::Peptidase S9, prolyl oligopeptidase, catalytic domain	4	10	9	6	6	5	4	7	3	2	3	8.0E-01	3.2E-01	5.5E-01	1.0E+00	8.7E-01	1.0E+00	5.6E-01	1.0E+00	7.0E-01	3.6E-01	5.7E-01	1.0E+00
IPR001461::Peptidase A1	10	10	13	7	6	8	18	24	18	9	2	8.0E-01	3.2E-01	4.1E-01	1.0E+00	2.9E-01	1.0E+00	9.4E-01	1.0E+00	9.1E-01	4.1E-01	2.0E-01	1.0E+00
IPR019759::Peptidase S24/S26A/S26B, conserved region	2	3	3	3	4	2	3	2	3	3	3	8.1E-01	3.2E-01	6.5E-01	1.0E+00	9.1E-01	1.0E+00	6.1E-01	1.0E+00	8.4E-01	3.9E-01	4.9E-01	1.0E+00
IPR021109::Peptidase aspartic	11	11	15	9	7	10	18	25	20	11	3	8.2E-01	3.2E-01	3.7E-01	1.0E+00	3.1E-01	1.0E+00	9.3E-01	1.0E+00	8.8E-01	4.0E-01	2.3E-01	1.0E+00
IPR002469::Peptidase S9B, dipeptidylpeptidase IV N-terminal	1	2	1	2	2	2	2	1	1	2	2	8.4E-01	3.3E-01	7.4E-01	1.0E+00	8.9E-01	1.0E+00	8.5E-01	1.0E+00	6.7E-01	3.6E-01	7.7E-01	1.0E+00
IPR011056::Peptidase S24/S26A/S26B/S26C, beta-ribbon domain	2	3	3	3	4	3	3	3	3	3	3	8.4E-01	3.3E-01	6.1E-01	1.0E+00	9.1E-01	1.0E+00	6.1E-01	1.0E+00	7.4E-01	3.6E-01	6.1E-01	1.0E+00
IPR001384::Peptidase M35, deuterolysin	0	4	0	3	7	5	0	0	2	0	0	8.6E-01	3.3E-01	1.4E-01	1.0E+00	9.9E-01	1.0E+00	5.4E-03	1.0E+00	2.6E-01	3.6E-01	9.5E-01	1.0E+00
IPR000834::Peptidase M14, carboxypeptidase A	1	2	1	1	3	4	3	2	2	1	1	8.7E-01	3.4E-01	6.9E-01	1.0E+00	9.5E-01	1.0E+00	6.5E-01	1.0E+00	3.0E-01	3.6E-01	9.4E-01	1.0E+00
IPR001131::Peptidase M24B, X-Pro dipeptidase/aminopeptidase P, conserved site	1	2	2	2	2	2	2	3	2	3	3	9.0E-01	3.5E-01	6.2E-01	1.0E+00	8.9E-01	1.0E+00	8.5E-01	1.0E+00	8.1E-01	3.8E-01	6.0E-01	1.0E+00
IPR009003::Serine/cysteine peptidase, trypsin-like	1	3	1	2	2	9	2	1	3	2	2	9.4E-01	3.6E-01	5.0E-01	1.0E+00	8.9E-01	1.0E+00	8.5E-01	1.0E+00	1.9E-02	3.6E-01	1.0E+00	1.0E+00
IPR001969::Peptidase aspartic, active site	7	5	7	5	4	5	9	16	11	34	14	9.5E-01	3.6E-01	1.7E-01	1.0E+00	3.3E-01	1.0E+00	9.5E-01	1.0E+00	9.9E-01	4.3E-01	2.1E-02	1.0E+00
IPR015366::Peptidase S53, propeptide	1	3	7	5	3	3	3	4	3	0	0	9.5E-01	3.6E-01	4.5E-01	1.0E+00	9.5E-01	1.0E+00	6.5E-01	1.0E+00	8.0E-01	3.8E-01	5.6E-01	1.0E+00
IPR015500::Peptidase S8, subtilisin-related	3	4	9	5	18	17	8	9	7	4	4	9.9E-01	3.7E-01	7.8E-02	1.0E+00	1.0E+00	1.0E+00	1.8E-03	1.0E+00	1.3E-01	3.6E-01	9.5E-01	1.0E+00
IPR022398::Peptidase S8/S53, subtilisin, active site	3	4	8	6	17	17	10	11	9	4	4	9.9E-01	3.7E-01	5.9E-02	1.0E+00	1.0E+00	1.0E+00	3.0E-03	1.0E+00	1.9E-01	3.6E-01	9.2E-01	1.0E+00
IPR000209::Peptidase S8/S53, subtilisin/kexin/sedolisin	3	7	16	10	21	19	13	14	11	4	4	1.0E+00	3.7E-01	1.0E-02	1.0E+00	1.0E+00	1.0E+00	3.2E-04	5.0E-01	4.5E-01	3.6E-01	7.2E-01	1.0E+00
IPR002142::Peptidase S49	0	0	0	0	0	0	0	0	0	0	0	1.0E+00	3.7E-01	1.0E+00	1.0E+00	1.0E+00	1.0E+00	1.0E+00	1.0E+00	1.0E+00	4.3E-01	1.0E+00	1.0E+00
IPR002470::Peptidase S9A, prolyl oligopeptidase	0	0	0	0	0	0	0	0	0	0	0	1.0E+00	3.7E-01	1.0E+00	1.0E+00	1.0E+00	1.0E+00	1.0E+00	1.0E+00	1.0E+00	4.3E-01	1.0E+00	1.0E+00
IPR004106::Peptidase S9A, oligopeptidase, N-terminal beta-propeller	0	0	0	0	0	0	0	0	0	0	0	1.0E+00	3.7E-01	1.0E+00	1.0E+00	1.0E+00	1.0E+00	1.0E+00	1.0E+00	1.0E+00	4.3E-01	1.0E+00	1.0E+00
IPR005322::Peptidase C69, dipeptidase A	0	0	0	0	0	0	0	0	0	0	0	1.0E+00	3.7E-01	1.0E+00	1.0E+00	1.0E+00	1.0E+00	1.0E+00	1.0E+00	1.0E+00	4.3E-01	1.0E+00	1.0E+00
IPR008915::Peptidase M50	0	0	0	0	0	0	0	0	0	0	0	1.0E+00	3.7E-01	1.0E+00	1.0E+00	1.0E+00	1.0E+00	1.0E+00	1.0E+00	1.0E+00	4.3E-01	1.0E+00	1.0E+00

Table S2 Cell wall genes in *W. dermatitidis* and other fungal genomes^.

Gene ID*	Number of genes in species											pH		Radiation		Gene symbol#	Gene description#
	W. dermatitidis	*A. niger*	*A. fumigatus*	*A. nidulans*	*C. immitis*	*T. rubrum*	*N. crassa*	*M. thermophila*	*T. terrestris*	*S. cerevisiae*	*S. pombe*	$\log_2$ Fold**	P-value***	$\log_2$ Fold**	P-value***		
**Chitin synthase**																	
HMPREF1120_06816&	2	2	2	2	2	2	2	2	2	2	1	1.75	7.17E-28	0.49	2.88E-04	CHS2	Class I chitin synthase
HMPREF1120_07981&	paralog&											0.38	2.19E-02	-1.73	1.68E-24	CHS1	Class II chitin synthase
HMPREF1120_06479	1	3	2	2	1	1	1	1	1	0	0	1.86	3.01E-32	0.43	1.35E-03	CHS3	Class III chitin synthase
HMPREF1120_07721	1	1	1	1	1	1	1	1	1	1	0	1.32	2.77E-17	-0.23	9.57E-02	CHS4	Class IV chitin synthase
HMPREF1120_08776	2	2	2	2	2	2	2	2	2	0	0	1.31	3.54E-17	-0.68	1.18E-07	CHS5	Class V chitin synthase
HMPREF1120_08777	paralog											1.48	1.82E-21	-0.65	3.85E-07	CHS7	Class VII chitin synthase
HMPREF1120_09115	1	1	1	1	1	1	1	1	1	0	0	0.04	8.82E-01	-0.34	1.22E-02	CHS6	Class VI chitin synthase
HMPREF1120_01791	1	0	0	0	0	0	0	0	0	0	0	-2.21	1.60E-41	-3.13	2.33E-119		Chitin synthase like
HMPREF1120_01790	1	0	0	0	0	0	0	0	0	0	0	-1.89	8.44E-30	-3.08	3.34E-118		UDP-N-acetylglucosamine 6-dehydrogenase
**Regulation of chitin synthase activity, by analogy to S. cerevisiae**																	
HMPREF1120_07720	1	1	1	1	1	1	1	1	1	1	0	1.73	2.89E-28	-0.39	3.43E-03	SKT5	Activator of Chs3p during vegetative growth
HMPREF1120_06335	1	1	1	1	1	1	1	1	1	0	1	1.55	9.08E-23	-0.1	5.03E-01		Similarity with ScSkt5, activator of Chs3
HMPREF1120_05528	1	1	1	1	1	1	1	1	1	0	1	-0.44	7.54E-03	-0.03	8.78E-01		Similarity with ScSkt5, activator of Chs3
HMPREF1120_05249	1	1	1	1	1	1	1	1	1	1	0	0.77	1.12E-06	-0.27	4.54E-02	BNI4	scaffold protein that tethers chitin synthase III (Chs3p) to the bud neck
HMPREF1120_05359	1	1	1	1	1	1	1	1	1	1	1	0.09	6.35E-01	-0.82	2.28E-10	ScCHS5	Similarity with ScChs5, component of exomer complex
HMPREF1120_01856	1	1	1	1	1	1	1	1	1	1	1	-0.14	4.40E-01	0.09	5.54E-01	ScCHS6	Similarity with ScChs6, component of exomer complex
HMPREF1120_00837	1	1	1	1	1	1	1	1	1	1	0	1.37	4.24E-18	0.47	4.40E-04		Similarity with export control protein ScChs7
HMPREF1120_03003	1	1	1	1	1	1	1	1	1	0	0	0.51	1.97E-03	0.38	7.36E-03	ScCHS7	Similarity with export control protein ScChs7
**Chitin modification**																	
HMPREF1120_08023	1	1	1	1	1	1	2	2	2	2	0	0.21	2.21E-01	-0.4	4.07E-03	Cda1/2	Chitin deacetylase
HMPREF1120_01911	1	1	1	1	1	1	1	1	1	0	0	1.63	5.46E-14	-0.87	1.72E-04		
**Chitin degradation**																	
HMPREF1120_03399	1	1	1	1	1	1	1	1	1	0	1	-0.24	1.60E-01	-0.73	3.68E-06	ChiA	GPI anchored class III chitinase
HMPREF1120_02334	1	1	1	1	1	1	1	1	1	0	0	-0.64	9.20E-05	-1.84	1.09E-24		Class III chitinase
HMPREF1120_06669	1	3	3	2	3	2	1	1	1	1	0	0.57	9.88E-04	0.41	7.23E-03	ChiB	Class V chitinase
HMPREF1120_03714	1	1	2	2	0	0	1	1	1	0	0	0.14	4.60E-01	0.48	9.28E-04		Class V chitinase
HMPREF1120_04557	2	0	0	0	0	0	0	0	0	0	0	0.73	3.60E-03	0.57	2.22E-04		Chitinase
HMPREF1120_07241	paralog											-2.9	1.56E-68	-0.93	9.28E-12		Chitinase
HMPREF1120_06035	2	2	1	1	2	2	1	1	1	0	0	-0.17	3.73E-01	0.25	1.06E-01	NagA	Extracellular N-acetyl-beta-glucosaminidase with a
HMPREF1120_06285	paralog											-0.04	8.30E-01	-0.49	2.87E-04	NagA	predicted role in chitin hydrolysis
AN4686	0	1	3	1	1	1	1	1	1	0	0					CsnA	Chitosanases (GH 75); similarity with A. fumigatus CsnC
AN11051	0	1	1	1	0	0	0	0	0	0	0					CsnC	and *A. oryzae* CsnC
**1,3-α-glucan synthesis and processing**																	
AN3307, AN5885	0	5	3	2	1	0	2	1	1	5	0					AgsB/A	Catalytic subunits of the 1,3-α-glucan synthase complex (GT5 and GH13)
AN3790,AN7349,AN9042	0	1	1	1	1	0	0	0	0	0	0					AgnB/C/D	Putative 1,3-α-glucanase family (GH 71); related to the S.
AN1604	0	2	3	1	0	0	1	1	1	0	0					AgnE	pombe Agn1-family
AN3308, AN4507,	0	2	3	3	1	0	1	1	0	0	0					AmyC/D	Amylase-like family (GH 13); similarity with A. fumigatus AmyA
HMPREF1120_08319	1	4	2	3	0	0	1	1	1	0	6	-0.03	8.84E-01	1.35	1.56E-24		
HMPREF1120_03460	1	1	1	1	1	1	2	1	0	0	0	-1.33	2.41E-16	1.74	1.85E-33		Putative amylase; similarity with H. capsulatum Amy1
**1,3-β-glucan synthesis and processing**																	
HMPREF1120_03476	1	1	1	1	1	1	1	1	1	1	4	1.03	3.01E-11	0.09	5.32E-01	FksA	Putative catalytic subunit 1,3-β-glucan synthase complex; ScFks1-like
HMPREF1120_04893	1	1	1	1	1	1	1	1	1	1	1	0.67	3.49E-05	0.95	1.94E-12	ScSMI1	Putative regulatory component 1,3-β-glucan synthesis; ScKnr4-like
HMPREF1120_09022	1	1	1	1	1	1	1	1	1	1	1	-0.14	1.34E-18	-1.14	2.92E-18	EngA	Endo-1,3-β-glucanase (GH 81-family); ScEng1-like
HMPREF1120_04506	1	1	1	1	0	1	0	0	0	1	1	-0.85	1.60E-06	-2.72	9.07E-95		Putative exo-1,3-β-glucanase family (GH 5); related to the
HMPREF1120_06180	1	1	1	1	1	1	1	1	1	0	1	-0.23	1.91E-01	0.01	9.74E-01		ScExg1-family
HMPREF1120_01556	2	1	3	1	1	1	4	4	4	0	0	1.23	4.73E-10	0.17	3.61E-01		Putative exo-1,3-β-glucanase family (GH 55); related to
HMPREF1120_05230	paralog											1.23	6.03E-08	-0.27	1.02E-01		Coniothyrium minitans exo-1,3-glucanase (Cmg1)
HMPREF1120_00547	2	1	1	1	0	0	2	1	1	0	0	3.14	9.48E-83	1.03	8.29E-16		Bgl2-family of putative 1,3-β-transglucosylases (GH 17) proposed to be involved in connecting the emerging 1,3-β-glucan chains to the existing b-glucan network through 1,6-β-linkages; related to A. fumigatus Bgt1-family
HMPREF1120_05209	paralog											1.68	2.06E-27	-0.5	1.29E-04		
HMPREF1120_06595	1	1	1	1	1	1	1	0	0	1	0	-1.88	4.33E-25	-1.51	4.66E-26		
HMPREF1120_08449	1	1	1	1	1	1	1	1	1	0	0	0.36	3.91E-02	-0.18	2.26E-01		
HMPREF1120_04141	1	1	1	1	1	1	0	0	0	1	1	-0.22	2.54E-01	0.79	2.77E-08		
HMPREF1120_03066	1	1	1	1	1	1	1	1	1	1	0	0.2	2.33E-01	-0.6	7.68E-06		
HMPREF1120_08078	1	0	0	0	0	0	0	0	0	0	0	6.64	1.85E-268	0.13	3.95E-01		
HMPREF1120_04931	1	1	1	1	1	1	1	1	1	1	0	2.73	2.65E-65	0.27	4.85E-02		Crh1-family of putative transglycosidases (GH 16);involved in crosslinking b-glucan and chitin; related to ScCrh-family
HMPREF1120_00627	1	1	1	1	1	1	1	1	1	0	0	0.08	6.89E-01	-0.62	9.59E-04		
HMPREF1120_07927	1	1	1	1	1	0	1	1	1	1	0	-0.42	1.77E-02	0.005	9.97E-01		
HMPREF1120_02703	1	1	1	0	1	1	1	1	1	1	2	0.09	6.58E-01	-0.05	7.77E-01		
HMPREF1120_07283	1	1	1	1	1	1	1	1	1	1	0	0.77	1.07E-06	0.08	5.72E-01		Gas-family of putative 1,3-β-transglucosylases (GH 72) proposed to be involved in connecting the emerging 1,3-β-glucan chains to the existing β-glucan network; related to A. fumigatus Gel-family
HMPREF1120_01763	1	1	1	1	1	1	2	2	2	1	2	1.96	3.15E-36	-0.02	9.28E-01		
HMPREF1120_03477	1	2	2	2	1	1	1	1	1	1	1	1.33	1.06E-17	0.11	4.56E-01		
HMPREF1120_01682	1	1	1	0	1	0	1	0	0	0	0	0.63	8.79E-05	0.28	3.88E-02	GelG	1,3-β-glucanosyltransferase
HMPREF1120_01649	1	1	1	1	1	1	1	1	1	1	2	0.63	7.07E-05	-0.48	4.40E-04	SunA	Sun family, involved in septation, possibly β-glucosidase
HMPREF1120_06902	1	1	1	1	1	1	1	1	1	1	1	0.92	1.48E-07	-0.16	4.07E-01	SunB	activity; related to ScSun-family
HMPREF1120_01614	1	1	1	1	1	1	0	0	0	1	2	1.5	1.52E-21	-0.55	2.19E-05	Kre6	Putative transglycosidase required for 1,6-β-glucan biosynthesis
HMPREF1120_04699	2	1	2	1	0	0	0	0	0	0	0	0.94	1.75E-09	0.47	3.56E-04	CelA	Similarity with cellulose synthases of the GT 2 family.
HMPREF1120_05299	paralog											-0.8	4.87E-07	1.8	6.57E-46	CelA	Putatively involved in 1,3-β-/1,4-β-glucan synthesis
HMPREF1120_02373	2	1	1	1	2	2	1	2	2	0	1	-1.55	1.03E-20	-0.65	8.03E-05	Mlg1	Mixed-linked glucanases in C. carbonum, hydrolyze 1,3-β-/1,4-β-glucans
HMPREF1120_07765	paralog											1.4	7.25E-19	-1.72	2.22E-40	Mlg1	
HMPREF1120_09051	1	2	1	1	0	0	0	1	1	0	0	3.95	8.92E-122	0.22	1.62E-01	Mlg1	
**Other cell wall biosynthesis proteins**																	
HMPREF1120_04431	1	1	1	1	1	1	1	1	1	0	0	1.11	4.02E-12	0.09	5.54E-01		
HMPREF1120_03513	1	1	1	1	0	0	1	1	1	0	0	0.19	3.28E-01	-0.08	6.40E-01		Endo-mannanase family (GH 76) with a putative role in
HMPREF1120_05522	1	2	2	1	2	2	2	2	1	1	1	2.67	1.53E-58	-0.19	1.82E-01		GPI-CWP incorporation; related to *S. cerevisiae* Dfg5
AN0393 (dfgC), AN3049 (dfgD), AN8421 (dfgB) AN0383 (dfgE)																	
HMPREF1120_03851	1	1	1	1	1	1	1	1	1	2	2	1.73	8.73E-29	-0.06	7.18E-01		Putative enzyme involved in cell wall biosynthesis with unknown function
HMPREF1120_08023	1	1	1	1	1	1	2	2	2	2	0	0.21	2.21E-01	-0.4	4.07E-03		Putative chitin deacetylases; similarity with ScCda1 and ScCda2

^ OrthoMCL clusters were identified based on the gene list in de Groot 2009, Fungal Genetics and Biology. For the GH18 family, we show only those involved in cell wall repair, which are listed by OrthoMCL cluster

***** *W. dermatitidis* gene IDs are listed for each OrthoMCL cluster. If a cluster does not have a *W. dermatitidis* gene, then *A. nidulans* gene IDs are listed.

****** $\log_2$Fold Change for pH is of pH2.5 compared to pH6, and $\log_2$Fold for radiation is of radiation treatment compared to untreated cells. Red indicates significant up-regulation under stress conditions (pH2.5 or with radiation) (corrected P-value < 1E-10), and blue indicates significant down-regulation under stress (corrected P-value < 1E-10).

******* P-values are corrected by the Benjamini and Hochberg method.

Gene symbols and descriptions are from de Groot 2009, Fungal Genetics and Biology.

& Split into separate rows, as they were named separately in de Groot 2009, although OrthoMCL placed Chs1 and Chs2 in the same ortholog cluster.
paralog indicates that the counts for the *W. dermatitidis* gene listed is absent as it is the paralog of the gene listed above it.

Table S3 Pathways involved in cell wall stress response and pH signaling.

Gene name	*A. nidulans*	*W. dermatitidis*	pH		Radiation			*A. niger* pH2.5 Vs pH6		
			Log_2Fold	P-value#	Log_2Fold	P-value#	Description	Gene ID	Log_2Fold	P-value
**Cell wall integrity pathway**										
Mid2	AN4897	HMPREF1120_03784	**1.48**	**2.04E-21**	0.55	3.01E-05	Transmembrane sensor for cell wall integrity signaling	An02g06660	-0.47	9.44E-02
Wsc1-3	AN6927	HMPREF1120_06587	**1.91**	**1.03E-33**	-0.57	1.84E-05	Sensor-transducer of the stress-activated PKC1-MPK1 kinase pathway	An01g09460<br>An02g13710	0.31<br>-0.25	3.51E-01<br>4.04E-01
	AN5660	HMPREF1120_05003	**1.5**	**3.32E-21**	0.48	3.00E-04		An03g00250<br>An07g04070	-0.10<br>0.31	6.54E-01<br>3.51E-01
	AN4674									
Rom2	AN4719	HMPREF1120_04966	-0.28	9.43E-02	-0.02	8.97E-01	GDP/GTP exchange factor (GEF)	An07g05090	0.41	8.59E-02
Rho1	AN5740	HMPREF1120_04193	0.48	2.63E-03	0.02	9.24E-01	Rho family GTPase	An18g05980	-0.32	1.35E-01
Rgd1	AN4745	HMPREF1120_02725	0.53	1.02E-03	0.19	1.98E-01	GTPase-activating protein	An11g10060	0.03	9.16E-01
Pkc1	AN0106	HMPREF1120_07353	0.34	3.97E-02	0.02	9.10E-01	Protein kinase C	An18g02400	-0.38	8.30E-02
Bkc1	AN4887	HMPREF1120_03472	0.37	2.49E-02	0.07	6.58E-01	mitogen-activated protein kinase kinase kinase (MAPKKK)	An02g06830	0.15	6.09E-01
Mkk1	AN4189	HMPREF1120_00691	**1.03**	**2.37E-10**	-0.1	4.94E-01	MAP kinase kinase (MAPKK)	An18g03740	0.24	2.43E-01
Mpk1	AN5666	HMPREF1120_06473	**2.32**	**4.15E-48**	0.09	5.59E-01	Serine/threonine MAP kinase	An01g09520	0.06	8.88E-01
Rlm1	AN2984	HMPREF1120_02025	**1.65**	**4.06E-25**	-0.35	1.19E-02	MADS-box transcription factor	An02g12210	-0.02	9.47E-01
Pst1	AN4390	HMPREF1120_03851	**1.73**	**8.73E-29**	-0.06	7.18E-01	GPI-anchored cell wall organization protein	An04g01230		
**UDP-GlcNAc synthesis pathway, required for chitin synthesis, GPI anchor biosynthesis and N-chain formation**										
gfaA	AN10709	HMPREF1120_00602	0.56	4.21E-04	-0.36	6.22E-03	Glutamine-fructose-6-phosphate transaminase (1st step)	An18g06820	2.16	2.63E-05
gnaA	AN8706	HMPREF1120_05484	0.5	2.43E-03	-0.19	2.20E-01	Glucosamine-phosphate N-acetyltransferase (2nd step)	An12g07840	0.39	3.14E-01
pcmA	AN4234	HMPREF1120_02062	0.3	7.16E-02	**-0.89**	**1.79E-11**	Phosphoacetylglucosamine mutase (3rd step)	An18g05160<br>An18g05170		
ungA	AN9094	HMPREF1120_05326	-0.03	8.59E-01	0.18	1.94E-01	UDP-N-acetylglucosamine pyrophosphorylase (4th step)	An12g00480	0.06	8.70E-01
**HOG signaling pathway**										
SLN1	AN1800	HMPREF1120_04358	0.78	8.33E-07	**0.85**	**3.84E-11**	Histidine kinase osmosensor that regulates a MAP kinase cascade			
YPD1	AN2005	HMPREF1120_08241	0.13	4.60E-01	0.4	3.41E-03	Phosphorelay intermediate protein	An04g06570	0.36	2.02E-01
SSK1	AN7697	HMPREF1120_04973	0.35	3.62E-02	0.37	5.95E-03	Cytoplasmic response regulator	An03g04670	0.04	8.48E-01
SSK2	AN10153	HMPREF1120_04310	0.43	8.79E-03	-0.33	1.70E-02	MAP kinase kinase kinase of the HOG1 signaling pathway	An08g03240	-0.05	8.99E-01
PBS2	AN0931	HMPREF1120_02538	0.52	1.13E-03	0.15	3.18E-01	MAP kinase kinase of the HOG signaling pathway	An01g11080	0.04	8.94E-01
HOG1	AN1017	HMPREF1120_05833	0.03	8.92E-01	0.08	6.11E-01	Mitogen-activated protein kinase involved in osmoregulation	An08g05850	-0.14	5.70E-01
**Ca2+/calcineurin signaling pathway**										
CCH1	AN1168	HMPREF1120_08350	**2.91**	**6.25E-72**	-0.7	5.25E-08	Voltage-gated high-affinity calcium channel	An08g03400	-0.23	2.92E-01
MID1	AN8842	HMPREF1120_05098	0.25	1.48E-01	0.21	1.44E-01	N-glycosylated integral membrane protein of the ER and plasma membrane	An17g02075	-0.12	5.46E-01
CMD1	AN2047	HMPREF1120_06249	-0.04	8.47E-01	0.36	7.07E-03	Calmodulin	An04g07010<br>An07g01640	0.44<br>0.03	1.71E-01<br>9.52E-01
CNA1	AN8820	HMPREF1120_03111	0.39	1.81E-02	-0.84	1.34E-10	Calcineurin A	An07g03620	-0.31	3.01E-01
CNB1	AN6566	HMPREF1120_02693	-0.06	7.60E-01	0.33	2.35E-02	Calcineurin B	An15g00870	-0.26	5.00E-01
CRZ1	AN5726	HMPREF1120_03777	0.16	3.81E-01	0.1	5.26E-01	Transcription factor that activates genes involved in stress response	An18g05920	0.31	3.18E-01
**pH signaling pathway**										
PacC	AN2855	HMPREF1120_00699	-0.36	2.89E-02	0.04	8.08E-01	Transcription factor	An02g07890	-3.03	8.18E-05
PalA	AN4351	HMPREF1120_02658	-0.24	1.72E-01	0.42	2.90E-03		An04g00760	-0.01	9.89E-01
PalB	AN0256	HMPREF1120_07519	0.14	4.67E-01	-0.01	9.38E-01	cysteine protease	An01g04680	-0.33	2.25E-01
PalC	AN7560	HMPREF1120_08758	-0.17	3.63E-01	-5.05	7.75E-01		An15g03350	-0.33	3.62E-01
PalF	AN1844	HMPREF1120_00084	0.97	1.14E-09	0.5	1.28E-04		An04g07460		
PalH	AN6886	HMPREF1120_01470	0.19	2.68E-01	0.25	7.19E-02	plasma membrane pH sensor	An14g04650	-0.08	7.90E-01
PalI	AN4853	HMPREF1120_06064	0.72	6.13E-06	-0.18	2.15E-01	plasma membrane pH sensor	An02g13150	0.05	8.59E-01
Vsp32/Snf7	AN4240	HMPREF1120_03523	-0.3	9.04E-02	-0.03	8.50E-01		An18g05430	-0.04	8.96E-01
**Chitin synthase genes**										
CHS2		HMPREF1120_06816	**1.75**	**7.17E-28**	0.49	2.88E-04		An07g05570	-0.14	6.42E-01
CHS1		HMPREF1120_07981	0.38	2.19E-02	**-1.73**	**1.68E-24**		An14g00660	0.05	9.16E-01
CHS3		HMPREF1120_06479	**1.86**	**3.01E-32**	0.43	1.35E-03		An09g04010	-1.03	4.18E-03
								An12g10380	1.98	1.85E-04
								An03g06360	0.11	5.76E-01
CHS4		HMPREF1120_07721	**1.32**	**2.77E-17**	-0.23	9.57E-02		An09g02290	-0.46	5.45E-02
CHS5		HMPREF1120_08776	**1.31**	**3.54E-17**	-0.68	1.18E-07		An02g02340	-0.28	4.16E-01
CHS7		HMPREF1120_08777	**1.48**	**1.82E-21**	-0.65	3.85E-07		An02g02360		
CHS6		HMPREF1120_09115	0.04	8.82E-01	-0.34	1.22E-02		An08g05290	0.47	7.67E-02

# P-values are corrected by the Benjamini and Hochberg method.

Table S4 GSEA tests on gene sets defined by InterPro domain, KEGG pathway, COG, MFS and APC transporter, and cell wall and related pathway.

Gene set ID	Gene set description	Size	pH enrichment			Radiation enrichment		
			ES*	NES*	q-value#	ES*	NES*	q-value#
**InterPro domain (q-value < 0.05)**								
IPR001128	Cytochrome P450	65	0.565	2.012	4.269E-03	-0.592	-2.121	2.050E-04
IPR003819	Taurine catabolism dioxygenase TauD/TfdA	16	0.741	1.967	5.661E-03	-0.431	-1.159	6.033E-01
IPR001461	Peptidase A1	10	0.829	1.934	6.252E-03	-0.287	-0.686	9.728E-01
IPR016196	Major facilitator superfamily, general substrate transporter	318	0.463	1.955	6.563E-03	-0.336	-1.487	2.293E-01
IPR021109	Peptidase aspartic	11	0.819	1.976	6.748E-03	-0.486	-1.157	6.049E-01
IPR008972	Cupredoxin	16	0.715	1.919	6.925E-03	-0.424	-1.126	6.430E-01
IPR006593	Cytochrome b561/ferric reductase transmembrane	6	0.936	1.888	1.006E-02	-0.285	-0.576	9.868E-01
IPR011701	Major facilitator superfamily MFS-1	230	0.455	1.858	1.346E-02	-0.295	-1.253	5.108E-01
IPR020846	Major facilitator superfamily	238	0.435	1.791	3.120E-02	-0.342	-1.464	2.599E-01
IPR002293	Amino acid/polyamine transporter I	52	0.521	1.768	3.712E-02	-0.405	-1.375	3.996E-01
IPR011707	Multicopper oxidase, type 3	13	0.700	1.753	3.763E-02	-0.485	-1.228	5.463E-01
IPR016048	Luciferase-like, subgroup	8	0.783	1.755	3.788E-02	0.300	0.682	1.000E+00
IPR004841	Amino acid permease domain	50	0.517	1.762	3.878E-02	-0.382	-1.295	4.721E-01
IPR001117	Multicopper oxidase, type 1	13	0.700	1.759	3.910E-02	-0.485	-1.189	5.816E-01
IPR011706	Multicopper oxidase, type 2	13	0.700	1.755	3.949E-02	-0.476	-1.180	5.897E-01
IPR005828	General substrate transporter	66	0.490	1.729	4.885E-02	-0.488	-1.743	3.136E-02
IPR020946	Flavin-containing monooxygenase-like	27	0.581	1.719	5.322E-02	-0.637	-1.876	5.444E-03
IPR012132	Glucose-methanol-choline oxidoreductase	9	0.700	1.602	1.568E-01	-0.757	-1.711	4.319E-02
IPR007219	Transcription factor, fungi	146	0.383	1.511	2.594E-01	-0.418	-1.691	4.914E-02
IPR001752	Kinesin, motor domain	11	0.537	1.297	5.918E-01	-0.798	-1.905	3.565E-03
IPR008775	Phytanoyl-CoA dioxygenase	11	0.505	1.219	6.921E-01	-0.732	-1.723	3.895E-02
IPR000873	AMP-dependent synthetase/ligase	45	0.352	1.168	7.428E-01	-0.618	-2.041	5.110E-04
IPR020617	Thiolase, C-terminal	8	0.520	1.166	7.449E-01	-0.908	-1.939	2.245E-03
IPR002155	Thiolase	8	0.520	1.146	7.650E-01	-0.908	-1.940	2.299E-03
IPR020616	Thiolase, N-terminal	7	0.531	1.117	7.759E-01	-0.900	-1.891	4.522E-03
IPR006092	Acyl-CoA dehydrogenase, N-terminal	16	0.335	0.877	9.679E-01	-0.803	-2.098	2.520E-04
IPR009100	Acyl-CoA dehydrogenase/oxidase	19	0.318	0.863	9.786E-01	-0.816	-2.264	0.000E+00
IPR009075	Acyl-CoA dehydrogenase/oxidase C-terminal	19	0.318	0.858	9.825E-01	-0.816	-2.229	0.000E+00
IPR001753	Crotonase, core	16	0.201	0.538	1.000E+00	-0.785	-2.072	2.920E-04
IPR003395	RecF/RecN/SMC	7	0.312	0.667	1.000E+00	-0.845	-1.771	2.137E-02
IPR006090	Acyl-CoA oxidase/dehydrogenase, type 1	16	0.250	0.668	1.000E+00	-0.819	-2.154	0.000E+00
IPR006091	Acyl-CoA oxidase/dehydrogenase, central domain	17	0.253	0.673	1.000E+00	-0.823	-2.162	0.000E+00
IPR006139	D-isomer specific 2-hydroxyacid dehydrogenase, catalytic domain	9	0.297	0.674	1.000E+00	-0.809	-1.858	7.151E-03
IPR012000	Thiamine pyrophosphate enzyme, central domain	9	0.246	0.565	1.000E+00	-0.745	-1.698	4.716E-02
IPR012001	Thiamine pyrophosphate enzyme, N-terminal TPP-binding domain	9	0.246	0.551	1.000E+00	-0.745	-1.704	4.475E-02
IPR015830	Amidase, fungi	10	0.291	0.707	1.000E+00	-0.841	-1.908	3.433E-03
IPR001509	NAD-dependent epimerase/dehydratase	23	-0.642	-2.143	3.114E-03	0.348	1.044	8.678E-01
IPR001163	Like-Sm ribonucleoprotein (LSM) domain	16	-0.676	-2.057	6.299E-03	0.273	0.745	9.837E-01
IPR006195	Aminoacyl-tRNA synthetase, class II	17	-0.656	-2.027	1.016E-02	0.471	1.317	6.736E-01
IPR015424	Pyridoxal phosphate-dependent transferase, major domain	53	-0.491	-1.990	1.185E-02	-0.475	-1.621	9.369E-02
IPR009000	Translation elongation/initiation factor/Ribosomal, beta-barrel	19	-0.624	-1.994	1.198E-02	0.305	0.867	9.149E-01
IPR023210	NADP-dependent oxidoreductase domain	28	-0.563	-2.000	1.230E-02	0.469	1.524	4.446E-01
IPR016027	Nucleic acid-binding, OB-fold-like	44	-0.501	-1.994	1.268E-02	-0.243	-0.811	9.475E-01
IPR009080	Aminoacyl-tRNA synthetase, class 1a, anticodon-binding	10	-0.757	-1.964	1.348E-02	0.304	0.738	9.842E-01
IPR001395	Aldo/keto reductase	28	-0.560	-1.970	1.400E-02	0.508	1.622	3.016E-01
IPR006649	Like-Sm ribonucleoprotein (LSM) domain, eukaryotic/archaea-type	16	-0.676	-1.964	1.409E-02	0.273	0.763	9.759E-01
IPR002423	Chaperonin Cpn60/TCP-1	10	-0.724	-1.941	1.675E-02	0.390	0.955	8.640E-01
IPR004161	Translation elongation factor EFTu/EF1A, domain 2	13	-0.679	-1.913	2.253E-02	0.288	0.755	9.795E-01
IPR002917	GTP-binding protein, HSR1-related	11	-0.720	-1.910	2.290E-02	0.639	1.633	2.897E-01
IPR020568	Ribosomal protein S5 domain 2-type fold	29	-0.532	-1.906	2.295E-02	-0.204	-0.619	9.835E-01
IPR014014	RNA helicase, DEAD-box type, Q motif	23	-0.569	-1.886	2.785E-02	0.670	2.040	4.650E-03
IPR001353	Proteasome, subunit alpha/beta	14	-0.635	-1.860	3.594E-02	-0.418	-1.076	7.106E-01
IPR019781	WD40 repeat, subgroup	103	-0.393	-1.842	4.190E-02	0.233	0.965	8.687E-01
IPR015815	3-hydroxyacid dehydrogenase/reductase	10	-0.596	-1.535	2.884E-01	-0.780	-1.847	8.142E-03
IPR016161	Aldehyde/histidinol dehydrogenase	21	-0.449	-1.440	3.935E-01	-0.741	-2.040	4.810E-04
IPR015590	Aldehyde dehydrogenase domain	21	-0.413	-1.349	4.911E-01	-0.750	-2.103	2.750E-04
IPR008927	6-phosphogluconate dehydrogenase, C-terminal-like	31	-0.344	-1.220	5.993E-01	-0.594	-1.840	8.589E-03
IPR012338	Beta-lactamase-type transpeptidase fold	7	-0.489	-1.149	6.303E-01	-0.820	-1.727	3.812E-02

IPR000425	Major intrinsic protein	9	-0.390	-0.991	7.736E-01	0.874	2.078	3.904E-03
IPR000120	Amidase	20	-0.301	-0.982	7.798E-01	-0.640	-1.792	1.609E-02
IPR004136	2-nitropropane dioxygenase, NPD	5	-0.433	-0.884	8.545E-01	-0.907	-1.704	4.553E-02
IPR006140	D-isomer specific 2-hydroxyacid dehydrogenase, NAD-binding	13	-0.255	-0.717	9.610E-01	-0.675	-1.709	4.349E-02
**KEGG pathway (q-value < 0.05)**								
ko05012	Parkinson's disease	53	-0.592	-2.457	6.850E-04	0.137	0.505	1.000E+00
ko05010	Alzheimer's disease	53	-0.552	-2.242	1.367E-03	0.121	0.449	9.986E-01
ko05016	Huntington's disease	69	-0.505	-2.155	4.106E-03	0.260	1.005	8.700E-01
ko00970	Aminoacyl-tRNA biosynthesis	13	-0.734	-2.105	4.071E-03	0.234	0.599	9.952E-01
ko00240	Pyrimidine metabolism	36	-0.566	-2.088	5.397E-03	0.255	0.863	9.170E-01
ko00591	Linoleic acid metabolism	35	-0.556	-2.068	6.218E-03	0.371	1.277	7.197E-01
ko04260	Cardiac muscle contraction	12	-0.718	-2.009	1.233E-02	0.333	0.847	9.328E-01
ko03020	RNA polymerase	20	-0.634	-2.001	1.303E-02	0.662	1.962	1.722E-02
ko00650	Butanoate metabolism	72	-0.436	-1.918	2.177E-02	-0.411	-1.509	1.993E-01
ko00363	Bisphenol degradation	43	-0.490	-1.889	2.778E-02	0.333	1.185	7.491E-01
ko03420	Nucleotide excision repair	23	-0.564	-1.852	3.903E-02	-0.425	-1.213	5.602E-01
ko03040	Spliceosome	16	-0.608	-1.849	3.907E-02	0.319	0.906	8.917E-01
ko00360	Phenylalanine metabolism	26	-0.350	-1.211	5.885E-01	-0.772	-2.286	0.000E+00
ko00380	Tryptophan metabolism	26	-0.264	-0.914	8.342E-01	-0.752	-2.264	0.000E+00
ko00280	Valine, leucine and isoleucine degradation	27	0.237	0.709	1.000E+00	-0.744	-2.222	0.000E+00
ko00627	Aminobenzoate degradation	45	0.371	1.214	6.982E-01	-0.647	-2.159	0.000E+00
ko00350	Tyrosine metabolism	34	-0.262	-0.963	7.876E-01	-0.675	-2.082	2.330E-04
ko00410	beta-Alanine metabolism	27	-0.257	-0.905	8.393E-01	-0.699	-2.054	4.760E-04
ko00960	Tropane, piperidine and pyridine alkaloid biosynthesis	17	0.369	0.994	8.877E-01	-0.741	-1.989	1.018E-03
ko00626	Naphthalene degradation	45	0.340	1.125	7.765E-01	-0.595	-1.971	1.441E-03
ko00643	Styrene degradation	10	-0.387	-1.007	7.595E-01	-0.818	-1.948	2.291E-03
ko00281	Geraniol degradation	12	0.454	1.126	7.803E-01	-0.805	-1.947	2.231E-03
ko00362	Benzoate degradation	25	0.206	0.609	1.000E+00	-0.660	-1.946	2.220E-03
ko00950	Isoquinoline alkaloid biosynthesis	15	0.284	0.735	1.000E+00	-0.738	-1.909	3.565E-03
ko00310	Lysine degradation	18	-0.502	-1.554	2.785E-01	-0.695	-1.887	4.812E-03
ko00903	Limonene and pinene degradation	42	0.274	0.899	9.647E-01	-0.576	-1.872	5.913E-03
ko03320	PPAR signaling pathway	14	0.387	0.977	8.833E-01	-0.712	-1.833	9.350E-03
ko04113	Meiosis - yeast	35	-0.164	-0.594	9.812E-01	-0.570	-1.807	1.346E-02
ko00071	Fatty acid metabolism	9	0.621	1.417	3.881E-01	-0.794	-1.804	1.373E-02
ko00940	Phenylpropanoid biosynthesis	10	0.617	1.453	3.429E-01	-0.756	-1.727	3.885E-02
ko00640	Propanoate metabolism	32	0.225	0.689	1.000E+00	-0.558	-1.720	3.969E-02
ko04146	Peroxisome	23	0.419	1.197	7.181E-01	-0.577	-1.694	4.915E-02
ko03030	DNA replication	9	-0.660	-1.648	1.610E-01	-0.732	-1.694	4.851E-02
**COG category (q-value < 0.05)**								
COGJ	Translation, ribosomal structure and biogenesis	319	-0.510	-2.731	0.000E+00	0.245	1.190	7.557E-01
COGE	Amino acid transport and metabolism	447	-0.245	-1.329	5.086E-01	-0.426	-1.927	2.675E-03
COGZ	Cytoskeleton	50	0.236	0.802	1.000E+00	-0.540	-1.853	7.153E-03
**MFS and APC transporter (see Figure S1)**								
MFS core	MFS core gene	100	0.376	1.402	4.082E-01	-0.406	-1.54	1.637E-01
MFS shared	MFS share gene	102	0.514	1.95	6.208E-03	-0.401	-1.538	1.646E-01
MFS uniq	MFS uniq gene	51	0.535	1.82	2.033E-02	-0.38	-1.302	4.716E-01
MFS_SP core	MFS_SP core gene	21	0.485	1.374	4.409E-01	-0.569	-1.581	1.307E-01
MFS_SP shared	MFS_SP share gene	24	0.492	1.453	3.346E-01	-0.628	-1.857	7.153E-03
MFS_SP uniq	MFS_SP uniq gene	12	0.577	1.407	3.974E-01	-0.426	-1.046	7.352E-01
MFS_DHA1 core	MFS_DHA1 core gene	22	-0.335	-1.121	6.480E-01	0.241	0.714	9.977E-01
MFS_DHA1 shared	MFS_DHA1 share gene	24	0.472	1.391	4.100E-01	0.317	0.982	8.761E-01
MFS_DHA1 uniq	MFS_DHA1 uniq gene	6	0.46	0.922	9.365E-01	-0.427	-0.853	9.244E-01
MFS_ACS core	MFS_ACS core gene	16	0.611	1.64	1.071E-01	-0.565	-1.507	2.017E-01
MFS_ACS shared	MFS_ACS share gene	38	0.657	2.09	7.790E-04	-0.483	-1.548	1.638E-01
MFS_ACS uniq	MFS_ACS uniq gene	21	0.639	1.796	2.564E-02	-0.439	-1.236	5.342E-01
APC core	APC core gene	21	0.434	1.214	6.645E-01	-0.351	-0.992	8.025E-01
APC shared	APC share gene	14	0.642	1.647	1.035E-01	-0.514	-1.314	4.684E-01
APC uniq	APC uniq gene	17	0.582	1.541	2.216E-01	-0.549	-1.491	2.150E-01
APC_ACT core	APC_ACT core gene	6	0.499	1.003	8.630E-01	-0.567	-1.11	6.563E-01
APC_ACT shared	APC_ACT share gene	8	0.593	1.307	5.395E-01	-0.329	-0.707	9.716E-01
APC_ACT uniq	APC_ACT uniq gene	7	0.798	1.664	8.943E-02	-0.563	-1.196	5.801E-01
APC_YAT core	APC_YAT core gene	12	0.397	0.972	8.851E-01	-0.358	-0.893	8.908E-01
APC_YAT shared	APC_YAT share gene	5	0.746	1.401	3.971E-01	-0.681	-1.317	4.653E-01
APC_YAT uniq	APC_YAT uniq gene	10	0.483	1.134	7.469E-01	-0.543	-1.23	5.431E-01
**Cell wall and pathway gene (see Tables 1, S2 and S3)**								
Chitin_syn	Chitin synthase	7	0.876	1.872	1.081E-02	-0.528	-1.115	6.539E-01
Chitin_syn_reg	Chitin synthesis regulation	8	0.674	1.454	3.348E-01	-0.301	-0.65	9.806E-01
Chitin_degrad	Chitin degradation	8	-0.465	-1.132	6.377E-01	-0.519	-1.136	6.286E-01

Glucan_beta_pro	Glucan 1,3-beta processing	27	0.541	1.595	1.548E-01	-0.388	-1.159	6.096E-01
Melanin_biosyn	Melanin biosynthesis	21	0.658	1.836	1.672E-02	-0.352	-0.997	7.962E-01
CWI	Cell wall integrity pathway	12	0.731	1.813	2.152E-02	-0.325	-0.801	9.490E-01
HOG	HOG pathway	6	0.592	1.202	6.876E-01	0.516	1.089	8.472E-01
Calcineurin	Calcineurin pathway genes	6	0.622	1.23	6.533E-01	-0.517	-1.036	7.422E-01
UDP-GlcNAc_syn	UDP-GlcNAc synthesis pathway	4	0.659	1.188	6.989E-01	-0.557	-1.004	7.897E-01
pH_sig	pH signaling pathway	8	0.416	0.902	9.446E-01	0.423	0.961	8.749E-01
Motor_prot	Motor protein	17	0.618	1.699	6.160E-02	-0.704	-1.882	5.022E-03
Calcium_trp	Calcium transporter	15	0.698	1.833	1.660E-02	0.457	1.259	7.203E-01
Light_sensing	Light sensing gene	11	0.365	0.871	9.617E-01	0.378	0.95	8.688E-01

***** Enrichment scores (ES) and normalized enrichment scores (NES) that are greater than 0 indicates the gene families are enriched under stress conditions (low pH or with radiation); negative ES and NES scores indicates enrichment under normal conditions (pH 6 or without radiation).

Significant enrichments (q-value < 0.05) are highlighted in blue (pH experiment) and red (radiation experiment).
