# Supplementary material for: Comparative Genomic and Transcriptomic Analysis of Wangiella dermatitidis, A Major Cause of Phaeohyphomycosis and a Model Black Yeast Human Pathogen
Source: G3 (Bethesda). 2014 Feb 4;4(4):561–78. doi: 10.1534/g3.113.009241 (PMC4059230; doi:10.1534/g3.113.009241)
Supplement: Supporting Information [file supp_g3.113.009241_FigureS1.pdf]

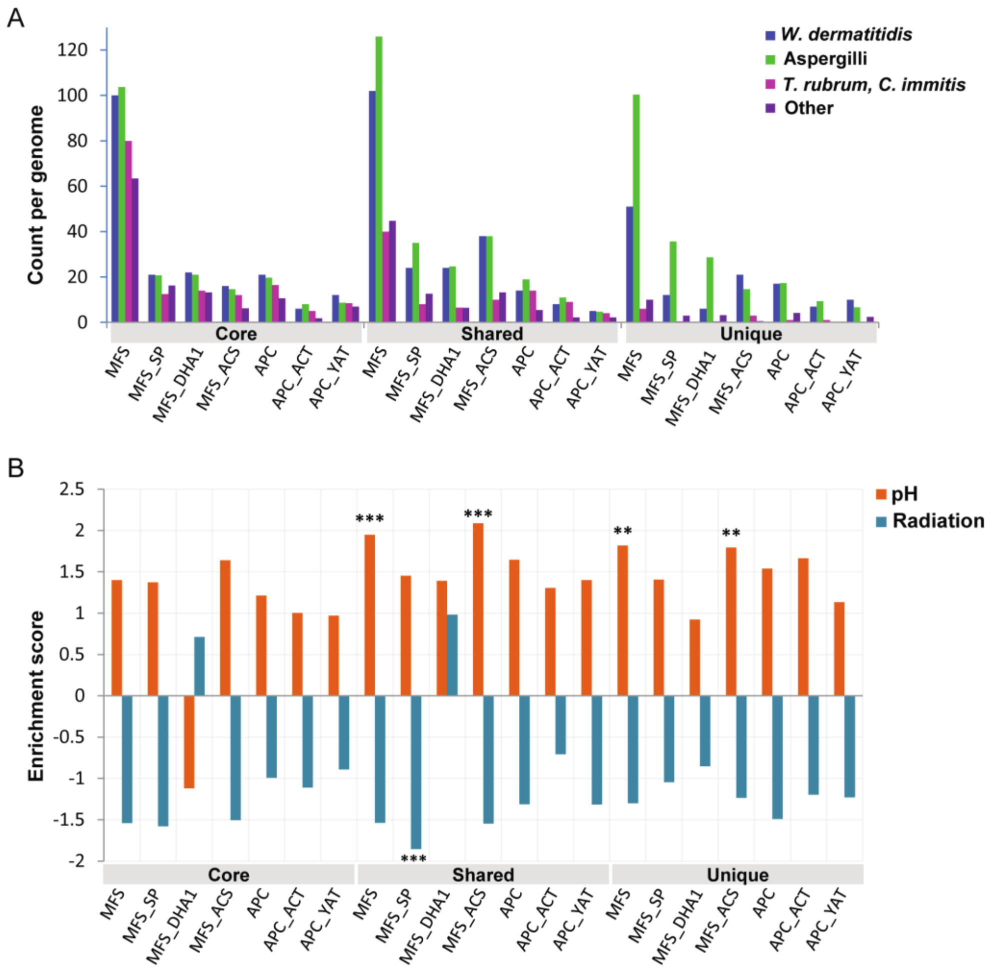

Figure S1 Independent expansion of MFS and APC transporter families in *W. dermatitidis* and selected aspergilli. (**A**) Average number of genes per genome for different category of MFS and APC families (Core families are the ortholog clusters shared by all four fungal groups; Shared, present in at least two out of the four fungal groups; Unique, unique to each group, including species-specific paralogous clusters and unclustered genes). (**B**) Enrichment of different category of MFS and APC transporters under different stress conditions (low pH or radiation). A positive normalized enrichment score (NES) indicates enrichment under stress conditions (pH 2.5 or with radiation), and a negative score indicates enrichment under normal conditions (pH 6 or no radiation). Significant enrichments noted with **: q-value < 0.05; ***: q-value < 0.01.
