# Supplementary material for: Comparative Genomic and Transcriptomic Analysis of Wangiella dermatitidis, A Major Cause of Phaeohyphomycosis and a Model Black Yeast Human Pathogen
Source: G3 (Bethesda). 2014 Feb 4;4(4):561–78. doi: 10.1534/g3.113.009241 (PMC4059230; doi:10.1534/g3.113.009241)
Supplement: Supporting Information [file supp_g3.113.009241_FigureS2.pdf]

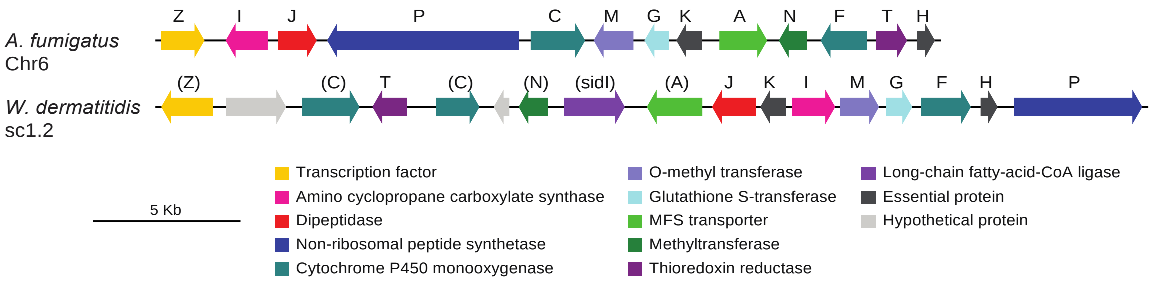

Figure S2 Gliotoxin biosynthetic gene cluster in *A. fumigatus* and conservation in *W. dermatitidis*. Gene symbols above each gene indicate the corresponding orthologs; symbols in parentheses were not identified as single copy orthologs of the corresponding *A. fumigatus* genes, but are part of the gene cluster and share sequence similarity. Gene identifiers for *W. dermatitidis* range from HMPREF1120_02977 (GliZ) to HMPREF1120_02933 (GliP).
