# Supplementary material for: Comparative Genomic and Transcriptomic Analysis of Wangiella dermatitidis, A Major Cause of Phaeohyphomycosis and a Model Black Yeast Human Pathogen
Source: G3 (Bethesda). 2014 Feb 4;4(4):561–78. doi: 10.1534/g3.113.009241 (PMC4059230; doi:10.1534/g3.113.009241)
Supplement: Supporting Information [file supp_g3.113.009241_FigureS3.pdf]

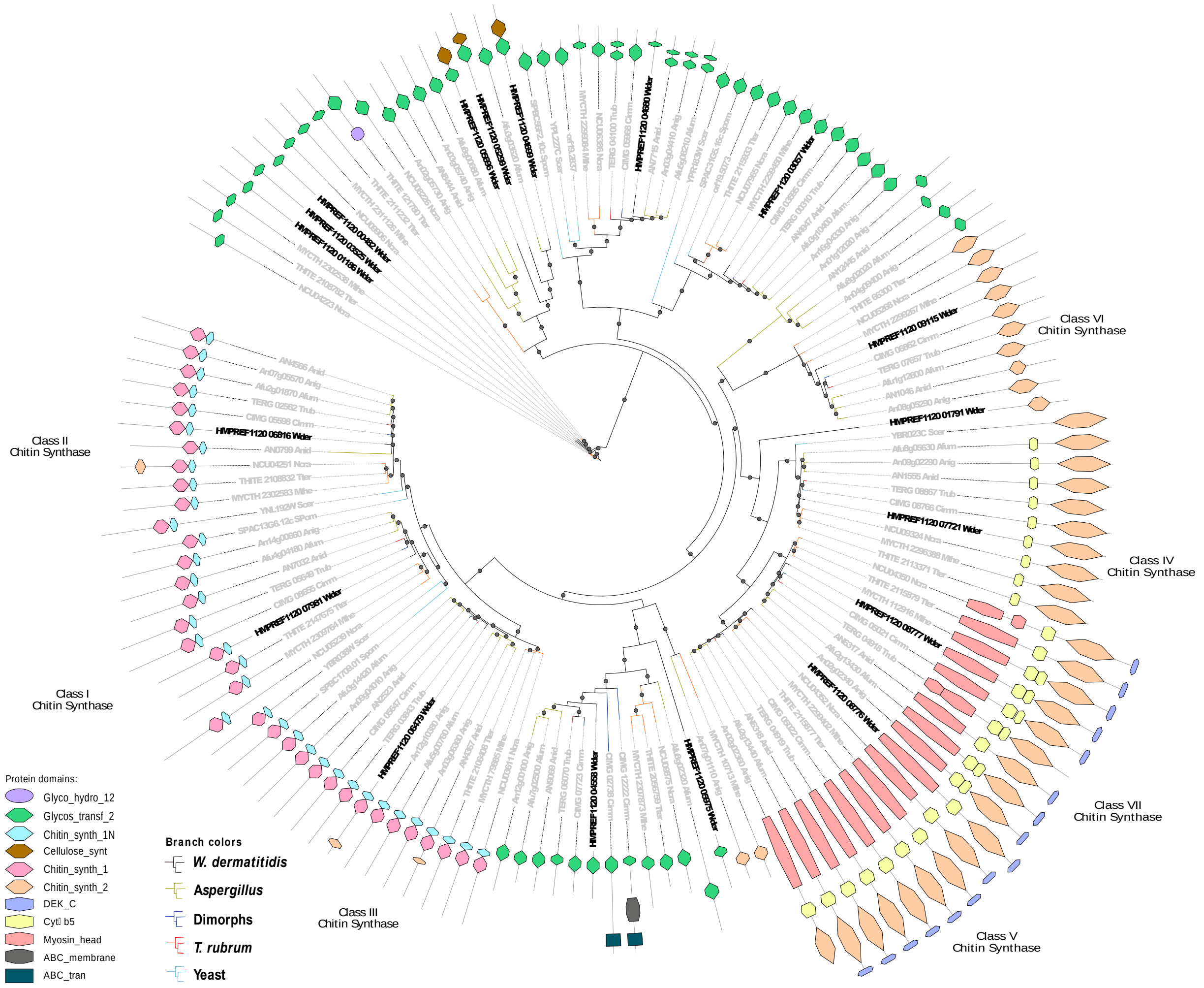

Figure S3 GT2 and chitin synthase tree. Phylogenetic tree of chitin synthase genes and other genes with the GT2 domain. Branch colors indicate species, where black denotes *W. dermatitidis* (bold text labels), blue *C. immitis*, yellow aspergilli, orange Pezizomycotina (*N. crassa*, *M. thermophilia*, *T. terrestris*) and red *T. rubrum*. The phylogeny was estimated with RAxML with the PROTGAMMAWAGF model and 1,000 bootstrap replicates. Nodes with a dot are supported by at least 75% of bootstrap replicates.
