# Supplementary material for: Comparative Genomic and Transcriptomic Analysis of Wangiella dermatitidis, A Major Cause of Phaeohyphomycosis and a Model Black Yeast Human Pathogen
Source: G3 (Bethesda). 2014 Feb 4;4(4):561–78. doi: 10.1534/g3.113.009241 (PMC4059230; doi:10.1534/g3.113.009241)
Supplement: Supporting Information [file supp_g3.113.009241_FigureS4.pdf]

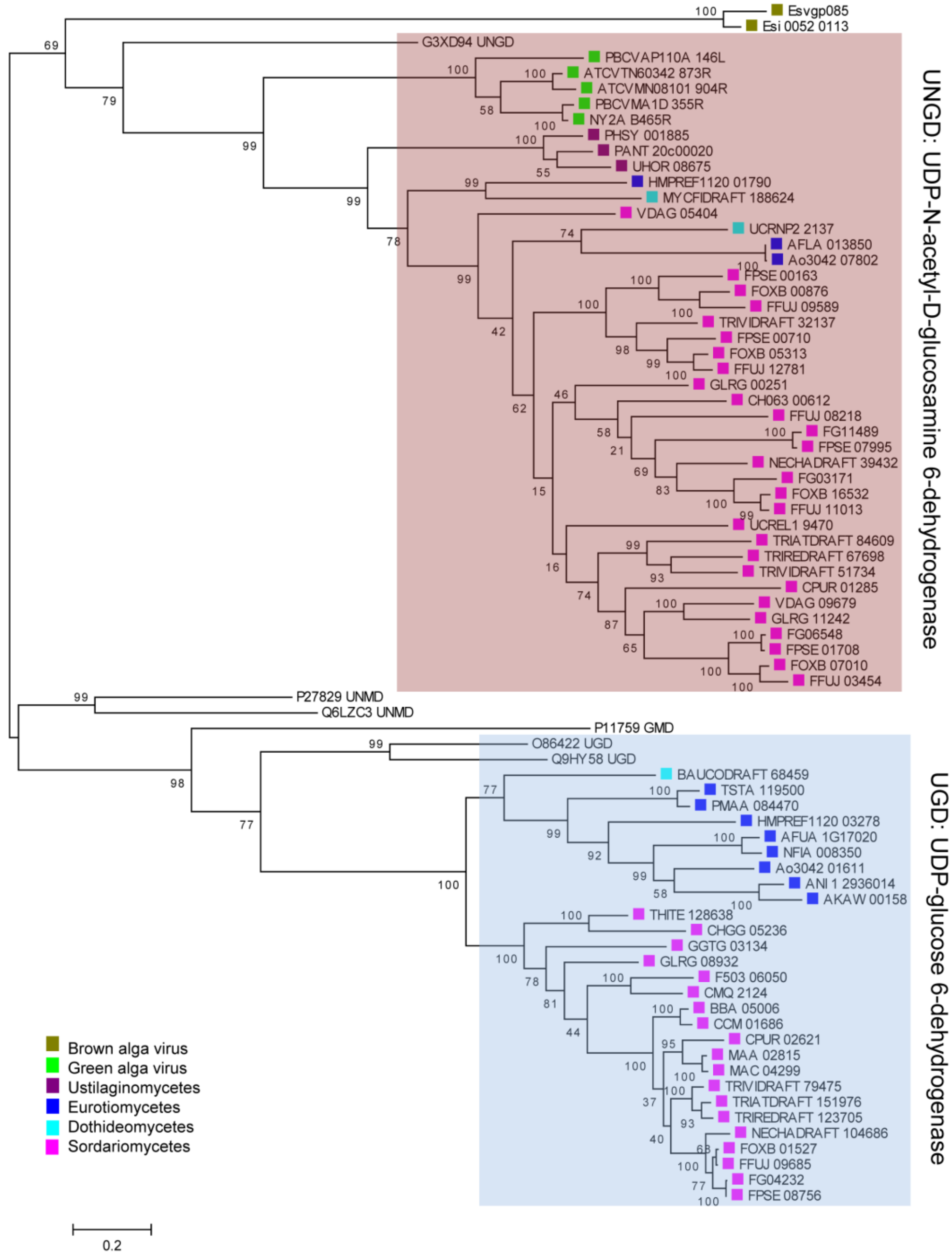

Figure S4 Classification of nucleotide sugar dehydrogenases. A phylogeny was inferred using maximum likelihood with MEGA5, performing 1,000 bootstrap replicates, for UDP-N-acetylglucosamine 6-dehydrogenases (UNGD), UDP-glucose 6-dehydrogenases (UGD), and several experimentally characterized bacterial nucleotide sugar dehydrogenases.
