# Supplementary material for: Comparative Genomic and Transcriptomic Analysis of Wangiella dermatitidis, A Major Cause of Phaeohyphomycosis and a Model Black Yeast Human Pathogen
Source: G3 (Bethesda). 2014 Feb 4;4(4):561–78. doi: 10.1534/g3.113.009241 (PMC4059230; doi:10.1534/g3.113.009241)
Supplement: Supporting Information [file supp_g3.113.009241_FigureS5.pdf]

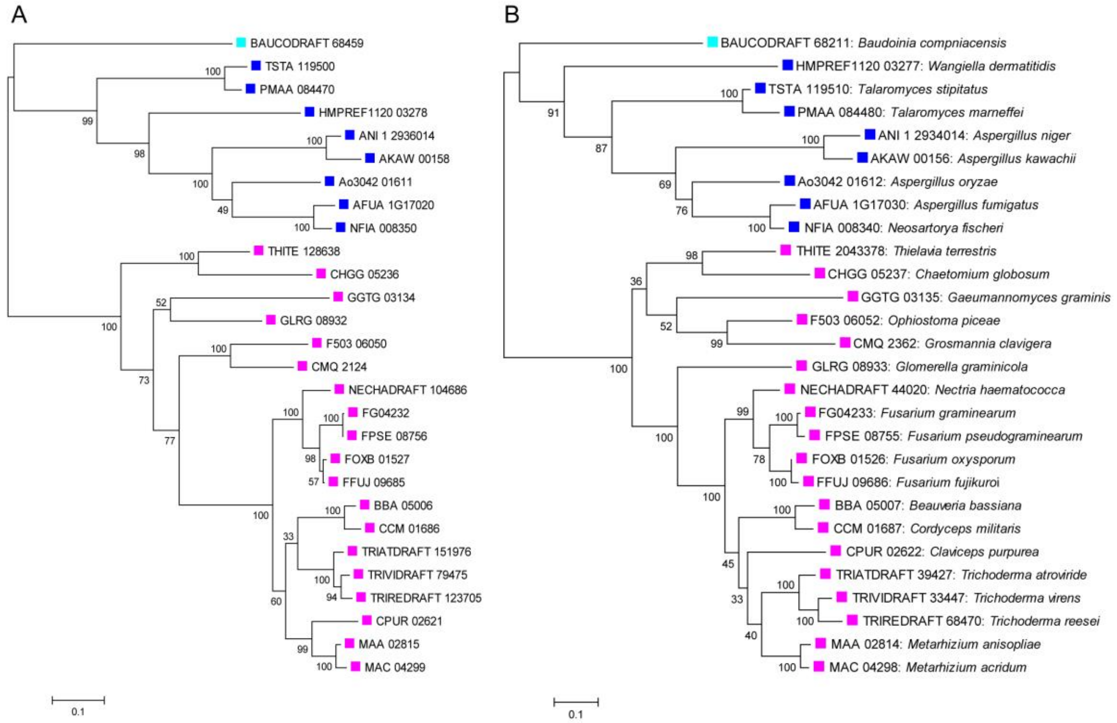

Figure S5 Phylogenies of UDP-glucose 6-dehydrogenases (**A**) and glycosyl transferase family 1 (**B**). The trees were inferred using maximum likelihood with MEGA5, performing 1,000 bootstrap replicates. Pairs of genes from each panel (**A, B**) are adjacent each fungal genome. The colored box icons indicate species groups as follows: Cyan: Dothideomycetes (Doth); Blue: Eurotiomycetes (Euro); Pink: Sordariomycetes (Sord).
