# Supplementary material for: Comparative Genomic and Transcriptomic Analysis of Wangiella dermatitidis, A Major Cause of Phaeohyphomycosis and a Model Black Yeast Human Pathogen
Source: G3 (Bethesda). 2014 Feb 4;4(4):561–78. doi: 10.1534/g3.113.009241 (PMC4059230; doi:10.1534/g3.113.009241)
Supplement: Supporting Information [file supp_g3.113.009241_FigureS6.pdf]

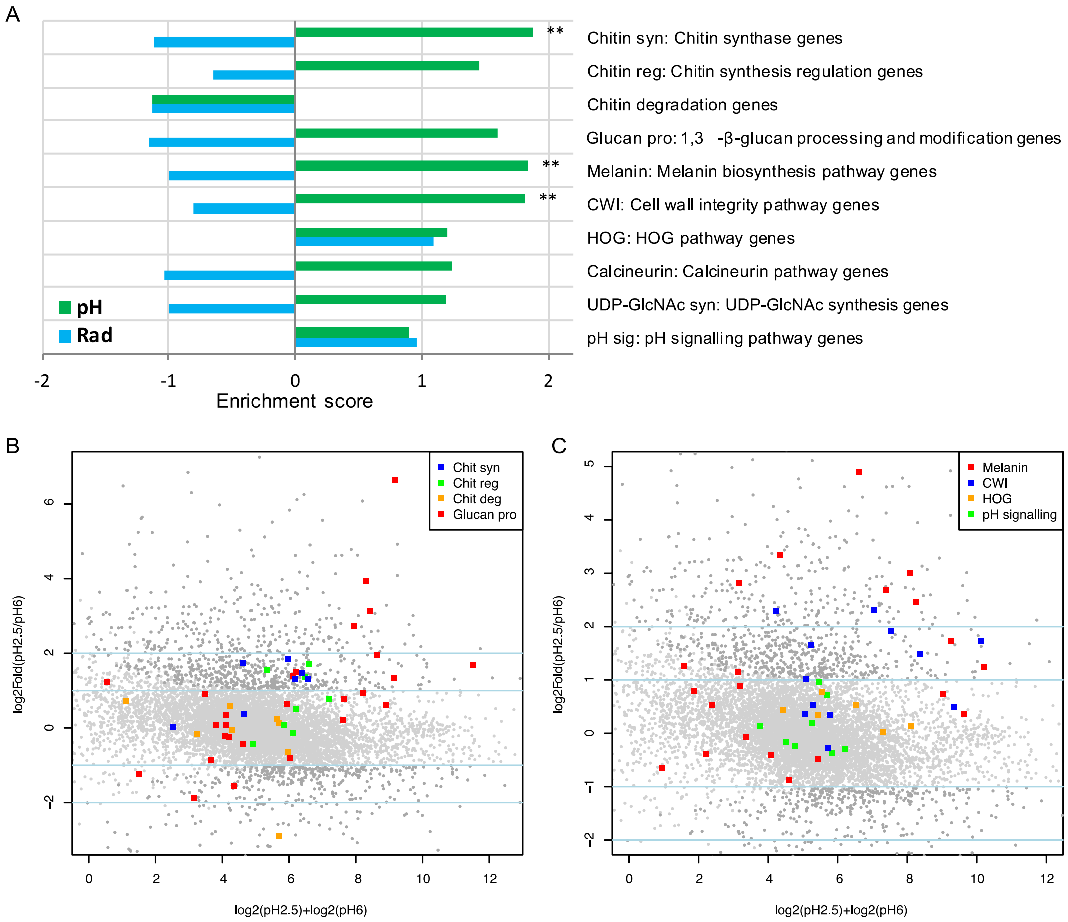

Figure S6 Enrichment of cell wall biosynthesis genes and stress response pathway genes under low pH and radiation stress. (**A**) GSEA enrichment score of different categories of genes or pathways. ** indicates significant enrichment with q-value < 0.05. (**B**) Distribution of different categories of cell wall biosynthesis genes on a MA plot, with light grey and dark grey dots representing the full gene set (dark grey indicates q-value < 1e-10). (**C**) Distribution of different stress response pathway genes on a MA plot.
