# Supplementary material for: Comparative Genomic and Transcriptomic Analysis of Wangiella dermatitidis, A Major Cause of Phaeohyphomycosis and a Model Black Yeast Human Pathogen
Source: G3 (Bethesda). 2014 Feb 4;4(4):561–78. doi: 10.1534/g3.113.009241 (PMC4059230; doi:10.1534/g3.113.009241)
Supplement: Supporting Information [file supp_g3.113.009241_TableS1.pdf]

Table S1 Protein domain enrichment and depletion in *W. dermatitidis* compared to other fungi.

Interpro Domain	Interpro Domain Counts per Genome											Enrichment and depletion in *W. dermatitidis* vs. all others				Enrichment and depletion in *W. dermatitidis* vs. *C. immitis*				Enrichment and depletion in *W. dermatitidis* and T. rubrum vs. all others			
	W. dermatitidis	*A. nidulans*	*A. niger*	*A. fumigatus*	*C. immitis*	*T. rubrum*	*M. thermophila*	*T. terrestris*	*N. crassa*	*S. cerevisiae*	*S. pombe*	Enrich pvalue	Enrich qvalue	Deplete pvalue	Deplete qvalue	Enrich pvalue	Enrich qvalue	Deplete pvalue	Deplete qvalue	Enrich pvalue	Enrich qvalue	Deplete pvalue	Deplete qvalue
**IPR domains enriched or depleted (pvalue < 0.05)**																							
IPR011701::Major facilitator superfamily MFS-1	230	261	318	211	109	128	120	166	114	43	51	2.1E-08	4.8E-05	1.0E+00	1.0E+00	6.5E-09	1.3E-05	1.0E+00	1.0E+00	2.1E-04	1.4E-01	1.0E+00	1.0E+00
IPR020635::Tyrosine-protein kinase, catalytic domain	99	71	17	57	87	92	96	98	34	41	3	6.1E-06	2.4E-03	1.0E+00	1.0E+00	4.6E-01	1.0E+00	6.6E-01	1.0E+00	5.5E-12	1.5E-08	1.0E+00	1.0E+00
IPR020843::Polyketide synthase, enoylreductase	46	49	32	25	25	24	29	36	6	6	1	5.2E-05	1.3E-02	1.0E+00	1.0E+00	2.5E-02	1.0E+00	9.9E-01	1.0E+00	3.8E-04	2.1E-01	1.0E+00	1.0E+00
IPR002290::Serine/threonine-protein kinase domain	117	95	70	87	144	136	124	138	76	87	73	1.6E-01	3.1E-01	8.8E-01	1.0E+00	9.9E-01	1.0E+00	1.0E-02	1.0E+00	5.0E-05	4.4E-02	1.0E+00	1.0E+00
IPR002575::Aminoglycoside phosphotransferase	22	7	29	15	62	51	26	42	3	1	0	6.2E-01	3.1E-01	5.6E-01	1.0E+00	1.0E+00	1.0E+00	2.4E-06	7.6E-03	2.5E-06	3.4E-03	1.0E+00	1.0E+00
IPR010730::Heterokaryon incompatibility	10	12	32	7	2	3	13	28	62	0	0	9.4E-01	3.6E-01	1.8E-01	1.0E+00	2.9E-02	1.0E+00	1.0E+00	1.0E+00	1.0E+00	4.3E-01	1.3E-03	5.0E-01
IPR017853::Glycoside hydrolase, catalytic core	65	136	121	126	37	43	84	93	82	26	28	9.4E-01	3.6E-01	1.0E-01	1.0E+00	1.5E-02	1.0E+00	1.0E+00	1.0E+00	1.0E+00	4.3E-01	4.4E-04	2.1E-01
IPR000992::Stress-induced protein SRP1/TIP1	0	0	0	0	0	0	0	0	0	32	0	9.5E-01	3.6E-01	4.7E-02	1.0E+00	1.0E+00	1.0E+00	1.0E+00	1.0E+00	1.0E+00	4.3E-01	2.5E-03	8.0E-01
IPR005197::Glycoside hydrolase, family 71	0	5	7	8	1	0	3	1	6	0	2	9.5E-01	3.6E-01	4.7E-02	1.0E+00	5.3E-01	1.0E+00	4.7E-01	1.0E+00	1.0E+00	4.3E-01	2.5E-03	8.0E-01
IPR013781::Glycoside hydrolase, subgroup, catalytic core	45	93	82	92	29	34	61	68	62	25	22	9.5E-01	3.6E-01	8.1E-02	1.0E+00	9.5E-02	1.0E+00	9.6E-01	1.0E+00	1.0E+00	4.3E-01	2.6E-03	8.0E-01
IPR006626::Parallel beta-helix repeat	0	9	12	11	0	0	1	2	3	1	0	9.7E-01	3.7E-01	2.7E-02	1.0E+00	1.0E+00	1.0E+00	1.0E+00	1.0E+00	1.0E+00	4.3E-01	8.4E-04	3.5E-01
IPR004906::Pogo transposase / Cenp-B / PDC2, subgroup, DNA-binding HTH domain	0	4	1	19	2	0	2	2	3	1	3	9.8E-01	3.7E-01	2.3E-02	1.0E+00	7.7E-01	1.0E+00	2.3E-01	1.0E+00	1.0E+00	4.3E-01	5.8E-04	2.6E-01
IPR007889::Helix-turn-helix, Psq	0	0	6	30	0	0	2	1	0	0	0	9.8E-01	3.7E-01	1.9E-02	1.0E+00	1.0E+00	1.0E+00	1.0E+00	1.0E+00	1.0E+00	4.3E-01	4.1E-04	2.1E-01
IPR000743::Glycoside hydrolase, family 28	0	11	20	12	0	0	2	7	2	1	0	9.9E-01	3.7E-01	6.2E-03	1.0E+00	1.0E+00	1.0E+00	1.0E+00	1.0E+00	1.0E+00	4.3E-01	4.6E-05	3.2E-02
IPR000254::Cellulose-binding domain, fungal	1	6	8	17	0	0	16	25	21	0	1	1.0E+00	3.7E-01	8.0E-03	1.0E+00	5.3E-01	1.0E+00	1.0E+00	1.0E+00	1.0E+00	4.3E-01	6.3E-06	5.6E-03
IPR000477::Reverse transcriptase	0	4	11	24	1	1	21	41	2	5	14	1.0E+00	3.7E-01	6.6E-06	4.1E-02	5.3E-01	1.0E+00	4.7E-01	1.0E+00	1.0E+00	4.3E-01	2.3E-08	1.5E-04
IPR000845::Nucleoside phosphorylase domain	2	23	34	10	3	2	5	1	4	2	2	1.0E+00	3.7E-01	3.7E-02	1.0E+00	8.4E-01	1.0E+00	7.8E-01	1.0E+00	1.0E+00	4.3E-01	1.5E-03	5.5E-01
IPR001584::Integrase, catalytic core	0	0	16	4	0	0	3	4	0	44	11	1.0E+00	3.7E-01	3.5E-04	7.6E-01	1.0E+00	1.0E+00	1.0E+00	1.0E+00	1.0E+00	4.3E-01	1.6E-07	4.3E-04
IPR004875::DDE superfamily endonuclease, CENP-B-like	0	3	14	49	2	1	1	0	0	1	3	1.0E+00	3.7E-01	5.1E-04	7.6E-01	7.7E-01	1.0E+00	2.3E-01	1.0E+00	1.0E+00	4.3E-01	5.1E-05	3.2E-02
IPR005103::Glycoside hydrolase, family 61	1	10	7	7	0	0	22	18	14	0	0	1.0E+00	3.7E-01	2.5E-02	1.0E+00	5.3E-01	1.0E+00	1.0E+00	1.0E+00	1.0E+00	4.3E-01	8.1E-05	4.6E-02
IPR006600::Pogo transposase / Cenp-B / PDC2, DNA-binding HTH domain	0	4	6	42	2	0	2	2	3	1	3	1.0E+00	3.7E-01	1.2E-03	1.0E+00	7.7E-01	1.0E+00	2.3E-01	1.0E+00	1.0E+00	4.3E-01	1.7E-06	1.8E-03
IPR011050::Pectin lyase fold/virulence factor	3	36	32	34	1	1	16	16	12	2	0	1.0E+00	3.7E-01	1.9E-03	1.0E+00	3.5E-01	1.0E+00	1.0E+00	1.0E+00	1.0E+00	4.3E-01	2.1E-07	4.3E-04
IPR012334::Pectin lyase fold	3	35	32	34	1	1	15	17	12	1	0	1.0E+00	3.7E-01	2.1E-03	1.0E+00	3.5E-01	1.0E+00	1.0E+00	1.0E+00	1.0E+00	4.3E-01	2.8E-07	4.4E-04
IPR013103::Reverse transcriptase, RNA-dependent DNA polymerase	0	1	16	0	0	0	1	2	0	42	0	1.0E+00	3.7E-01	2.9E-03	1.0E+00	1.0E+00	1.0E+00	1.0E+00	1.0E+00	1.0E+00	4.3E-01	1.1E-05	8.3E-03
IPR015820::Retrotransposon Ty1 A, N-terminal	0	0	0	0	0	0	0	0	0	80	0	1.0E+00	3.7E-01	6.1E-04	7.6E-01	1.0E+00	1.0E+00	1.0E+00	1.0E+00	1.0E+00	4.3E-01	4.9E-07	6.1E-04
**Glycoside hydrolase family**																							
IPR000933::Glycoside hydrolase, family 29	0	0	1	0	0	0	0	0	0	0	0	8.8E-02	3.1E-01	9.1E-01	1.0E+00	1.0E+00	1.0E+00	1.0E+00	1.0E+00	1.7E-01	3.6E-01	8.3E-01	1.0E+00
IPR016286::Glycoside hydrolase, family 29, bacteria/metazoa/fungi	0	0	1	0	0	0	0	0	0	0	0	8.8E-02	3.1E-01	9.1E-01	1.0E+00	1.0E+00	1.0E+00	1.0E+00	1.0E+00	1.7E-01	3.6E-01	8.3E-01	1.0E+00
IPR013529::Glycoside hydrolase, family 42, N-terminal	1	0	0	1	0	0	0	0	0	0	0	1.7E-01	3.1E-01	1.0E+00	1.0E+00	5.3E-01	1.0E+00	1.0E+00	1.0E+00	3.0E-01	3.6E-01	1.0E+00	1.0E+00
IPR016282::Glycoside hydrolase, family 5, endoglucanase B	0	1	0	1	0	0	0	0	0	0	0	1.7E-01	3.1E-01	8.3E-01	1.0E+00	1.0E+00	1.0E+00	1.0E+00	1.0E+00	3.0E-01	3.6E-01	7.0E-01	1.0E+00
IPR018087::Glycoside hydrolase, family 5, conserved site	3	0	1	2	1	0	0	3	0	4	1	2.3E-01	3.1E-01	9.8E-01	1.0E+00	3.5E-01	1.0E+00	1.0E+00	1.0E+00	6.3E-01	3.6E-01	8.0E-01	1.0E+00
IPR000757::Glycoside hydrolase, family 16	12	12	10	11	6	7	14	13	15	3	1	2.4E-01	3.1E-01	9.1E-01	1.0E+00	1.7E-01	1.0E+00	9.7E-01	1.0E+00	4.1E-01	3.6E-01	7.7E-01	1.0E+00
IPR005089::Glycoside hydrolase, family 18, carbohydrate-binding	0	0	0	1	0	0	1	0	0	1	0	2.4E-01	3.1E-01	7.6E-01	1.0E+00	1.0E+00	1.0E+00	1.0E+00	1.0E+00	4.2E-01	3.6E-01	5.8E-01	1.0E+00
IPR006215::Glycoside hydrolase, melibiase	1	0	0	1	0	0	0	0	0	0	1	2.4E-01	3.1E-01	1.0E+00	1.0E+00	5.3E-01	1.0E+00	1.0E+00	1.0E+00	4.2E-01	3.6E-01	1.0E+00	1.0E+00
IPR011330::Glycoside hydrolase/deacetylase, beta/alpha-barrel	9	11	10	9	5	6	7	7	7	3	2	2.5E-01	3.1E-01	9.2E-01	1.0E+00	2.7E-01	1.0E+00	9.6E-01	1.0E+00	3.1E-01	3.6E-01	8.6E-01	1.0E+00
IPR000490::Glycoside hydrolase, family 17	4	1	3	2	3	2	3	2	3	4	1	3.1E-01	3.1E-01	9.4E-01	1.0E+00	5.6E-01	1.0E+00	9.2E-01	1.0E+00	4.4E-01	3.6E-01	8.5E-01	1.0E+00
IPR005194::Glycoside hydrolase, family 65, C-terminal	0	1	0	1	1	0	0	0	0	1	0	3.1E-01	3.1E-01	6.9E-01	1.0E+00	5.3E-01	1.0E+00	4.7E-01	1.0E+00	5.2E-01	3.6E-01	4.8E-01	1.0E+00
IPR000726::Glycoside hydrolase, family 19, catalytic	0	0	0	0	2	1	1	1	0	0	0	3.7E-01	3.1E-01	6.3E-01	1.0E+00	7.7E-01	1.0E+00	2.3E-01	1.0E+00	6.0E-01	3.6E-01	9.6E-01	1.0E+00
IPR000805::Glycoside hydrolase, family 26	0	3	1	0	0	0	1	0	0	0	0	3.7E-01	3.1E-01	6.3E-01	1.0E+00	1.0E+00	1.0E+00	1.0E+00	1.0E+00	6.0E-01	3.6E-01	4.0E-01	1.0E+00
IPR002196::Glycoside hydrolase, family 24	0	1	0	0	0	2	1	1	0	0	0	3.7E-01	3.1E-01	6.3E-01	1.0E+00	1.0E+00	1.0E+00	1.0E+00	1.0E+00	2.0E-01	3.6E-01	1.0E+00	1.0E+00
IPR008270::Glycoside hydrolase, family 25, active site	0	2	0	0	0	0	1	1	1	0	0	3.7E-01	3.1E-01	6.3E-01	1.0E+00	1.0E+00	1.0E+00	1.0E+00	1.0E+00	6.0E-01	3.6E-01	4.0E-01	1.0E+00
IPR001360::Glycoside hydrolase, family 1	2	3	3	5	0	0	1	1	1	0	0	4.2E-01	3.1E-01	9.5E-01	1.0E+00	2.8E-01	1.0E+00	1.0E+00	1.0E+00	7.7E-01	3.7E-01	7.3E-01	1.0E+00
IPR000334::Glycoside hydrolase, family 45	0	1	0	1	0	0	1	2	1	0	0	4.3E-01	3.1E-01	5.7E-01	1.0E+00	1.0E+00	1.0E+00	1.0E+00	1.0E+00	6.6E-01	3.6E-01	3.4E-01	1.0E+00
IPR005154::Glycoside hydrolase, family 67, alpha glucuronidase, N-terminal	1	1	1	1	0	0	1	0	1	0	0	4.3E-01	3.1E-01	9.9E-01	1.0E+00	5.3E-01	1.0E+00	1.0E+00	1.0E+00	6.6E-01	3.6E-01	9.4E-01	1.0E+00
IPR011099::Glycosyl hydrolase 67, C-terminal	1	1	1	1	0	0	1	0	1	0	0	4.3E-01	3.1E-01	9.9E-01	1.0E+00	5.3E-01	1.0E+00	1.0E+00	1.0E+00	6.6E-01	3.6E-01	9.4E-01	1.0E+00
IPR011100::Glycosyl hydrolase 67 middle	1	1	1	1	0	0	1	0	1	0	0	4.3E-01	3.1E-01	9.9E-01	1.0E+00	5.3E-01	1.0E+00	1.0E+00	1.0E+00	6.6E-01	3.6E-01	9.4E-01	1.0E+00
IPR011395::Glycoside hydrolase, family 67, alpha glucuronidase	1	1	1	1	0	0	1	0	1	0	0	4.3E-01	3.1E-01	9.9E-01	1.0E+00	5.3E-01	1.0E+00	1.0E+00	1.0E+00	6.6E-01	3.6E-01	9.4E-01	1.0E+00
IPR022790::Endoglucanase H/Glycosyl hydrolase family 26	0	3	1	0	0	0	1	0	1	0	0	4.3E-01	3.1E-01	5.7E-01	1.0E+00	1.0E+00	1.0E+00	1.0E+00	1.0E+00	6.6E-01	3.6E-01	3.4E-01	1.0E+00
IPR000322::Glycoside hydrolase, family 31	6	10	7	7	2	2	6	8	5	1	4	4.5E-01	3.1E-01	8.3E-01	1.0E+00	1.8E-01	1.0E+00	9.9E-01	1.0E+00	8.2E-01	3.8E-01	4.3E-01	1.0E+00
IPR002594::Glycoside hydrolase, family 12	2	1	4	4	0	0	1	3	2	0	0	4.5E-01	3.1E-01	9.4E-01	1.0E+00	2.8E-01	1.0E+00	1.0E+00	1.0E+00	8.0E-01	3.8E-01	6.9E-01	1.0E+00
IPR006589::Glycosyl hydrolase, family 13, subfamily, catalytic domain	8	11	10	10	4	2	7	4	9	7	8	4.5E-01	3.1E-01	8.0E-01	1.0E+00	2.5E-01	1.0E+00	9.7E-01	1.0E+00	9.0E-01	4.1E-01	2.6E-01	1.0E+00
IPR001540::Glycoside hydrolase, family 20	2	2	3	2	3	2	1	1	1	0	0	4.8E-01	3.1E-01	9.3E-01	1.0E+00	8.4E-01	1.0E+00	7.8E-01	1.0E+00	3.5E-01	3.6E-01	9.4E-01	1.0E+00
IPR002252::Glycoside hydrolase, family 36	1	3	1	2	0	0	0	0	0	0	0	4.8E-01	3.1E-01	9.8E-01	1.0E+00	5.3E-01	1.0E+00	1.0E+00	1.0E+00	7.2E-01	3.6E-01	9.1E-01	1.0E+00
IPR006046::Glycoside hydrolase, family 13	0	3	2	1	0	0	0	1	0	0	0	4.8E-01	3.1E-01	5.2E-01	1.0E+00	1.0E+00	1.0E+00	1.0E+00	1.0E+00	7.2E-01	3.6E-01	2.8E-01	1.0E+00
IPR011683::Glycosyl hydrolase 53	0	1	2	1	0	0	1	1	1	0	0	4.8E-01	3.1E-01	5.2E-01	1.0E+00	1.0E+00	1.0E+00	1.0E+00	1.0E+00	7.2E-01	3.6E-01	2.8E-01	1.0E+00
IPR015883::Glycoside hydrolase, family 20, catalytic core	2	2	3	2	3	2	1	1	1	0	0	4.8E-01	3.1E-01	9.3E-01	1.0E+00	8.4E-01	1.0E+00	7.8E-01	1.0E+00	3.5E-01	3.6E-01	9.4E-01	1.0E+00
IPR018053::Glycoside hydrolase, family 32, active site	0	1	1	2	0	0	0	0	0	1	2	4.8E-01	3.1E-01	5.2E-01	1.0E+00	1.0E+00	1.0E+00	1.0E+00	1.0E+00	7.2E-01	3.6E-01	2.8E-01	1.0E+00
IPR019801::Glycoside hydrolase, family 35, conserved site	1	0	2	2	0	0	0	1	1	0	0	4.8E-01	3.1E-01	9.8E-01	1.0E+00	5.3E-01	1.0E+00	1.0E+00	1.0E+00	7.2E-01	3.6E-01	9.1E-01	1.0E+00
IPR004199::Glycoside hydrolase, family 42, domain 5	1	2	0	1	0	0	1	1	2	0	0	5.2E-01	3.1E-01	9.7E-01	1.0E+00	5.3E-01	1.0E+00	1.0E+00	1.0E+00	7.7E-01	3.7E-01	8.7E-01	1.0E+00
IPR005195::Glycoside hydrolase, family 65, central catalytic	1	1	1	1	1	1	0	0	0	1	0	5.2E-01	3.1E-01	9.7E-01	1.0E+00	7.7E-01	1.0E+00	1.0E+00	1.0E+00	3.9E-01	3.6E-01	9.7E-01	1.0E+00
IPR005196::Glycoside hydrolase, family 65, N-terminal	1	1	1	1	1	1	0	0	0	1	0	5.2E-01	3.1E-01	9.7E-01	1.0E+00	7.7E-01	1.0E+00	1.0E+00	1.0E+00	3.9E-01	3.6E-01	9.7E-01	1.0E+00
IPR014718::Glycoside hydrolase-type carbohydrate-binding, subgroup	4	5	3	4	3	3	5	4	6	4	1	5.5E-01	3.1E-01	8.1E-01	1.0E+00	5.6E-01	1.0E+00	9.2E-01	1.0E+00	6.1E-01	3.6E-01	7.0E-01	1.0E+00
IPR001139::Glycoside hydrolase, family 30	1	0	1	1	0	0	2	3	1	0	0	5.6E-01	3.1E-01	9.6E-01	1.0E+00	5.3E-01	1.0E+00	1.0E+00	1.0E+00	8.0E-01	3.8E-01	8.2E-01	1.0E+00
IPR004888::Glycoside hydrolase, family 63	2	2	2	1	1	1	2	3	3	1	1	5.6E-01	3.1E-01	8.9E-01	1.0E+00	5.4E-01	1.0E+00	1.0E+00	1.0E+00	7.0E-01	3.6E-01	7.4E-01	1.0E+00
IPR005193::Glycoside hydrolase, family 62, arabinosidase	0	2	1	2	0	0	2	2	0	0	0	5.6E-01	3.1E-01	4.4E-01	1.0E+00	1.0E+00	1.0E+00	1.0E+00	1.0E+00	8.0E-01	3.8E-01	2.0E-01	1.0E+00
IPR002053::Glycoside hydrolase, family 25	0	3	0	3	0	1	1	1	1	0	0	6.0E-01	3.1E-01	4.0E-01	1.0E+00	1.0E+00	1.0E+00	1.0E+00	1.0E+00	8.4E-01	3.9E-01	7.8E-01	1.0E+00
IPR018077::Glycoside hydrolase, family 25 subgroup	0	3	0	3	0	1	1	1	1	0	0	6.0E-01	3.1E-01	4.0E-01	1.0E+00	1.0E+00	1.0E+00	1.0E+00	1.0E+00	8.4E-01	3.9E-01	7.8E-01	1.0E+00
IPR018120::Glycoside hydrolase, family 1, active site	1	1	2	3	0	0	1	1	1	0	0	6.0E-01	3.1E-01	9.5E-01	1.0E+00	5.3E-01	1.0E+00	1.0E+00	1.0E+00	8.4E-01	3.9E-01	7.8E-01	1.0E+00
IPR011013::Glycoside hydrolase-type carbohydrate-binding	11	20	14	15	6	7	13	14	13	6	6	6.1E-01	3.1E-01	6.3E-01	1.0E+00	2.2E-01	1.0E+00	9.6E-01	1.0E+00	8.4E-01	3.9E-01	3.0E-01	1.0E+00
IPR000602::Glycoside hydrolase, family 38, core	1	1	1	1	1	1	1	1	1	1	1	6.7E-01	3.1E-01	9.2E-01	1.0E+00	7.7E-01	1.0E+00	1.0E+00	1.0E+00	6.2E-01	3.6E-01	8.8E-01	1.0E+00
IPR004193::Glycoside hydrolase, family 13, N-terminal	1	1	1	1	1	1	1	1	2	1	0	6.7E-01	3.1E-01	9.2E-01	1.0E+00	7.7E-01	1.0E+00	1.0E+00	1.0E+00	6.2E-01	3.6E-01	8.8E-01	1.0E+00
IPR011682::Glycosyl hydrolases 38, C-terminal	1	1	1	1	1	1	1	1	1	1	1	6.7E-01	3.1E-01	9.2E-01	1.0E+00	7.7E-01	1.0E+00	1.0E+00	1.0E+00	6.2E-01	3.6E-01	8.8E-01	1.0E+00
IPR015341::Glycoside hydrolase, family 38, central domain	1	1	1	1	1	1	1	1	1	1	1	6.7E-01	3.1E-01	9.2E-01	1.0E+00	7.7E-01	1.0E+00	1.0E+00	1.0E+00	6.2E-01	3.6E-01	8.8E-01	1.0E+00
IPR016840::Glycoside hydrolase, family 43, endo-1, 5-alpha-L-arabinosidase	0	4	3	2	1	0	1	1	0	0	0	6.7E-01	3.1E-01	3.3E-01	1.0E+00	5.3E-01	1.0E+00	4.7E-01	1.0E+00	8.9E-01	4.0E-01	1.1E-01	1.0E+00
IPR000111::Glycoside hydrolase, clan GH-D	2	6	7	7	0	0	2	1	0	0	1	6.8E-01	3.1E-01	8.1E-01	1.0E+00	2.8E-01	1.0E+00	1.0E+00	1.0E+00	9.5E-01	4.2E-01	3.5E-01	1.0E+00
IPR001524::Glycoside hydrolase, family 6, conserved site	0	2	2	1	0	0	3	2	3	0	0	7.0E-01	3.1E-01	3.0E-01	1.0E+00	1.0E+00	1.0E+00	1.0E+00	1.0E+00	9.1E-01	4.1E-01	9.4E-02	1.0E+00
IPR006047::Glycosyl hydrolase, family 13, catalytic domain	8	12	17	15	4	2	7	4	10	9	12	7.0E-01	3.1E-01	5.7E-01	1.0E+00	2.5E-01	1.0E+00	9.7E-01	1.0E+00	9.8E-01	4.3E-01	6.3E-02	1.0E+00
IPR017168::Glycoside hydrolase, family 16, CRH1, predicted	2	3	3	3	2	0	3	3	3	2	0	7.0E-01	3.1E-01	7.9E-01	1.0E+00	7.2E-01	1.0E+00	9.2E-01	1.0E+00	9.5E-01	4.3E-01	3.2E-01	1.0E+00
IPR005198::Glycoside hydrolase, family 76	6	7	11	8	3	3	9	9	10	2	4	7.1E-01	3.1E-01	6.0E-01	1.0E+00	3.1E-01	1.0E+00	9.7E-01	1.0E+00	9.2E-01	4.2E-01	2.2E-01	1.0E+00
IPR013189::Glycosyl hydrolase family 32, C-terminal	1	2	5	2	0	0	0	1	1	1	1	7.3E-01	3.1E-01	8.8E-01	1.0E+00	5.3E-01	1.0E+00	1.0E+00	1.0E+00	9.2E-01	4.1E-01	5.8E-01	1.0E+00
IPR000165::Glycoside hydrolase, family 15	0	2	1	3	1	1	1	2	1	1	1	7.5E-01	3.1E-01	2.5E-01	1.0E+00	5.3E-01	1.0E+00	4.7E-01	1.0E+00	9.3E-01	4.2E-01	5.3E-01	1.0E+00
IPR005200::Glycoside hydrolase, family 81	1	1	1	1	1	1	1	1	1	2	2	7.5E-01	3.1E-01	8.6E-01	1.0E+00	7.7E-01	1.0E+00	1.0E+00	1.0E+00	7.4E-01	3.6E-01	7.7E-01	1.0E+00
IPR006101::Glycoside hydrolase, family 2	1	4	1	1	0	0	3	3	2	0	0	7.5E-01	3.1E-01	8.6E-01	1.0E+00	5.3E-01	1.0E+00	1.0E+00	1.0E+00	9.3E-01	4.2E-01	5.3E-01	1.0E+00
IPR013780::Glycosyl hydrolase, family 13, all-beta	8	11	18	18	3	2	10	8	10	8	9	7.5E-01	3.1E-01	5.1E-01	1.0E+00	1.5E-01	1.0E+00	9.9E-01	1.0E+00	9.9E-01	4.3E-01	4.2E-02	1.0E+00
IPR001661::Glycoside hydrolase, family 37	1	1	1	1	1	1	2	2	2	2	1	7.7E-01	3.1E-01	8.4E-01	1.0E+00	7.7E-01	1.0E+00	1.0E+00	1.0E+00	7.7E-01	3.7E-01	7.3E-01	1.0E+00
IPR018232::Glycoside hydrolase, family 37, conserved site	1	1	1	1	1	1	2	2	2	2	1	7.7E-01	3.1E-01	8.4E-01	1.0E+00	7.7E-01	1.0E+00	1.0E+00	1.0E+00	7.7E-01	3.7E-01	7.3E-01	1.0E+00
IPR001382::Glycoside hydrolase, family 47	5	8	5	6	7	6	9	10	9	3	2	7.8E-01	3.1E-01	5.3E-01	1.0E+00	8.5E-01	1.0E+00	5.4E-01	1.0E+00	6.9E-01	3.6E-01	5.6E-01	1.0E+00
IPR001362::Glycoside hydrolase, family 32	1	2	5	4	0	0	0	1	1	1	2	7.9E-01	3.2E-01	8.1E-01	1.0E+00	5.3E-01	1.0E+00	1.0E+00	1.0E+00	9.5E-01	4.3E-01	4.5E-01	1.0E+00
IPR002241::Glycoside hydrolase, family 27	1	3	5	5	0	0	2	1	0	0	1	8.1E-01	3.2E-01	7.9E-01	1.0E+00	5.3E-01	1.0E+00	1.0E+00	1.0E+00	9.6E-01	4.3E-01	4.1E-01	1.0E+00
IPR018208::Glycoside hydrolase, family 11, active site	0	2	3	3	0	0	6	2	2	0	0	8.1E-01	3.2E-01	1.9E-01	1.0E+00	1.0E+00	1.0E+00	1.0E+00	1.0E+00	9.6E-01	4.3E-01	3.8E-02	1.0E+00
IPR001944::Glycoside hydrolase, family 35	1	4	5	4	0	0	1	2	2	0	0	8.3E-01	3.2E-01	7.7E-01	1.0E+00	5.3E-01	1.0E+00	1.0E+00	1.0E+00	9.7E-01	4.3E-01	3.7E-01	1.0E+00
IPR002044::Glycoside hydrolase, carbohydrate-binding	0	4	1	4	1	1	2	3	3	0	0	8.3E-01	3.2E-01	1.7E-01	1.0E+00	5.3E-01	1.0E+00	4.7E-01	1.0E+00	9.7E-01	4.3E-01	3.7E-01	1.0E+00
IPR010905::Glycosyl hydrolase, family 88	1	6	3	5	0	0	1	1	2	0	0	8.3E-01	3.2E-01	7.7E-01	1.0E+00	5.3E-01	1.0E+00	1.0E+00	1.0E+00	9.7E-01	4.3E-01	3.7E-01	1.0E+00
IPR013148::Glycosyl hydrolases family 32, N-terminal	1	2	5	4	0	0	0	1	3	1	2	8.3E-01	3.2E-01	7.7E-01	1.0E+00	5.3E-01	1.0E+00	1.0E+00	1.0E+00	9.7E-01	4.3E-01	3.7E-01	1.0E+00
IPR019800::Glycoside hydrolase, family 3, active site	2	7	3	5	2	2	3	4	4	0	1	8.3E-01	3.2E-01	6.3E-01	1.0E+00	7.2E-01	1.0E+00	9.2E-01	1.0E+00	8.6E-01	3.9E-01	4.6E-01	1.0E+00
IPR002772::Glycoside hydrolase, family 3, C-terminal	6	19	15	16	3	5	10	11	8	0	1	8.6E-01	3.3E-01	3.8E-01	1.0E+00	3.1E-01	1.0E+00	9.7E-01	1.0E+00	9.4E-01	4.2E-01	1.7E-01	1.0E+00
IPR001000::Glycoside hydrolase, family 10	1	3	1	4	0	0	4	6	4	0	0	8.8E-01	3.4E-01	6.7E-01	1.0E+00	5.3E-01	1.0E+00	1.0E+00	1.0E+00	9.8E-01	4.3E-01	2.4E-01	1.0E+00
IPR011613::Glycoside hydrolase 15-related	1	2	2	5	1	1	2	3	2	1	2	8.8E-01	3.4E-01	6.7E-01	1.0E+00	7.7E-01	1.0E+00	1.0E+00	1.0E+00	9.1E-01	4.1E-01	4.5E-01	1.0E+00
IPR001137::Glycoside hydrolase, family 11	0	2	4	3	0	0	8	5	2	0	0	8.9E-01	3.4E-01	1.1E-01	1.0E+00	1.0E+00	1.0E+00	1.0E+00	1.0E+00	9.9E-01	4.3E-01	1.3E-02	1.0E+00
IPR001547::Glycoside hydrolase, family 5	5	15	10	13	2	3	9	13	6	4	3	8.9E-01	3.4E-01	3.6E-01	1.0E+00	2.7E-01	1.0E+00	9.9E-01	1.0E+00	9.8E-01	4.3E-01	7.7E-02	1.0E+00
IPR001764::Glycoside hydrolase, family 3, N-terminal	7	21	17	18	5	6	12	14	10	0	1	8.9E-01	3.4E-01	3.1E-01	1.0E+00	4.6E-01	1.0E+00	9.0E-01	1.0E+00	9.5E-01	4.3E-01	1.3E-01	1.0E+00
IPR013319::Glycoside hydrolase, family 11/12, catalytic domain	2	3	8	7	0	0	9	8	4	0	0	8.9E-01	3.4E-01	5.0E-01	1.0E+00	2.8E-01	1.0E+00	1.0E+00	1.0E+00	9.9E-01	4.3E-01	7.3E-02	1.0E+00
IPR001722::Glycoside hydrolase, family 7	0	3	2	4	0	0	6	5	5	0	0	9.0E-01	3.5E-01	9.9E-02	1.0E+00	1.0E+00	1.0E+00	1.0E+00	1.0E+00	9.9E-01	4.3E-01	1.1E-02	1.0E+00
IPR006104::Glycoside hydrolase, family 2, carbohydrate-binding	2	9	5	5	1	2	7	7	5	0	0	9.0E-01	3.5E-01	4.7E-01	1.0E+00	5.4E-01	1.0E+00	1.0E+00	1.0E+00	9.4E-01	4.2E-01	2.6E-01	1.0E+00
IPR006710::Glycoside hydrolase, family 43	4	17	10	17	1	1	11	8	5	0	0	9.0E-01	3.5E-01	3.5E-01	1.0E+00	2.2E-01	1.0E+00	1.0E+00	1.0E+00	1.0E+00	4.3E-01	2.8E-02	1.0E+00
IPR012939::Glycosyl hydrolase 92	0	5	5	5	1	2	2	2	2	0	0	9.0E-01	3.5E-01	9.9E-02	1.0E+00	5.3E-01	1.0E+00	4.7E-01	1.0E+00	9.4E-01	4.2E-01	3.8E-01	1.0E+00
IPR001579::Glycoside hydrolase, chitinase active site	5	16	11	15	7	11	5	12	7	2	0	9.3E-01	3.6E-01	2.5E-01	1.0E+00	8.5E-01	1.0E+00	5.4E-01	1.0E+00	5.4E-01	3.6E-01	6.7E-01	1.0E+00
IPR006102::Glycoside hydrolase, family 2, immunoglobulin-like beta-sandwich	2	10	6	6	1	2	7	8	5	0	0	9.3E-01	3.6E-01	3.8E-01	1.0E+00	5.4E-01	1.0E+00	1.0E+00	1.0E+00	9.7E-01	4.3E-01	1.7E-01	1.0E+00
IPR006103::Glycoside hydrolase, family 2, TIM barrel	1	7	3	2	1	1	5	6	3	0	0	9.3E-01	3.6E-01	5.2E-01	1.0E+00	7.7E-01	1.0E+00	1.0E+00	1.0E+00	9.7E-01	4.3E-01	2.7E-01	1.0E+00
IPR013812::Glycoside hydrolase, family 2/20, immunoglobulin-like beta-sandwich domain	2	10	6	6	1	2	7	8	5	0	0	9.3E-01	3.6E-01	3.8E-01	1.0E+00	5.4E-01	1.0E+00	1.0E+00	1.0E+00	9.7E-01	4.3E-01	1.7E-01	1.0E+00
IPR017853::Glycoside hydrolase, catalytic core	65	136	121	126	37	43	84	93	82	26	28	9.4E-01	3.6E-01	1.0E-01	1.0E+00	1.5E-02	1.0E+00	1.0E+00	1.0E+00	1.0E+00	4.3E-01	4.4E-04	2.1E-01
IPR005197::Glycoside hydrolase, family 71	0	5	7	8	1	0	3	1	6	0	2	9.5E-01	3.6E-01	4.7E-02	1.0E+00	5.3E-01	1.0E+00	4.7E-01	1.0E+00	1.0E+00	4.3E-01	2.5E-03	8.0E-01
IPR013781::Glycoside hydrolase, subgroup, catalytic core	45	93	82	92	29	34	61	68	62	25	22	9.5E-01	3.6E-01	8.1E-02	1.0E+00	9.5E-02	1.0E+00	9.6E-01	1.0E+00	1.0E+00	4.3E-01	2.6E-03	8.0E-01
IPR001223::Glycoside hydrolase, family 18, catalytic domain	6	20	14	18	9	14	10	15	12	1	0	9.6E-01	3.7E-01	1.5E-01	1.0E+00	8.9E-01	1.0E+00	4.2E-01	1.0E+00	5.6E-01	3.6E-01	6.3E-01	1.0E+00
IPR000743::Glycoside hydrolase, family 28	0	11	20	12	0	0	2	7	2	1	0	9.9E-01	3.7E-01	6.2E-03	1.0E+00	1.0E+00	1.0E+00	1.0E+00	1.0E+00	1.0E+00	4.3E-01	4.6E-05	3.2E-02
IPR002037::Glycoside hydrolase, family 8	0	0	0	0	0	0	0	0	0	0	0	1.0E+00	3.7E-01	1.0E+00	1.0E+00	1.0E+00	1.0E+00	1.0E+00	1.0E+00	1.0E+00	4.3E-01	1.0E+00	1.0E+00
IPR005103::Glycoside hydrolase, family 61	1	10	7	7	0	0	22	18	14	0	0	1.0E+00	3.7E-01	2.5E-02	1.0E+00	5.3E-01	1.0E+00	1.0E+00	1.0E+00	1.0E+00	4.3E-01	8.1E-05	4.6E-02
IPR005201::Glycoside hydrolase, family 85	0	0	0	0	0	0	0	0	0	0	0	1.0E+00	3.7E-01	1.0E+00	1.0E+00	1.0E+00	1.0E+00	1.0E+00	1.0E+00	1.0E+00	4.3E-01	1.0E+00	1.0E+00

**Peptidase family**																							
IPR008257::Peptidase M19, renal dipeptidase	4	1	1	2	2	3	1	1	1	0	2	6.8E-02	3.1E-01	1.0E+00	1.0E+00	3.9E-01	1.0E+00	9.8E-01	1.0E+00	2.0E-02	3.6E-01	1.0E+00	1.0E+00
IPR005079::Peptidase C45, acyl-coenzyme A:6-aminopenicillanic acid acyl-transferase	3	2	1	1	1	2	1	1	0	0	0	8.3E-02	3.1E-01	1.0E+00	1.0E+00	3.5E-01	1.0E+00	1.0E+00	1.0E+00	3.6E-02	3.6E-01	1.0E+00	1.0E+00
IPR000819::Peptidase M17, leucyl aminopeptidase, C-terminal	0	0	0	0	0	0	0	0	0	0	1	8.8E-02	3.1E-01	9.1E-01	1.0E+00	1.0E+00	1.0E+00	1.0E+00	1.0E+00	1.7E-01	3.6E-01	8.3E-01	1.0E+00
IPR001314::Peptidase S1A, chymotrypsin	0	1	0	0	0	0	0	0	0	0	0	8.8E-02	3.1E-01	9.1E-01	1.0E+00	1.0E+00	1.0E+00	1.0E+00	1.0E+00	1.7E-01	3.6E-01	8.3E-01	1.0E+00
IPR001818::Peptidase M10, metallopeptidase	0	1	0	0	0	0	0	0	0	0	0	8.8E-02	3.1E-01	9.1E-01	1.0E+00	1.0E+00	1.0E+00	1.0E+00	1.0E+00	1.7E-01	3.6E-01	8.3E-01	1.0E+00
IPR002138::Peptidase C14, caspase non-catalytic subunit p10	0	0	0	0	0	1	0	0	0	0	0	8.8E-02	3.1E-01	9.1E-01	1.0E+00	1.0E+00	1.0E+00	1.0E+00	1.0E+00	1.7E-01	3.6E-01	1.0E+00	1.0E+00
IPR005944::Peptidase S33, proline iminopeptidase 1	0	0	0	0	0	1	0	0	0	0	0	8.8E-02	3.1E-01	9.1E-01	1.0E+00	1.0E+00	1.0E+00	1.0E+00	1.0E+00	1.7E-01	3.6E-01	1.0E+00	1.0E+00
IPR008283::Peptidase M17, leucyl aminopeptidase, N-terminal	0	0	0	0	0	0	0	0	0	0	1	8.8E-02	3.1E-01	9.1E-01	1.0E+00	1.0E+00	1.0E+00	1.0E+00	1.0E+00	1.7E-01	3.6E-01	8.3E-01	1.0E+00
IPR009090::D-aminopeptidase, middle/C-terminal	0	0	1	0	0	0	0	0	0	0	0	8.8E-02	3.1E-01	9.1E-01	1.0E+00	1.0E+00	1.0E+00	1.0E+00	1.0E+00	1.7E-01	3.6E-01	8.3E-01	1.0E+00
IPR011356::Peptidase M17	0	0	0	0	0	0	0	0	0	0	1	8.8E-02	3.1E-01	9.1E-01	1.0E+00	1.0E+00	1.0E+00	1.0E+00	1.0E+00	1.7E-01	3.6E-01	8.3E-01	1.0E+00
IPR011697::Peptidase C26	0	0	0	0	0	0	0	0	0	0	1	8.8E-02	3.1E-01	9.1E-01	1.0E+00	1.0E+00	1.0E+00	1.0E+00	1.0E+00	1.7E-01	3.6E-01	8.3E-01	1.0E+00
IPR012857::Peptidase S12, aminopeptidase DmpB, domain C	0	0	1	0	0	0	0	0	0	0	0	8.8E-02	3.1E-01	9.1E-01	1.0E+00	1.0E+00	1.0E+00	1.0E+00	1.0E+00	1.7E-01	3.6E-01	8.3E-01	1.0E+00
IPR022229::Peptidase S8A, tripeptidyl peptidase II	0	0	0	0	0	0	0	0	0	0	1	8.8E-02	3.1E-01	9.1E-01	1.0E+00	1.0E+00	1.0E+00	1.0E+00	1.0E+00	1.7E-01	3.6E-01	8.3E-01	1.0E+00
IPR002933::Peptidase M20	12	14	15	12	6	5	5	4	6	3	3	9.8E-02	3.1E-01	9.7E-01	1.0E+00	1.7E-01	1.0E+00	9.7E-01	1.0E+00	3.2E-01	3.6E-01	8.4E-01	1.0E+00
IPR011650::Peptidase M20, dimerisation	12	14	16	12	7	5	6	5	6	3	3	1.2E-01	3.1E-01	9.6E-01	1.0E+00	2.4E-01	1.0E+00	9.5E-01	1.0E+00	3.9E-01	3.6E-01	7.9E-01	1.0E+00
IPR000180::Peptidase M19, renal dipeptidase, active site	0	0	0	0	2	0	0	0	0	0	0	1.7E-01	3.1E-01	8.3E-01	1.0E+00	7.7E-01	1.0E+00	2.3E-01	1.0E+00	3.0E-01	3.6E-01	7.0E-01	1.0E+00
IPR000668::Peptidase C1A, papain C-terminal	0	0	0	0	0	0	1	1	0	0	0	1.7E-01	3.1E-01	8.3E-01	1.0E+00	1.0E+00	1.0E+00	1.0E+00	1.0E+00	3.0E-01	3.6E-01	7.0E-01	1.0E+00
IPR005321::Peptidase S58, DmpA	0	1	0	1	0	0	0	0	0	0	0	1.7E-01	3.1E-01	8.3E-01	1.0E+00	1.0E+00	1.0E+00	1.0E+00	1.0E+00	3.0E-01	3.6E-01	7.0E-01	1.0E+00
IPR005945::Peptidase S33, tricorn interacting factor 1	0	0	1	0	0	0	0	1	0	0	0	1.7E-01	3.1E-01	8.3E-01	1.0E+00	1.0E+00	1.0E+00	1.0E+00	1.0E+00	3.0E-01	3.6E-01	7.0E-01	1.0E+00
IPR006026::Peptidase, metallopeptidase	0	0	2	0	0	0	0	0	0	0	0	1.7E-01	3.1E-01	8.3E-01	1.0E+00	1.0E+00	1.0E+00	1.0E+00	1.0E+00	3.0E-01	3.6E-01	7.0E-01	1.0E+00
IPR007921::Cysteine, histidine-dependent amidohydrolase/peptidase	0	0	0	0	0	0	1	1	0	0	0	1.7E-01	3.1E-01	8.3E-01	1.0E+00	1.0E+00	1.0E+00	1.0E+00	1.0E+00	3.0E-01	3.6E-01	7.0E-01	1.0E+00
IPR012962::Peptidase M54, archaemetzincin	0	1	0	0	0	0	0	1	0	0	0	1.7E-01	3.1E-01	8.3E-01	1.0E+00	1.0E+00	1.0E+00	1.0E+00	1.0E+00	3.0E-01	3.6E-01	7.0E-01	1.0E+00
IPR012985::Peptidase S64, Ssy5	0	0	0	0	0	0	0	0	0	1	0	1.7E-01	3.1E-01	8.3E-01	1.0E+00	1.0E+00	1.0E+00	1.0E+00	1.0E+00	3.0E-01	3.6E-01	7.0E-01	1.0E+00
IPR013128::Peptidase C1A, papain	0	0	0	0	0	0	1	1	0	0	0	1.7E-01	3.1E-01	8.3E-01	1.0E+00	1.0E+00	1.0E+00	1.0E+00	1.0E+00	3.0E-01	3.6E-01	7.0E-01	1.0E+00
IPR013856::Peptidase M4, thermolysin	0	0	1	0	0	0	0	0	1	0	0	1.7E-01	3.1E-01	8.3E-01	1.0E+00	1.0E+00	1.0E+00	1.0E+00	1.0E+00	3.0E-01	3.6E-01	7.0E-01	1.0E+00
IPR010168::Peptidase M20D, amidohydrolase	4	4	6	4	2	0	2	2	2	0	0	1.9E-01	3.1E-01	9.8E-01	1.0E+00	3.9E-01	1.0E+00	9.8E-01	1.0E+00	6.5E-01	3.6E-01	7.4E-01	1.0E+00
IPR017439::Peptidase M20D, mername-AA028/carboxypeptidase Ss1	2	2	2	2	1	0	0	0	0	0	0	1.9E-01	3.1E-01	9.9E-01	1.0E+00	5.4E-01	1.0E+00	1.0E+00	1.0E+00	4.6E-01	3.6E-01	9.5E-01	1.0E+00
IPR008256::Peptidase S1B, glutamyl endopeptidase I	0	1	0	0	1	1	0	0	0	0	0	2.4E-01	3.1E-01	7.6E-01	1.0E+00	5.3E-01	1.0E+00	4.7E-01	1.0E+00	4.2E-01	3.6E-01	1.0E+00	1.0E+00
IPR008757::Peptidase M6-like, domain	0	0	0	0	1	1	0	1	0	0	0	2.4E-01	3.1E-01	7.6E-01	1.0E+00	5.3E-01	1.0E+00	4.7E-01	1.0E+00	4.2E-01	3.6E-01	1.0E+00	1.0E+00
IPR010435::Peptidase S8A, DUF1034 C-terminal	0	0	0	0	0	0	1	1	1	0	0	2.4E-01	3.1E-01	7.6E-01	1.0E+00	1.0E+00	1.0E+00	1.0E+00	1.0E+00	4.2E-01	3.6E-01	5.8E-01	1.0E+00
IPR019533::Peptidase S26, conserved region	1	0	0	0	0	0	1	1	0	0	0	2.4E-01	3.1E-01	1.0E+00	1.0E+00	5.3E-01	1.0E+00	1.0E+00	1.0E+00	4.2E-01	3.6E-01	1.0E+00	1.0E+00
IPR000169::Peptidase, cysteine peptidase active site	2	1	2	0	0	0	1	1	2	1	0	2.5E-01	3.1E-01	9.9E-01	1.0E+00	2.8E-01	1.0E+00	1.0E+00	1.0E+00	5.7E-01	3.6E-01	9.1E-01	1.0E+00
IPR000383::Peptidase S9/S15	3	4	7	1	0	0	1	2	2	0	0	2.6E-01	3.1E-01	9.7E-01	1.0E+00	1.5E-01	1.0E+00	1.0E+00	1.0E+00	6.7E-01	3.6E-01	7.7E-01	1.0E+00
IPR000718::Peptidase M13, neprilysin	1	1	0	1	0	0	0	1	0	0	0	3.1E-01	3.1E-01	1.0E+00	1.0E+00	5.3E-01	1.0E+00	1.0E+00	1.0E+00	5.2E-01	3.6E-01	9.8E-01	1.0E+00
IPR002870::Peptidase M12B, propeptide	1	0	1	0	0	0	1	1	0	0	0	3.1E-01	3.1E-01	1.0E+00	1.0E+00	5.3E-01	1.0E+00	1.0E+00	1.0E+00	5.2E-01	3.6E-01	9.8E-01	1.0E+00
IPR008753::Peptidase M13	1	1	0	1	0	0	0	1	0	0	0	3.1E-01	3.1E-01	1.0E+00	1.0E+00	5.3E-01	1.0E+00	1.0E+00	1.0E+00	5.2E-01	3.6E-01	9.8E-01	1.0E+00
IPR014766::Carboxypeptidase, regulatory domain	0	0	1	1	0	0	1	0	1	0	0	3.1E-01	3.1E-01	6.9E-01	1.0E+00	1.0E+00	1.0E+00	1.0E+00	1.0E+00	5.2E-01	3.6E-01	4.8E-01	1.0E+00
IPR018114::Peptidase S1/S6, chymotrypsin/Hap, active site	0	1	0	0	1	1	0	0	1	0	0	3.1E-01	3.1E-01	6.9E-01	1.0E+00	5.3E-01	1.0E+00	4.7E-01	1.0E+00	5.2E-01	3.6E-01	9.8E-01	1.0E+00
IPR018497::Peptidase M13, neprilysin, C-terminal	1	1	0	1	0	0	0	1	0	0	0	3.1E-01	3.1E-01	1.0E+00	1.0E+00	5.3E-01	1.0E+00	1.0E+00	1.0E+00	5.2E-01	3.6E-01	9.8E-01	1.0E+00
IPR019758::Peptidase S26A, signal peptidase I, conserved site	0	1	0	0	0	0	0	0	0	2	0	3.1E-01	3.1E-01	6.9E-01	1.0E+00	1.0E+00	1.0E+00	1.0E+00	1.0E+00	5.2E-01	3.6E-01	4.8E-01	1.0E+00
IPR012338::Beta-lactamase-type transpeptidase fold	7	8	11	6	5	6	6	7	6	0	0	3.2E-01	3.1E-01	9.0E-01	1.0E+00	4.6E-01	1.0E+00	9.0E-01	1.0E+00	2.4E-01	3.6E-01	9.1E-01	1.0E+00
IPR002410::Peptidase S33, prolyl aminopeptidase	2	1	2	0	1	2	1	3	1	0	0	3.5E-01	3.1E-01	9.7E-01	1.0E+00	5.4E-01	1.0E+00	1.0E+00	1.0E+00	1.9E-01	3.6E-01	9.8E-01	1.0E+00
IPR000816::Peptidase C15, pyroglutamyl peptidase I	1	1	0	0	1	1	1	0	0	0	0	3.7E-01	3.1E-01	9.9E-01	1.0E+00	7.7E-01	1.0E+00	1.0E+00	1.0E+00	2.0E-01	3.6E-01	1.0E+00	1.0E+00
IPR001570::Peptidase M4, thermolysin C-terminal	0	0	1	1	0	2	0	0	1	0	0	3.7E-01	3.1E-01	6.3E-01	1.0E+00	1.0E+00	1.0E+00	1.0E+00	1.0E+00	2.0E-01	3.6E-01	1.0E+00	1.0E+00
IPR001995::Peptidase A2A, retrovirus, catalytic	1	2	0	0	0	0	0	0	1	1	0	3.7E-01	3.1E-01	9.9E-01	1.0E+00	5.3E-01	1.0E+00	1.0E+00	1.0E+00	6.0E-01	3.6E-01	9.6E-01	1.0E+00
IPR017860::Peptidase M22, glycoprotease, conserved site	2	1	1	1	1	1	2	1	1	2	1	4.2E-01	3.1E-01	9.5E-01	1.0E+00	5.4E-01	1.0E+00	1.0E+00	1.0E+00	5.1E-01	3.6E-01	8.9E-01	1.0E+00
IPR001563::Peptidase S10, serine carboxypeptidase	8	5	12	11	10	11	5	5	4	3	3	4.3E-01	3.1E-01	8.2E-01	1.0E+00	8.2E-01	1.0E+00	5.1E-01	1.0E+00	7.0E-02	3.6E-01	9.8E-01	1.0E+00
IPR003146::Proteinase inhibitor, carboxypeptidase propeptide	0	0	0	0	2	2	1	0	1	0	0	4.3E-01	3.1E-01	5.7E-01	1.0E+00	7.7E-01	1.0E+00	2.3E-01	1.0E+00	2.6E-01	3.6E-01	9.9E-01	1.0E+00
IPR021860::Peptidase S12, Pab87-related, C-terminal	0	0	3	0	0	2	1	0	0	0	0	4.3E-01	3.1E-01	5.7E-01	1.0E+00	1.0E+00	1.0E+00	1.0E+00	1.0E+00	2.6E-01	3.6E-01	9.9E-01	1.0E+00
IPR005317::Peptidase M49, dipeptidyl-peptidase III	2	3	2	2	1	1	1	2	1	1	0	4.5E-01	3.1E-01	9.4E-01	1.0E+00	5.4E-01	1.0E+00	1.0E+00	1.0E+00	5.5E-01	3.6E-01	8.6E-01	1.0E+00
IPR013595::Peptidase S33, tripeptidyl-peptidase C-terminal	2	2	4	1	0	1	3	3	1	0	0	4.5E-01	3.1E-01	9.4E-01	1.0E+00	2.8E-01	1.0E+00	1.0E+00	1.0E+00	5.5E-01	3.6E-01	8.6E-01	1.0E+00
IPR013736::Peptidase S15/CocE/NonD, C-terminal	2	3	6	1	0	0	1	2	2	0	0	4.5E-01	3.1E-01	9.4E-01	1.0E+00	2.8E-01	1.0E+00	1.0E+00	1.0E+00	8.0E-01	3.8E-01	6.9E-01	1.0E+00
IPR017144::Peptidase M20D, amidohydrolase, predicted	2	2	4	2	1	0	2	2	2	0	0	4.5E-01	3.1E-01	9.4E-01	1.0E+00	5.4E-01	1.0E+00	1.0E+00	1.0E+00	8.0E-01	3.8E-01	6.9E-01	1.0E+00
IPR002471::Peptidase S9, serine active site	0	0	0	0	1	2	0	0	1	1	1	4.8E-01	3.1E-01	5.2E-01	1.0E+00	5.3E-01	1.0E+00	4.7E-01	1.0E+00	3.3E-01	3.6E-01	9.8E-01	1.0E+00
IPR004134::Peptidase C1B, bleomycin hydrolase	1	1	1	0	0	0	1	0	1	1	0	4.8E-01	3.1E-01	9.8E-01	1.0E+00	5.3E-01	1.0E+00	1.0E+00	1.0E+00	7.2E-01	3.6E-01	9.1E-01	1.0E+00
IPR005151::Peptidase S41	1	0	0	1	1	3	0	0	1	0	0	4.8E-01	3.1E-01	9.8E-01	1.0E+00	7.7E-01	1.0E+00	1.0E+00	1.0E+00	1.7E-02	3.6E-01	1.0E+00	1.0E+00
IPR018349::Peptidase M24A, methionine aminopeptidase, subfamily 2, binding site	2	2	2	2	2	2	1	1	1	1	1	4.8E-01	3.1E-01	9.3E-01	1.0E+00	7.2E-01	1.0E+00	9.2E-01	1.0E+00	3.5E-01	3.6E-01	9.4E-01	1.0E+00
IPR019757::Peptidase S26A, signal peptidase I, lysine active site	0	0	0	0	1	0	1	1	1	1	1	4.8E-01	3.1E-01	5.2E-01	1.0E+00	5.3E-01	1.0E+00	4.7E-01	1.0E+00	7.2E-01	3.6E-01	2.8E-01	1.0E+00
IPR003111::Peptidase S16, lon N-terminal	3	3	3	3	3	4	2	2	2	1	2	5.0E-01	3.1E-01	8.8E-01	1.0E+00	7.0E-01	1.0E+00	8.6E-01	1.0E+00	2.2E-01	3.6E-01	9.5E-01	1.0E+00
IPR019400::Peptidase C65, otubain	1	1	0	1	1	1	1	1	1	0	0	5.2E-01	3.1E-01	9.7E-01	1.0E+00	7.7E-01	1.0E+00	1.0E+00	1.0E+00	3.9E-01	3.6E-01	9.7E-01	1.0E+00
IPR001578::Peptidase C12, ubiquitin carboxyl-terminal hydrolase 1	4	4	5	4	4	5	4	5	3	1	2	5.3E-01	3.1E-01	8.2E-01	1.0E+00	6.9E-01	1.0E+00	8.2E-01	1.0E+00	2.8E-01	3.6E-01	9.1E-01	1.0E+00
IPR018202::Peptidase S10, serine carboxypeptidase, active site	6	5	10	9	8	9	3	4	4	3	2	5.5E-01	3.1E-01	7.6E-01	1.0E+00	8.4E-01	1.0E+00	5.3E-01	1.0E+00	1.4E-01	3.6E-01	9.6E-01	1.0E+00
IPR001907::Peptidase S14, ClpP	1	1	1	1	1	1	1	1	1	0	0	5.6E-01	3.1E-01	9.6E-01	1.0E+00	7.7E-01	1.0E+00	1.0E+00	1.0E+00	4.6E-01	3.6E-01	9.5E-01	1.0E+00
IPR002468::Peptidase M24A, methionine aminopeptidase, subfamily 2	2	2	2	3	2	2	2	2	1	1	1	5.6E-01	3.1E-01	8.9E-01	1.0E+00	7.2E-01	1.0E+00	9.2E-01	1.0E+00	4.7E-01	3.6E-01	8.8E-01	1.0E+00
IPR004815::Peptidase S16, ATP-dependent protease La	2	2	2	2	2	2	2	2	2	1	1	5.6E-01	3.1E-01	8.9E-01	1.0E+00	7.2E-01	1.0E+00	9.2E-01	1.0E+00	4.7E-01	3.6E-01	8.8E-01	1.0E+00
IPR011096::Propeptide, peptidase M4/M36	0	0	1	1	2	5	0	0	0	0	0	5.6E-01	3.1E-01	4.4E-01	1.0E+00	7.7E-01	1.0E+00	2.3E-01	1.0E+00	8.8E-03	3.6E-01	1.0E+00	1.0E+00
IPR016125::Peptidase C15, pyroglutamyl peptidase I-like	1	1	1	1	1	1	1	1	1	0	0	5.6E-01	3.1E-01	9.6E-01	1.0E+00	7.7E-01	1.0E+00	1.0E+00	1.0E+00	4.6E-01	3.6E-01	9.5E-01	1.0E+00
IPR018215::Peptidase S14, ClpP, active site	1	1	1	1	1	1	1	1	1	0	0	5.6E-01	3.1E-01	9.6E-01	1.0E+00	7.7E-01	1.0E+00	1.0E+00	1.0E+00	4.6E-01	3.6E-01	9.5E-01	1.0E+00
IPR019103::Peptidase aspartic, eukaryotic predicted	0	1	1	1	1	1	0	0	1	1	1	5.6E-01	3.1E-01	4.4E-01	1.0E+00	5.3E-01	1.0E+00	4.7E-01	1.0E+00	8.0E-01	3.8E-01	8.2E-01	1.0E+00
IPR022683::Peptidase C2, calpain, domain III	1	1	1	1	1	1	1	1	1	0	0	5.6E-01	3.1E-01	9.6E-01	1.0E+00	7.7E-01	1.0E+00	1.0E+00	1.0E+00	4.6E-01	3.6E-01	9.5E-01	1.0E+00
IPR000223::Peptidase S26A, signal peptidase I	2	2	2	2	2	1	2	1	2	2	2	5.9E-01	3.1E-01	8.8E-01	1.0E+00	7.2E-01	1.0E+00	9.2E-01	1.0E+00	7.3E-01	3.6E-01	7.0E-01	1.0E+00
IPR000246::Peptidase T2, asparaginase 2	2	2	1	2	2	2	3	3	3	0	1	5.9E-01	3.1E-01	8.8E-01	1.0E+00	7.2E-01	1.0E+00	9.2E-01	1.0E+00	5.1E-01	3.6E-01	8.5E-01	1.0E+00
IPR001394::Peptidase C19, ubiquitin carboxyl-terminal hydrolase 2	17	17	17	17	18	17	15	15	15	18	16	5.9E-01	3.1E-01	6.1E-01	1.0E+00	7.4E-01	1.0E+00	5.1E-01	1.0E+00	4.4E-01	3.6E-01	7.0E-01	1.0E+00
IPR001915::Peptidase M48	2	2	2	2	1	1	2	2	2	2	2	5.9E-01	3.1E-01	8.8E-01	1.0E+00	5.4E-01	1.0E+00	1.0E+00	1.0E+00	7.3E-01	3.6E-01	7.0E-01	1.0E+00
IPR001930::Peptidase M1, Alanine aminopeptidase/Leukotriene A4 hydrolase	3	3	3	2	3	2	3	3	3	4	2	5.9E-01	3.1E-01	8.2E-01	1.0E+00	7.0E-01	1.0E+00	8.6E-01	1.0E+00	6.9E-01	3.6E-01	6.7E-01	1.0E+00
IPR008269::Peptidase S16, Lon C-terminal	2	2	2	2	2	2	2	2	2	1	1	5.9E-01	3.1E-01	8.8E-01	1.0E+00	7.2E-01	1.0E+00	9.2E-01	1.0E+00	5.1E-01	3.6E-01	8.5E-01	1.0E+00
IPR008758::Peptidase S28	2	2	3	3	3	2	3	2	2	0	0	5.9E-01	3.1E-01	8.8E-01	1.0E+00	8.4E-01	1.0E+00	7.8E-01	1.0E+00	5.1E-01	3.6E-01	8.5E-01	1.0E+00
IPR001254::Peptidase S1/S6, chymotrypsin/Hap	0	2	1	1	2	2	0	0	1	0	0	6.0E-01	3.1E-01	4.0E-01	1.0E+00	7.7E-01	1.0E+00	2.3E-01	1.0E+00	5.1E-01	3.6E-01	9.3E-01	1.0E+00
IPR001842::Peptidase M36, fungalysin	0	0	1	1	2	6	0	0	0	0	0	6.0E-01	3.1E-01	4.0E-01	1.0E+00	7.7E-01	1.0E+00	2.3E-01	1.0E+00	2.4E-03	3.6E-01	1.0E+00	1.0E+00
IPR008442::Propeptide, carboxypeptidase Y	1	1	1	1	1	0	1	1	1	1	0	6.0E-01	3.1E-01	9.5E-01	1.0E+00	7.7E-01	1.0E+00	1.0E+00	1.0E+00	8.4E-01	3.9E-01	7.8E-01	1.0E+00
IPR012462::Peptidase C78, ubiquitin fold modifier-specific peptidase 1/ 2	1	1	1	1	1	1	1	1	1	0	1	6.0E-01	3.1E-01	9.5E-01	1.0E+00	7.7E-01	1.0E+00	1.0E+00	1.0E+00	5.1E-01	3.6E-01	9.3E-01	1.0E+00
IPR015211::Peptidase M1, leukotriene A4 hydrolase, aminopeptidase C-terminal	1	1	1	0	1	0	1	1	1	1	1	6.0E-01	3.1E-01	9.5E-01	1.0E+00	7.7E-01	1.0E+00	1.0E+00	1.0E+00	8.4E-01	3.9E-01	7.8E-01	1.0E+00
IPR022682::Peptidase C2, calpain, large subunit, domain III	1	1	1	1	1	1	1	1	1	0	0	6.0E-01	3.1E-01	9.5E-01	1.0E+00	7.7E-01	1.0E+00	1.0E+00	1.0E+00	5.1E-01	3.6E-01	9.3E-01	1.0E+00
IPR022684::Peptidase C2, calpain family	1	2	1	1	1	1	1	1	1	0	0	6.0E-01	3.1E-01	9.5E-01	1.0E+00	7.7E-01	1.0E+00	1.0E+00	1.0E+00	5.1E-01	3.6E-01	9.3E-01	1.0E+00
IPR000243::Peptidase T1A, proteasome beta-subunit	3	3	3	3	3	3	3	3	3	3	2	6.1E-01	3.1E-01	8.1E-01	1.0E+00	7.0E-01	1.0E+00	8.6E-01	1.0E+00	5.4E-01	3.6E-01	7.8E-01	1.0E+00
IPR001714::Peptidase M24, methionine aminopeptidase	3	4	4	5	3	2	3	3	2	2	2	6.1E-01	3.1E-01	8.1E-01	1.0E+00	7.0E-01	1.0E+00	8.6E-01	1.0E+00	7.1E-01	3.6E-01	6.4E-01	1.0E+00
IPR001300::Peptidase C2, calpain, catalytic domain	2	2	2	2	1	1	3	4	4	1	0	6.2E-01	3.1E-01	8.6E-01	1.0E+00	5.4E-01	1.0E+00	1.0E+00	1.0E+00	7.6E-01	3.7E-01	6.7E-01	1.0E+00
IPR014782::Peptidase M1, membrane alanine aminopeptidase, N-terminal	3	3	3	2	3	2	3	3	3	4	3	6.3E-01	3.1E-01	7.9E-01	1.0E+00	7.0E-01	1.0E+00	8.6E-01	1.0E+00	7.4E-01	3.6E-01	6.1E-01	1.0E+00
IPR000905::Peptidase M22, glycoprotease	2	2	2	2	2	2	2	2	2	2	2	6.4E-01	3.1E-01	8.4E-01	1.0E+00	7.2E-01	1.0E+00	9.2E-01	1.0E+00	5.8E-01	3.6E-01	8.0E-01	1.0E+00
IPR001940::Peptidase S1C, HrtA/DegP2/Q/S	1	1	1	1	1	1	1	1	1	1	1	6.4E-01	3.1E-01	9.3E-01	1.0E+00	7.7E-01	1.0E+00	1.0E+00	1.0E+00	5.7E-01	3.6E-01	9.1E-01	1.0E+00
IPR002610::Peptidase S54, rhomboid	2	2	2	2	2	2	2	2	3	1	2	6.4E-01	3.1E-01	8.4E-01	1.0E+00	7.2E-01	1.0E+00	9.2E-01	1.0E+00	5.8E-01	3.6E-01	8.0E-01	1.0E+00
IPR006639::Peptidase A22, presenilin signal peptide	1	1	1	1	1	1	1	1	1	1	1	6.4E-01	3.1E-01	9.3E-01	1.0E+00	7.7E-01	1.0E+00	1.0E+00	1.0E+00	5.7E-01	3.6E-01	9.1E-01	1.0E+00
IPR007369::Peptidase A22B, signal peptide peptidase	1	1	1	1	1	1	1	1	1	1	1	6.4E-01	3.1E-01	9.3E-01	1.0E+00	7.7E-01	1.0E+00	1.0E+00	1.0E+00	5.7E-01	3.6E-01	9.1E-01	1.0E+00
IPR008754::Peptidase M43, pregnancy-associated plasma-A	1	2	1	1	1	2	1	0	2	0	0	6.4E-01	3.1E-01	9.3E-01	1.0E+00	7.7E-01	1.0E+00	1.0E+00	1.0E+00	2.7E-01	3.6E-01	9.8E-01	1.0E+00
IPR009542::Microsomal signal peptidase 12kDa subunit	1	1	1	1	1	1	1	1	1	1	1	6.4E-01	3.1E-01	9.3E-01	1.0E+00	7.7E-01	1.0E+00	1.0E+00	1.0E+00	5.7E-01	3.6E-01	9.1E-01	1.0E+00
IPR011600::Peptidase C14, caspase catalytic	2	4	3	2	1	2	2	2	3	1	1	6.4E-01	3.1E-01	8.4E-01	1.0E+00	5.4E-01	1.0E+00	1.0E+00	1.0E+00	5.8E-01	3.6E-01	8.0E-01	1.0E+00
IPR016295::Proteasome endopeptidase complex, beta subunit	1	1	1	1	1	1	0	1	1	1	1	6.4E-01	3.1E-01	9.3E-01	1.0E+00	7.7E-01	1.0E+00	1.0E+00	1.0E+00	5.7E-01	3.6E-01	9.1E-01	1.0E+00
IPR017861::Peptidase M22, glycoprotease, subgroup	2	2	2	2	2	2	2	2	2	2	2	6.4E-01	3.1E-01	8.4E-01	1.0E+00	7.2E-01	1.0E+00	9.2E-01	1.0E+00	5.8E-01	3.6E-01	8.0E-01	1.0E+00
IPR015927::Peptidase S24/S26A/S26B/S26C	3	3	3	3	4	3	3	3	3	3	3	6.5E-01	3.1E-01	7.7E-01	1.0E+00	8.1E-01	1.0E+00	7.3E-01	1.0E+00	5.9E-01	3.6E-01	7.4E-01	1.0E+00
IPR022764::Peptidase S54, rhomboid domain	3	3	3	3	3	3	3	3	4	2	4	6.5E-01	3.1E-01	7.7E-01	1.0E+00	7.0E-01	1.0E+00	8.6E-01	1.0E+00	5.9E-01	3.6E-01	7.4E-01	1.0E+00
IPR001567::Peptidase M3A/M3B, thimet/oligopeptidase F	2	1	4	2	2	2	2	3	2	2	1	6.6E-01	3.1E-01	8.3E-01	1.0E+00	7.2E-01	1.0E+00	9.2E-01	1.0E+00	6.2E-01	3.6E-01	7.7E-01	1.0E+00
IPR001948::Peptidase M18	2	3	2	2	2	2	2	2	2	2	1	6.6E-01	3.1E-01	8.3E-01	1.0E+00	7.2E-01	1.0E+00	9.2E-01	1.0E+00	6.2E-01	3.6E-01	7.7E-01	1.0E+00
IPR018200::Peptidase C19, ubiquitin carboxyl-terminal hydrolase 2, conserved site	12	11	11	12	14	13	11	10	11	16	14	6.6E-01	3.1E-01	5.6E-01	1.0E+00	8.0E-01	1.0E+00	4.7E-01	1.0E+00	5.0E-01	3.6E-01	6.7E-01	1.0E+00
IPR001096::Peptidase C13, legumain	1	1	1	1	1	1	1	1	1	1	1	6.7E-01	3.1E-01	9.2E-01	1.0E+00	7.7E-01	1.0E+00	1.0E+00	1.0E+00	6.2E-01	3.6E-01	8.8E-01	1.0E+00
IPR001590::Peptidase M12B, ADAM/reprolysin	1	1	2	2	1	1	1	1	1	0	1	6.7E-01	3.1E-01	9.2E-01	1.0E+00	7.7E-01	1.0E+00	1.0E+00	1.0E+00	6.2E-01	3.6E-01	8.8E-01	1.0E+00
IPR005078::Peptidase C54	1	1	1	1	1	1	1	1	1	1	1	6.7E-01	3.1E-01	9.2E-01	1.0E+00	7.7E-01	1.0E+00	1.0E+00	1.0E+00	6.2E-01	3.6E-01	8.8E-01	1.0E+00
IPR005314::Peptidase C50, separase	1	1	1	1	1	1	1	1	1	1	1	6.7E-01	3.1E-01	9.2E-01	1.0E+00	7.7E-01	1.0E+00	1.0E+00	1.0E+00	6.2E-01	3.6E-01	8.8E-01	1.0E+00
IPR007653::Signal peptidase 22kDa subunit	1	1	1	1	1	1	1	1	1	1	1	6.7E-01	3.1E-01	9.2E-01	1.0E+00	7.7E-01	1.0E+00	1.0E+00	1.0E+00	6.2E-01	3.6E-01	8.8E-01	1.0E+00
IPR007863::Peptidase M16, C-terminal	6	6	7	6	6	6	6	6	6	8	6	6.7E-01	3.1E-01	6.4E-01	1.0E+00	6.8E-01	1.0E+00	7.5E-01	1.0E+00	6.2E-01	3.6E-01	6.2E-01	1.0E+00
IPR008268::Peptidase S16, active site	1	1	1	1	1	1	1	1	1	1	1	6.7E-01	3.1E-01	9.2E-01	1.0E+00	7.7E-01	1.0E+00	1.0E+00	1.0E+00	6.2E-01	3.6E-01	8.8E-01	1.0E+00
IPR009582::Signal peptidase complex subunit 2	1	1	1	1	1	1	1	1	1	1	1	6.7E-01	3.1E-01	9.2E-01	1.0E+00	7.7E-01	1.0E+00	1.0E+00	1.0E+00	6.2E-01	3.6E-01	8.8E-01	1.0E+00
IPR011765::Peptidase M16, N-terminal	6	6	7	6	5	6	6	6	6	8	6	6.7E-01	3.1E-01	6.4E-01	1.0E+00	5.7E-01	1.0E+00	8.5E-01	1.0E+00	6.2E-01	3.6E-01	6.2E-01	1.0E+00
IPR013578::Peptidase M16C associated	1	1	1	1	1	1	1	1	1	1	1	6.7E-01	3.1E-01	9.2E-01	1.0E+00	7.7E-01	1.0E+00	1.0E+00	1.0E+00	6.2E-01	3.6E-01	8.8E-01	1.0E+00
IPR016526::Peptidase M49, dipeptidyl-peptidase 3, eukaryotic	1	1	1	2	1	1	1	1	1	1	0	6.7E-01	3.1E-01	9.2E-01	1.0E+00	7.7E-01	1.0E+00	1.0E+00	1.0E+00	6.2E-01	3.6E-01	8.8E-01	1.0E+00
IPR017141::Peptidase M20, carboxypeptidase S	1	2	1	1	0	0	1	1	1	1	1	6.7E-01	3.1E-01	9.2E-01	1.0E+00	5.3E-01	1.0E+00	1.0E+00	1.0E+00	8.9E-01	4.0E-01	6.8E-01	1.0E+00
IPR019165::Peptidase M76, ATP23	1	1	1	1	1	1	1	1	1	1	1	6.7E-01	3.1E-01	9.2E-01	1.0E+00	7.7E-01	1.0E+00	1.0E+00	1.0E+00	6.2E-01	3.6E-01	8.8E-01	1.0E+00
IPR022495::Kinase-associated endopeptidase 1, Bud32	1	1	1	1	1	1	1	1	1	1	1	6.7E-01	3.1E-01	9.2E-01	1.0E+00	7.7E-01	1.0E+00	1.0E+00	1.0E+00	6.2E-01	3.6E-01	8.8E-01	1.0E+00
IPR000101::Gamma-glutamyltranspeptidase	2	2	4	2	2	3	2	2	2	1	2	6.8E-01	3.1E-01	8.1E-01	1.0E+00	7.2E-01	1.0E+00	9.2E-01	1.0E+00	4.4E-01	3.6E-01	8.7E-01	1.0E+00
IPR000642::Peptidase M41	2	2	2	2	2	2	2	2	2	3	2	6.8E-01	3.1E-01	8.1E-01	1.0E+00	7.2E-01	1.0E+00	9.2E-01	1.0E+00	6.5E-01	3.6E-01	7.4E-01	1.0E+00
IPR001984::Peptidase S16, Lon protease, C-terminal	2	2	2	2	2	2	3	2	4	1	1	6.8E-01	3.1E-01	8.1E-01	1.0E+00	7.2E-01	1.0E+00	9.2E-01	1.0E+00	6.5E-01	3.6E-01	7.4E-01	1.0E+00
IPR005936::Peptidase M41, FtsH	2	2	2	2	2	2	2	2	2	3	2	6.8E-01	3.1E-01	8.1E-01	1.0E+00	7.2E-01	1.0E+00	9.2E-01	1.0E+00	6.5E-01	3.6E-01	7.4E-01	1.0E+00
IPR011237::Peptidase M16, core	6	6	7	6	6	6	6	6	6	8	6	6.8E-01	3.1E-01	6.3E-01	1.0E+00	6.8E-01	1.0E+00	7.5E-01	1.0E+00	6.4E-01	3.6E-01	6.0E-01	1.0E+00
IPR001431::Peptidase M16, zinc-binding site	2	2	2	2	2	2	2	2	2	5	3	7.0E-01	3.1E-01	7.9E-01	1.0E+00	7.2E-01	1.0E+00	9.2E-01	1.0E+00	6.8E-01	3.6E-01	7.1E-01	1.0E+00
IPR001733::Peptidase S26B, eukaryotic signal peptidase	1	1	1	1	2	1	1	1	1	1	1	7.0E-01	3.1E-01	9.0E-01	1.0E+00	8.9E-01	1.0E+00	8.5E-01	1.0E+00	6.6E-01	3.6E-01	8.4E-01	1.0E+00
IPR006615::Peptidase C19, ubiquitin-specific peptidase, DUSP domain	1	1	1	1	1	1	1	1	1	1	2	7.0E-01	3.1E-01	9.0E-01	1.0E+00	7.7E-01	1.0E+00	1.0E+00	1.0E+00	6.6E-01	3.6E-01	8.4E-01	1.0E+00
IPR021917::Uncharacterised protein family, zinc metallopeptidase-like	1	1	1	1	1	1	2	1	1	1	1	7.0E-01	3.1E-01	9.0E-01	1.0E+00	7.7E-01	1.0E+00	1.0E+00	1.0E+00	6.6E-01	3.6E-01	8.4E-01	1.0E+00
IPR011249::Metalloenzyme, LuxS/M16 peptidase-like, metal-binding	6	6	7	7	6	6	7	6	6	8	6	7.1E-01	3.1E-01	6.0E-01	1.0E+00	6.8E-01	1.0E+00	7.5E-01	1.0E+00	6.8E-01	3.6E-01	5.6E-01	1.0E+00
IPR003653::Peptidase C48, SUMO/Sentrin/Ubl1	3	4	3	3	6	3	3	4	4	2	4	7.3E-01	3.1E-01	6.9E-01	1.0E+00	9.3E-01	1.0E+00	4.4E-01	1.0E+00	7.2E-01	3.6E-01	6.0E-01	1.0E+00
IPR011546::Peptidase M41, FtsH extracellular	1	1	1	1	1	1	1	1	1	2	1	7.3E-01	3.1E-01	8.8E-01	1.0E+00	7.7E-01	1.0E+00	1.0E+00	1.0E+00	7.0E-01	3.6E-01	8.1E-01	1.0E+00
IPR016117::Peptidase S58 DmpA/arginine biosynthesis protein ArgJ	1	2	1	2	1	1	1	1	1	1	1	7.3E-01	3.1E-01	8.8E-01	1.0E+00	7.7E-01	1.0E+00	1.0E+00	1.0E+00	7.0E-01	3.6E-01	8.1E-01	1.0E+00
IPR000250::Peptidase G1, eqolisin	0	2	4	2	0	0	2	4	1	0	0	7.5E-01	3.1E-01	2.5E-01	1.0E+00	1.0E+00	1.0E+00	1.0E+00	1.0E+00	9.3E-01	4.2E-01	6.6E-02	1.0E+00

IPR007484::Peptidase M28	6	7	7	7	9	11	9	7	8	5	3	7.6E-01	3.1E-01	5.3E-01	1.0E+00	8.9E-01	1.0E+00	4.2E-01	1.0E+00	2.2E-01	3.6E-01	9.1E-01	1.0E+00
IPR002467::Peptidase M24A, methionine aminopeptidase, subfamily 1	1	3	2	2	1	1	1	1	1	1	1	7.7E-01	3.1E-01	8.4E-01	1.0E+00	7.7E-01	1.0E+00	1.0E+00	1.0E+00	7.7E-01	3.7E-01	7.3E-01	1.0E+00
IPR007230::Peptidase S59, nucleoporin	1	1	1	1	1	1	1	1	1	3	2	7.7E-01	3.1E-01	8.4E-01	1.0E+00	7.7E-01	1.0E+00	1.0E+00	1.0E+00	7.7E-01	3.7E-01	7.3E-01	1.0E+00
IPR007865::Peptidase M24B, X-Pro dipeptidase/aminopeptidase P N-terminal	2	3	3	3	3	3	3	3	3	2	1	7.7E-01	3.1E-01	7.1E-01	1.0E+00	8.4E-01	1.0E+00	7.8E-01	1.0E+00	6.0E-01	3.6E-01	7.5E-01	1.0E+00
IPR009007::Peptidase aspartic, catalytic	11	10	14	8	6	9	18	26	19	10	2	7.7E-01	3.1E-01	4.4E-01	1.0E+00	2.2E-01	1.0E+00	9.6E-01	1.0E+00	8.8E-01	4.0E-01	2.4E-01	1.0E+00
IPR019756::Peptidase S26A, signal peptidase I, serine active site	1	1	1	1	2	1	1	1	1	3	0	7.7E-01	3.1E-01	8.4E-01	1.0E+00	8.9E-01	1.0E+00	8.5E-01	1.0E+00	7.7E-01	3.7E-01	7.3E-01	1.0E+00
IPR000994::Peptidase M24, structural domain	8	12	12	11	9	9	9	9	9	7	8	7.8E-01	3.1E-01	4.7E-01	1.0E+00	7.6E-01	1.0E+00	6.1E-01	1.0E+00	7.0E-01	3.6E-01	5.0E-01	1.0E+00
IPR001375::Peptidase S9, prolyl oligopeptidase, catalytic domain	4	10	9	6	6	5	4	7	3	2	3	8.0E-01	3.2E-01	5.5E-01	1.0E+00	8.7E-01	1.0E+00	5.6E-01	1.0E+00	7.0E-01	3.6E-01	5.7E-01	1.0E+00
IPR001461::Peptidase A1	10	10	13	7	6	8	18	24	18	9	2	8.0E-01	3.2E-01	4.1E-01	1.0E+00	2.9E-01	1.0E+00	9.4E-01	1.0E+00	9.1E-01	4.1E-01	2.0E-01	1.0E+00
IPR019759::Peptidase S24/S26A/S26B, conserved region	2	3	3	3	4	2	3	2	3	3	3	8.1E-01	3.2E-01	6.5E-01	1.0E+00	9.1E-01	1.0E+00	6.1E-01	1.0E+00	8.4E-01	3.9E-01	4.9E-01	1.0E+00
IPR021109::Peptidase aspartic	11	11	15	9	7	10	18	25	20	11	3	8.2E-01	3.2E-01	3.7E-01	1.0E+00	3.1E-01	1.0E+00	9.3E-01	1.0E+00	8.8E-01	4.0E-01	2.3E-01	1.0E+00
IPR002469::Peptidase S9B, dipeptidylpeptidase IV N-terminal	1	2	1	2	2	2	2	1	1	2	2	8.4E-01	3.3E-01	7.4E-01	1.0E+00	8.9E-01	1.0E+00	8.5E-01	1.0E+00	6.7E-01	3.6E-01	7.7E-01	1.0E+00
IPR011056::Peptidase S24/S26A/S26B/S26C, beta-ribbon domain	2	3	3	3	4	3	3	3	3	3	3	8.4E-01	3.3E-01	6.1E-01	1.0E+00	9.1E-01	1.0E+00	6.1E-01	1.0E+00	7.4E-01	3.6E-01	6.1E-01	1.0E+00
IPR001384::Peptidase M35, deuterolysin	0	4	0	3	7	5	0	0	2	0	0	8.6E-01	3.3E-01	1.4E-01	1.0E+00	9.9E-01	1.0E+00	5.4E-03	1.0E+00	2.6E-01	3.6E-01	9.5E-01	1.0E+00
IPR000834::Peptidase M14, carboxypeptidase A	1	2	1	1	3	4	3	2	2	1	1	8.7E-01	3.4E-01	6.9E-01	1.0E+00	9.5E-01	1.0E+00	6.5E-01	1.0E+00	3.0E-01	3.6E-01	9.4E-01	1.0E+00
IPR001131::Peptidase M24B, X-Pro dipeptidase/aminopeptidase P, conserved site	1	2	2	2	2	2	2	3	2	3	3	9.0E-01	3.5E-01	6.2E-01	1.0E+00	8.9E-01	1.0E+00	8.5E-01	1.0E+00	8.1E-01	3.8E-01	6.0E-01	1.0E+00
IPR009003::Serine/cysteine peptidase, trypsin-like	1	3	1	2	2	9	2	1	3	2	2	9.4E-01	3.6E-01	5.0E-01	1.0E+00	8.9E-01	1.0E+00	8.5E-01	1.0E+00	1.9E-02	3.6E-01	1.0E+00	1.0E+00
IPR001969::Peptidase aspartic, active site	7	5	7	5	4	5	9	16	11	34	14	9.5E-01	3.6E-01	1.7E-01	1.0E+00	3.3E-01	1.0E+00	9.5E-01	1.0E+00	9.9E-01	4.3E-01	2.1E-02	1.0E+00
IPR015366::Peptidase S53, propeptide	1	3	7	5	3	3	3	4	3	0	0	9.5E-01	3.6E-01	4.5E-01	1.0E+00	9.5E-01	1.0E+00	6.5E-01	1.0E+00	8.0E-01	3.8E-01	5.6E-01	1.0E+00
IPR015500::Peptidase S8, subtilisin-related	3	4	9	5	18	17	8	9	7	4	4	9.9E-01	3.7E-01	7.8E-02	1.0E+00	1.0E+00	1.0E+00	1.8E-03	1.0E+00	1.3E-01	3.6E-01	9.5E-01	1.0E+00
IPR022398::Peptidase S8/S53, subtilisin, active site	3	4	8	6	17	17	10	11	9	4	4	9.9E-01	3.7E-01	5.9E-02	1.0E+00	1.0E+00	1.0E+00	3.0E-03	1.0E+00	1.9E-01	3.6E-01	9.2E-01	1.0E+00
IPR000209::Peptidase S8/S53, subtilisin/kexin/sedolisin	3	7	16	10	21	19	13	14	11	4	4	1.0E+00	3.7E-01	1.0E-02	1.0E+00	1.0E+00	1.0E+00	3.2E-04	5.0E-01	4.5E-01	3.6E-01	7.2E-01	1.0E+00
IPR002142::Peptidase S49	0	0	0	0	0	0	0	0	0	0	0	1.0E+00	3.7E-01	1.0E+00	1.0E+00	1.0E+00	1.0E+00	1.0E+00	1.0E+00	1.0E+00	4.3E-01	1.0E+00	1.0E+00
IPR002470::Peptidase S9A, prolyl oligopeptidase	0	0	0	0	0	0	0	0	0	0	0	1.0E+00	3.7E-01	1.0E+00	1.0E+00	1.0E+00	1.0E+00	1.0E+00	1.0E+00	1.0E+00	4.3E-01	1.0E+00	1.0E+00
IPR004106::Peptidase S9A, oligopeptidase, N-terminal beta-propeller	0	0	0	0	0	0	0	0	0	0	0	1.0E+00	3.7E-01	1.0E+00	1.0E+00	1.0E+00	1.0E+00	1.0E+00	1.0E+00	1.0E+00	4.3E-01	1.0E+00	1.0E+00
IPR005322::Peptidase C69, dipeptidase A	0	0	0	0	0	0	0	0	0	0	0	1.0E+00	3.7E-01	1.0E+00	1.0E+00	1.0E+00	1.0E+00	1.0E+00	1.0E+00	1.0E+00	4.3E-01	1.0E+00	1.0E+00
IPR008915::Peptidase M50	0	0	0	0	0	0	0	0	0	0	0	1.0E+00	3.7E-01	1.0E+00	1.0E+00	1.0E+00	1.0E+00	1.0E+00	1.0E+00	1.0E+00	4.3E-01	1.0E+00	1.0E+00
