# Supplementary material for: Comparative Genomic and Transcriptomic Analysis of Wangiella dermatitidis, A Major Cause of Phaeohyphomycosis and a Model Black Yeast Human Pathogen
Source: G3 (Bethesda). 2014 Feb 4;4(4):561–78. doi: 10.1534/g3.113.009241 (PMC4059230; doi:10.1534/g3.113.009241)
Supplement: Supporting Information [file supp_g3.113.009241_TableS2.pdf]

Table S2 Cell wall genes in *W. dermatitidis* and other fungal genomes^.

Gene ID*	Number of genes in species											pH		Radiation		Gene symbol#	Gene description#
	W. dermatitidis	*A. niger*	*A. fumigatus*	*A. nidulans*	*C. immitis*	*T. rubrum*	*N. crassa*	*M. thermophila*	*T. terrestris*	*S. cerevisiae*	*S. pombe*	log_2 Fold**	P-value***	log_2 Fold**	P-value***		
**Chitin synthase**																	
HMPREF1120_06816&	2	2	2	2	2	2	2	2	2	2	1	1.75	7.17E-28	0.49	2.88E-04	CHS2	Class I chitin synthase
HMPREF1120_07981&	paralog&											0.38	2.19E-02	-1.73	1.68E-24	CHS1	Class II chitin synthase
HMPREF1120_06479	1	3	2	2	1	1	1	1	1	0	0	1.86	3.01E-32	0.43	1.35E-03	CHS3	Class III chitin synthase
HMPREF1120_07721	1	1	1	1	1	1	1	1	1	1	0	1.32	2.77E-17	-0.23	9.57E-02	CHS4	Class IV chitin synthase
HMPREF1120_08776	2	2	2	2	2	2	2	2	2	0	0	1.31	3.54E-17	-0.68	1.18E-07	CHS5	Class V chitin synthase
HMPREF1120_08777	paralog											1.48	1.82E-21	-0.65	3.85E-07	CHS7	Class VII chitin synthase
HMPREF1120_09115	1	1	1	1	1	1	1	1	1	0	0	0.04	8.82E-01	-0.34	1.22E-02	CHS6	Class VI chitin synthase
HMPREF1120_01791	1	0	0	0	0	0	0	0	0	0	0	-2.21	1.60E-41	-3.13	2.33E-119		Chitin synthase like
HMPREF1120_01790	1	0	0	0	0	0	0	0	0	0	0	-1.89	8.44E-30	-3.08	3.34E-118		UDP-N-acetylglucosamine 6-dehydrogenase
**Regulation of chitin synthase activity, by analogy to S. cerevisiae**																	
HMPREF1120_07720	1	1	1	1	1	1	1	1	1	1	0	1.73	2.89E-28	-0.39	3.43E-03	SKT5	Activator of Chs3p during vegetative growth
HMPREF1120_06335	1	1	1	1	1	1	1	1	1	0	1	1.55	9.08E-23	-0.1	5.03E-01		Similarity with ScSkt5, activator of Chs3
HMPREF1120_05528	1	1	1	1	1	1	1	1	1	0	1	-0.44	7.54E-03	-0.03	8.78E-01		Similarity with ScSkt5, activator of Chs3
HMPREF1120_05249	1	1	1	1	1	1	1	1	1	1	0	0.77	1.12E-06	-0.27	4.54E-02	BNI4	scaffold protein that tethers chitin synthase III (Chs3p) to the bud neck
HMPREF1120_05359	1	1	1	1	1	1	1	1	1	1	1	0.09	6.35E-01	-0.82	2.28E-10	ScCHS5	Similarity with ScChs5, component of exomer complex
HMPREF1120_01856	1	1	1	1	1	1	1	1	1	1	1	-0.14	4.40E-01	0.09	5.54E-01	ScCHS6	Similarity with ScChs6, component of exomer complex
HMPREF1120_00837	1	1	1	1	1	1	1	1	1	1	0	1.37	4.24E-18	0.47	4.40E-04		Similarity with export control protein ScChs7
HMPREF1120_03003	1	1	1	1	1	1	1	1	1	0	0	0.51	1.97E-03	0.38	7.36E-03	ScCHS7	Similarity with export control protein ScChs7
**Chitin modification**																	
HMPREF1120_08023	1	1	1	1	1	1	2	2	2	2	0	0.21	2.21E-01	-0.4	4.07E-03	Cda1/2	Chitin deacetylase
HMPREF1120_01911	1	1	1	1	1	1	1	1	1	0	0	1.63	5.46E-14	-0.87	1.72E-04		
**Chitin degradation**																	
HMPREF1120_03399	1	1	1	1	1	1	1	1	1	0	1	-0.24	1.60E-01	-0.73	3.68E-06	ChiA	GPI anchored class III chitinase
HMPREF1120_02334	1	1	1	1	1	1	1	1	1	0	0	-0.64	9.20E-05	-1.84	1.09E-24		Class III chitinase
HMPREF1120_06669	1	3	3	2	3	2	1	1	1	1	0	0.57	9.88E-04	0.41	7.23E-03	ChiB	Class V chitinase
HMPREF1120_03714	1	1	2	2	0	0	1	1	1	0	0	0.14	4.60E-01	0.48	9.28E-04		Class V chitinase
HMPREF1120_04557	2	0	0	0	0	0	0	0	0	0	0	0.73	3.60E-03	0.57	2.22E-04		Chitinase
HMPREF1120_07241	paralog											-2.9	1.56E-68	-0.93	9.28E-12		Chitinase
HMPREF1120_06035	2	2	1	1	2	2	1	1	1	0	0	-0.17	3.73E-01	0.25	1.06E-01	NagA	Extracellular N-acetyl-beta-glucosaminidase with a predicted role in chitin hydrolysis
HMPREF1120_06285	paralog											-0.04	8.30E-01	-0.49	2.87E-04	NagA	
AN4686	0	1	3	1	1	1	1	1	1	0	0					CsnA	Chitosanases (GH 75); similarity with A. fumigatus CsnC and *A. oryzae* CsnC
AN11051	0	1	1	1	0	0	0	0	0	0	0					CsnC	
**1,3-α-glucan synthesis and processing**																	
AN3307, AN5885	0	5	3	2	1	0	2	1	1	5	0					AgsB/A	Catalytic subunits of the 1,3-α-glucan synthase complex (GT5 and GH13)
AN3790,AN7349,AN9042	0	1	1	1	1	0	0	0	0	0	0					AgnB/C/D	Putative 1,3-α-glucanase family (GH 71); related to the S. pombe Agn1-family
AN1604	0	2	3	1	0	0	1	1	1	0	0					AgnE	
AN3308, AN4507,	0	2	3	3	1	0	1	1	0	0	0					AmyC/D	Amylase-like family (GH 13); similarity with A. fumigatus AmyA
HMPREF1120_08319	1	4	2	3	0	0	1	1	1	0	6	-0.03	8.84E-01	1.35	1.56E-24		
HMPREF1120_03460	1	1	1	1	1	1	2	1	0	0	0	-1.33	2.41E-16	1.74	1.85E-33		Putative amylase; similarity with H. capsulatum Amy1
**1,3-β-glucan synthesis and processing**																	
HMPREF1120_03476	1	1	1	1	1	1	1	1	1	1	4	1.03	3.01E-11	0.09	5.32E-01	FksA	Putative catalytic subunit 1,3-β-glucan synthase complex; ScFks1-like
HMPREF1120_04893	1	1	1	1	1	1	1	1	1	1	1	0.67	3.49E-05	0.95	1.94E-12	ScSMI1	Putative regulatory component 1,3-β-glucan synthesis; ScKnr4-like
HMPREF1120_09022	1	1	1	1	1	1	1	1	1	1	1	-0.14	1.34E-18	-1.14	2.92E-18	EngA	Endo-1,3-β-glucanase (GH 81-family); ScEng1-like
HMPREF1120_04506	1	1	1	1	0	1	0	0	0	1	1	-0.85	1.60E-06	-2.72	9.07E-95		Putative exo-1,3-β-glucanase family (GH 5); related to the ScExg1-family
HMPREF1120_06180	1	1	1	1	1	1	1	1	1	0	1	-0.23	1.91E-01	0.01	9.74E-01		
HMPREF1120_01556	2	1	3	1	1	1	4	4	4	0	0	1.23	4.73E-10	0.17	3.61E-01		Putative exo-1,3-β-glucanase family (GH 55); related to Coniothyrium minitans exo-1,3-glucanase (Cmg1)
HMPREF1120_05230	paralog											1.23	6.03E-08	-0.27	1.02E-01		
HMPREF1120_00547	2	1	1	1	0	0	2	1	1	0	0	3.14	9.48E-83	1.03	8.29E-16		Bgl2-family of putative 1,3-β-transglucosylases (GH 17) proposed to be involved in connecting the emerging 1,3-β-glucan chains to the existing b-glucan network through 1,6-β-linkages; related to A. fumigatus Bgt1-family
HMPREF1120_05209	paralog											1.68	2.06E-27	-0.5	1.29E-04		
HMPREF1120_06595	1	1	1	1	1	1	1	0	0	1	0	-1.88	4.33E-25	-1.51	4.66E-26		
HMPREF1120_08449	1	1	1	1	1	1	1	1	1	0	0	0.36	3.91E-02	-0.18	2.26E-01		
HMPREF1120_04141	1	1	1	1	1	1	0	0	0	1	1	-0.22	2.54E-01	0.79	2.77E-08		
HMPREF1120_03066	1	1	1	1	1	1	1	1	1	1	0	0.2	2.33E-01	-0.6	7.68E-06		
HMPREF1120_08078	1	0	0	0	0	0	0	0	0	0	0	6.64	1.85E-268	0.13	3.95E-01		
HMPREF1120_04931	1	1	1	1	1	1	1	1	1	1	0	2.73	2.65E-65	0.27	4.85E-02		Crh1-family of putative transglycosidases (GH 16);involved in crosslinking b-glucan and chitin; related to ScCrh-family
HMPREF1120_00627	1	1	1	1	1	1	1	1	1	0	0	0.08	6.89E-01	-0.62	9.59E-04		
HMPREF1120_07927	1	1	1	1	1	0	1	1	1	1	0	-0.42	1.77E-02	0.005	9.97E-01		
HMPREF1120_02703	1	1	1	0	1	1	1	1	1	1	2	0.09	6.58E-01	-0.05	7.77E-01		
HMPREF1120_07283	1	1	1	1	1	1	1	1	1	1	0	0.77	1.07E-06	0.08	5.72E-01		Gas-family of putative 1,3-β-transglucosylases (GH 72) proposed to be involved in connecting the emerging 1,3-β-glucan chains to the existing β-glucan network; related to A. fumigatus Gel-family
HMPREF1120_01763	1	1	1	1	1	1	2	2	2	1	2	1.96	3.15E-36	-0.02	9.28E-01		
HMPREF1120_03477	1	2	2	2	1	1	1	1	1	1	1	1.33	1.06E-17	0.11	4.56E-01		
HMPREF1120_01682	1	1	1	0	1	0	1	0	0	0	0	0.63	8.79E-05	0.28	3.88E-02	GelG	1,3-β-glucanosyltransferase
HMPREF1120_01649	1	1	1	1	1	1	1	1	1	1	2	0.63	7.07E-05	-0.48	4.40E-04	SunA	Sun family, involved in septation, possibly β-glucosidase activity; related to ScSun-family
HMPREF1120_06902	1	1	1	1	1	1	1	1	1	1	1	0.92	1.48E-07	-0.16	4.07E-01	SunB	
HMPREF1120_01614	1	1	1	1	1	1	0	0	0	1	2	1.5	1.52E-21	-0.55	2.19E-05	Kre6	Putative transglycosidase required for 1,6-β-glucan biosynthesis
HMPREF1120_04699	2	1	2	1	0	0	0	0	0	0	0	0.94	1.75E-09	0.47	3.56E-04	CelA	Similarity with cellulose synthases of the GT 2 family.
HMPREF1120_05299	paralog											-0.8	4.87E-07	1.8	6.57E-46	CelA	Putatively involved in 1,3-β-/1,4-β-glucan synthesis
HMPREF1120_02373	2	1	1	1	2	2	1	2	2	0	1	-1.55	1.03E-20	-0.65	8.03E-05	Mlg1	Mixed-linked glucanases in C. carbonum, hydrolyze 1,3-β-/1,4-β-glucans
HMPREF1120_07765	paralog											1.4	7.25E-19	-1.72	2.22E-40	Mlg1	
HMPREF1120_09051	1	2	1	1	0	0	0	1	1	0	0	3.95	8.92E-122	0.22	1.62E-01	Mlg1	
**Other cell wall biosynthesis proteins**																	
HMPREF1120_04431	1	1	1	1	1	1	1	1	1	0	0	1.11	4.02E-12	0.09	5.54E-01		Endo-mannanase family (GH 76) with a putative role in GPI-CWP incorporation; related to *S. cerevisiae* Dfg5
HMPREF1120_03513	1	1	1	1	0	0	1	1	1	0	0	0.19	3.28E-01	-0.08	6.40E-01		
HMPREF1120_05522	1	2	2	1	2	2	2	2	1	1	1	2.67	1.53E-58	-0.19	1.82E-01		
AN0393 (dfgC), AN3049 (dfgD), AN8421 (dfgB) AN0383 (dfgE)																	
HMPREF1120_03851	1	1	1	1	1	1	1	1	1	2	2	1.73	8.73E-29	-0.06	7.18E-01		Putative enzyme involved in cell wall biosynthesis with unknown function
HMPREF1120_08023	1	1	1	1	1	1	2	2	2	2	0	0.21	2.21E-01	-0.4	4.07E-03		Putative chitin deacetylases; similarity with ScCda1 and ScCda2

^ OrthoMCL clusters were identified based on the gene list in de Groot 2009, Fungal Genetics and Biology. For the GH18 family, we show only those involved in cell wall repair, which are listed by OrthoMCL cluster

***** *W. dermatitidis* gene IDs are listed for each OrthoMCL cluster. If a cluster does not have a *W. dermatitidis* gene, then *A. nidulans* gene IDs are listed.

****** log_2Fold Change for pH is of pH2.5 compared to pH6, and log_2Fold for radiation is of radiation treatment compared to untreated cells. Red indicates significant up-regulation under stress conditions (pH2.5 or with radiation) (corrected P-value < 1E-10), and blue indicates significant down-regulation under stress (corrected P-value < 1E-10).

******* P-values are corrected by the Benjamini and Hochberg method.

Gene symbols and descriptions are from de Groot 2009, Fungal Genetics and Biology.

& Split into separate rows, as they were named separately in de Groot 2009, although OrthoMCL placed Chs1 and Chs2 in the same ortholog cluster.
paralog indicates that the counts for the *W. dermatitidis* gene listed is absent as it is the paralog of the gene listed above it.
