# Supplementary material for: Comparative Genomic and Transcriptomic Analysis of Wangiella dermatitidis, A Major Cause of Phaeohyphomycosis and a Model Black Yeast Human Pathogen
Source: G3 (Bethesda). 2014 Feb 4;4(4):561–78. doi: 10.1534/g3.113.009241 (PMC4059230; doi:10.1534/g3.113.009241)
Supplement: Supporting Information [file supp_g3.113.009241_TableS3.pdf]

Table S3 Pathways involved in cell wall stress response and pH signaling.

Gene name	*A. nidulans*	*W. dermatitidis*	pH		Radiation			*A. niger* pH2.5 Vs pH6		
			Log_2Fold	P-value#	Log_2Fold	P-value#	Description	Gene ID	Log_2Fold	P-value
**Cell wall integrity pathway**										
Mid2	AN4897	HMPREF1120_03784	**1.48**	**2.04E-21**	0.55	3.01E-05	Transmembrane sensor for cell wall integrity signaling	An02g06660	-0.47	9.44E-02
Wsc1-3	AN6927	HMPREF1120_06587	**1.91**	**1.03E-33**	-0.57	1.84E-05	Sensor-transducer of the stress-activated PKC1-MPK1 kinase pathway	An01g09460<br>An02g13710	0.31<br>-0.25	3.51E-01<br>4.04E-01
	AN5660	HMPREF1120_05003	**1.5**	**3.32E-21**	0.48	3.00E-04		An03g00250<br>An07g04070	-0.10<br>0.31	6.54E-01<br>3.51E-01
	AN4674									
Rom2	AN4719	HMPREF1120_04966	-0.28	9.43E-02	-0.02	8.97E-01	GDP/GTP exchange factor (GEF)	An07g05090	0.41	8.59E-02
Rho1	AN5740	HMPREF1120_04193	0.48	2.63E-03	0.02	9.24E-01	Rho family GTPase	An18g05980	-0.32	1.35E-01
Rgd1	AN4745	HMPREF1120_02725	0.53	1.02E-03	0.19	1.98E-01	GTPase-activating protein	An11g10060	0.03	9.16E-01
Pkc1	AN0106	HMPREF1120_07353	0.34	3.97E-02	0.02	9.10E-01	Protein kinase C	An18g02400	-0.38	8.30E-02
Bkc1	AN4887	HMPREF1120_03472	0.37	2.49E-02	0.07	6.58E-01	mitogen-activated protein kinase kinase kinase (MAPKKK)	An02g06830	0.15	6.09E-01
Mkk1	AN4189	HMPREF1120_00691	**1.03**	**2.37E-10**	-0.1	4.94E-01	MAP kinase kinase (MAPKK)	An18g03740	0.24	2.43E-01
Mpk1	AN5666	HMPREF1120_06473	**2.32**	**4.15E-48**	0.09	5.59E-01	Serine/threonine MAP kinase	An01g09520	0.06	8.88E-01
Rlm1	AN2984	HMPREF1120_02025	**1.65**	**4.06E-25**	-0.35	1.19E-02	MADS-box transcription factor	An02g12210	-0.02	9.47E-01
Pst1	AN4390	HMPREF1120_03851	**1.73**	**8.73E-29**	-0.06	7.18E-01	GPI-anchored cell wall organization protein	An04g01230		
**UDP-GlcNAc synthesis pathway, required for chitin synthesis, GPI anchor biosynthesis and N-chain formation**										
gfaA	AN10709	HMPREF1120_00602	0.56	4.21E-04	-0.36	6.22E-03	Glutamine-fructose-6-phosphate transaminase (1st step)	An18g06820	2.16	2.63E-05
gnaA	AN8706	HMPREF1120_05484	0.5	2.43E-03	-0.19	2.20E-01	Glucosamine-phosphate N-acetyltransferase (2nd step)	An12g07840	0.39	3.14E-01
pcmA	AN4234	HMPREF1120_02062	0.3	7.16E-02	**-0.89**	**1.79E-11**	Phosphoacetylglucosamine mutase (3rd step)	An18g05160<br>An18g05170		
ungA	AN9094	HMPREF1120_05326	-0.03	8.59E-01	0.18	1.94E-01	UDP-N-acetylglucosamine pyrophosphorylase (4th step)	An12g00480	0.06	8.70E-01
**HOG signaling pathway**										
SLN1	AN1800	HMPREF1120_04358	0.78	8.33E-07	**0.85**	**3.84E-11**	Histidine kinase osmosensor that regulates a MAP kinase cascade			
YPD1	AN2005	HMPREF1120_08241	0.13	4.60E-01	0.4	3.41E-03	Phosphorelay intermediate protein	An04g06570	0.36	2.02E-01
SSK1	AN7697	HMPREF1120_04973	0.35	3.62E-02	0.37	5.95E-03	Cytoplasmic response regulator	An03g04670	0.04	8.48E-01
SSK2	AN10153	HMPREF1120_04310	0.43	8.79E-03	-0.33	1.70E-02	MAP kinase kinase kinase of the HOG1 signaling pathway	An08g03240	-0.05	8.99E-01
PBS2	AN0931	HMPREF1120_02538	0.52	1.13E-03	0.15	3.18E-01	MAP kinase kinase of the HOG signaling pathway	An01g11080	0.04	8.94E-01
HOG1	AN1017	HMPREF1120_05833	0.03	8.92E-01	0.08	6.11E-01	Mitogen-activated protein kinase involved in osmoregulation	An08g05850	-0.14	5.70E-01
**Ca2+/calcineurin signaling pathway**										
CCH1	AN1168	HMPREF1120_08350	**2.91**	**6.25E-72**	-0.7	5.25E-08	Voltage-gated high-affinity calcium channel	An08g03400	-0.23	2.92E-01
MID1	AN8842	HMPREF1120_05098	0.25	1.48E-01	0.21	1.44E-01	N-glycosylated integral membrane protein of the ER and plasma membrane	An17g02075	-0.12	5.46E-01
CMD1	AN2047	HMPREF1120_06249	-0.04	8.47E-01	0.36	7.07E-03	Calmodulin	An04g07010<br>An07g01640	0.44<br>0.03	1.71E-01<br>9.52E-01
CNA1	AN8820	HMPREF1120_03111	0.39	1.81E-02	-0.84	1.34E-10	Calcineurin A	An07g03620	-0.31	3.01E-01
CNB1	AN6566	HMPREF1120_02693	-0.06	7.60E-01	0.33	2.35E-02	Calcineurin B	An15g00870	-0.26	5.00E-01
CRZ1	AN5726	HMPREF1120_03777	0.16	3.81E-01	0.1	5.26E-01	Transcription factor that activates genes involved in stress response	An18g05920	0.31	3.18E-01
**pH signaling pathway**										
PacC	AN2855	HMPREF1120_00699	-0.36	2.89E-02	0.04	8.08E-01	Transcription factor	An02g07890	-3.03	8.18E-05
PalA	AN4351	HMPREF1120_02658	-0.24	1.72E-01	0.42	2.90E-03		An04g00760	-0.01	9.89E-01
PalB	AN0256	HMPREF1120_07519	0.14	4.67E-01	-0.01	9.38E-01	cysteine protease	An01g04680	-0.33	2.25E-01
PalC	AN7560	HMPREF1120_08758	-0.17	3.63E-01	-5.05	7.75E-01		An15g03350	-0.33	3.62E-01
PalF	AN1844	HMPREF1120_00084	0.97	1.14E-09	0.5	1.28E-04		An04g07460		
PalH	AN6886	HMPREF1120_01470	0.19	2.68E-01	0.25	7.19E-02	plasma membrane pH sensor	An14g04650	-0.08	7.90E-01
PalI	AN4853	HMPREF1120_06064	0.72	6.13E-06	-0.18	2.15E-01	plasma membrane pH sensor	An02g13150	0.05	8.59E-01
Vsp32/Snf7	AN4240	HMPREF1120_03523	-0.3	9.04E-02	-0.03	8.50E-01		An18g05430	-0.04	8.96E-01
**Chitin synthase genes**										
CHS2		HMPREF1120_06816	**1.75**	**7.17E-28**	0.49	2.88E-04		An07g05570	-0.14	6.42E-01
CHS1		HMPREF1120_07981	0.38	2.19E-02	**-1.73**	**1.68E-24**		An14g00660	0.05	9.16E-01
CHS3		HMPREF1120_06479	**1.86**	**3.01E-32**	0.43	1.35E-03		An09g04010	-1.03	4.18E-03
								An12g10380	1.98	1.85E-04
								An03g06360	0.11	5.76E-01
CHS4		HMPREF1120_07721	**1.32**	**2.77E-17**	-0.23	9.57E-02		An09g02290	-0.46	5.45E-02
CHS5		HMPREF1120_08776	**1.31**	**3.54E-17**	-0.68	1.18E-07		An02g02340	-0.28	4.16E-01
CHS7		HMPREF1120_08777	**1.48**	**1.82E-21**	-0.65	3.85E-07		An02g02360		
CHS6		HMPREF1120_09115	0.04	8.82E-01	-0.34	1.22E-02		An08g05290	0.47	7.67E-02

# P-values are corrected by the Benjamini and Hochberg method.
