# Supplementary material for: Comparative Genomic and Transcriptomic Analysis of Wangiella dermatitidis, A Major Cause of Phaeohyphomycosis and a Model Black Yeast Human Pathogen
Source: G3 (Bethesda). 2014 Feb 4;4(4):561–78. doi: 10.1534/g3.113.009241 (PMC4059230; doi:10.1534/g3.113.009241)
Supplement: Supporting Information [file supp_g3.113.009241_TableS4.pdf]

Table S4 GSEA tests on gene sets defined by InterPro domain, KEGG pathway, COG, MFS and APC transporter, and cell wall and related pathway.

Gene set ID	Gene set description	Size	pH enrichment ES*	pH enrichment NES*	pH enrichment q-value#	Radiation enrichment ES*	Radiation enrichment NES*	Radiation enrichment q-value#
**InterPro domain (q-value < 0.05)**								
IPR001128	Cytochrome P450	65	0.565	2.012	4.269E-03	-0.592	-2.121	2.050E-04
IPR003819	Taurine catabolism dioxygenase TauD/TfdA	16	0.741	1.967	5.661E-03	-0.431	-1.159	6.033E-01
IPR001461	Peptidase A1	10	0.829	1.934	6.252E-03	-0.287	-0.686	9.728E-01
IPR016196	Major facilitator superfamily, general substrate transporter	318	0.463	1.955	6.563E-03	-0.336	-1.487	2.293E-01
IPR021109	Peptidase aspartic	11	0.819	1.976	6.748E-03	-0.486	-1.157	6.049E-01
IPR008972	Cupredoxin	16	0.715	1.919	6.925E-03	-0.424	-1.126	6.430E-01
IPR006593	Cytochrome b561/ferric reductase transmembrane	6	0.936	1.888	1.006E-02	-0.285	-0.576	9.868E-01
IPR011701	Major facilitator superfamily MFS-1	230	0.455	1.858	1.346E-02	-0.295	-1.253	5.108E-01
IPR020846	Major facilitator superfamily	238	0.435	1.791	3.120E-02	-0.342	-1.464	2.599E-01
IPR002293	Amino acid/polyamine transporter I	52	0.521	1.768	3.712E-02	-0.405	-1.375	3.996E-01
IPR011707	Multicopper oxidase, type 3	13	0.700	1.753	3.763E-02	-0.485	-1.228	5.463E-01
IPR016048	Luciferase-like, subgroup	8	0.783	1.755	3.788E-02	0.300	0.682	1.000E+00
IPR004841	Amino acid permease domain	50	0.517	1.762	3.878E-02	-0.382	-1.295	4.721E-01
IPR001117	Multicopper oxidase, type 1	13	0.700	1.759	3.910E-02	-0.485	-1.189	5.816E-01
IPR011706	Multicopper oxidase, type 2	13	0.700	1.755	3.949E-02	-0.476	-1.180	5.897E-01
IPR005828	General substrate transporter	66	0.490	1.729	4.885E-02	-0.488	-1.743	3.136E-02
IPR020946	Flavin-containing monooxygenase-like	27	0.581	1.719	5.322E-02	-0.637	-1.876	5.444E-03
IPR012132	Glucose-methanol-choline oxidoreductase	9	0.700	1.602	1.568E-01	-0.757	-1.711	4.319E-02
IPR007219	Transcription factor, fungi	146	0.383	1.511	2.594E-01	-0.418	-1.691	4.914E-02
IPR001752	Kinesin, motor domain	11	0.537	1.297	5.918E-01	-0.798	-1.905	3.565E-03
IPR008775	Phytanoyl-CoA dioxygenase	11	0.505	1.219	6.921E-01	-0.732	-1.723	3.895E-02
IPR000873	AMP-dependent synthetase/ligase	45	0.352	1.168	7.428E-01	-0.618	-2.041	5.110E-04
IPR020617	Thiolase, C-terminal	8	0.520	1.166	7.449E-01	-0.908	-1.939	2.245E-03
IPR002155	Thiolase	8	0.520	1.146	7.650E-01	-0.908	-1.940	2.299E-03
IPR020616	Thiolase, N-terminal	7	0.531	1.117	7.759E-01	-0.900	-1.891	4.522E-03
IPR006092	Acyl-CoA dehydrogenase, N-terminal	16	0.335	0.877	9.679E-01	-0.803	-2.098	2.520E-04
IPR009100	Acyl-CoA dehydrogenase/oxidase	19	0.318	0.863	9.786E-01	-0.816	-2.264	0.000E+00
IPR009075	Acyl-CoA dehydrogenase/oxidase C-terminal	19	0.318	0.858	9.825E-01	-0.816	-2.229	0.000E+00
IPR001753	Crotonase, core	16	0.201	0.538	1.000E+00	-0.785	-2.072	2.920E-04
IPR003395	RecF/RecN/SMC	7	0.312	0.667	1.000E+00	-0.845	-1.771	2.137E-02
IPR006090	Acyl-CoA oxidase/dehydrogenase, type 1	16	0.250	0.668	1.000E+00	-0.819	-2.154	0.000E+00
IPR006091	Acyl-CoA oxidase/dehydrogenase, central domain	17	0.253	0.673	1.000E+00	-0.823	-2.162	0.000E+00
IPR006139	D-isomer specific 2-hydroxyacid dehydrogenase, catalytic domain	9	0.297	0.674	1.000E+00	-0.809	-1.858	7.151E-03
IPR012000	Thiamine pyrophosphate enzyme, central domain	9	0.246	0.565	1.000E+00	-0.745	-1.698	4.716E-02
IPR012001	Thiamine pyrophosphate enzyme, N-terminal TPP-binding domain	9	0.246	0.551	1.000E+00	-0.745	-1.704	4.475E-02
IPR015830	Amidase, fungi	10	0.291	0.707	1.000E+00	-0.841	-1.908	3.433E-03
IPR001509	NAD-dependent epimerase/dehydratase	23	-0.642	-2.143	3.114E-03	0.348	1.044	8.678E-01
IPR001163	Like-Sm ribonucleoprotein (LSM) domain	16	-0.676	-2.057	6.299E-03	0.273	0.745	9.837E-01
IPR006195	Aminoacyl-tRNA synthetase, class II	17	-0.656	-2.027	1.016E-02	0.471	1.317	6.736E-01
IPR015424	Pyridoxal phosphate-dependent transferase, major domain	53	-0.491	-1.990	1.185E-02	-0.475	-1.621	9.369E-02
IPR009000	Translation elongation/initiation factor/Ribosomal, beta-barrel	19	-0.624	-1.994	1.198E-02	0.305	0.867	9.149E-01
IPR023210	NADP-dependent oxidoreductase domain	28	-0.563	-2.000	1.230E-02	0.469	1.524	4.446E-01
IPR016027	Nucleic acid-binding, OB-fold-like	44	-0.501	-1.994	1.268E-02	-0.243	-0.811	9.475E-01
IPR009080	Aminoacyl-tRNA synthetase, class 1a, anticodon-binding	10	-0.757	-1.964	1.348E-02	0.304	0.738	9.842E-01
IPR001395	Aldo/keto reductase	28	-0.560	-1.970	1.400E-02	0.508	1.622	3.016E-01
IPR006649	Like-Sm ribonucleoprotein (LSM) domain, eukaryotic/archaea-type	16	-0.676	-1.964	1.409E-02	0.273	0.763	9.759E-01
IPR002423	Chaperonin Cpn60/TCP-1	10	-0.724	-1.941	1.675E-02	0.390	0.955	8.640E-01
IPR004161	Translation elongation factor EFTu/EF1A, domain 2	13	-0.679	-1.913	2.253E-02	0.288	0.755	9.795E-01
IPR002917	GTP-binding protein, HSR1-related	11	-0.720	-1.910	2.290E-02	0.639	1.633	2.897E-01
IPR020568	Ribosomal protein S5 domain 2-type fold	29	-0.532	-1.906	2.295E-02	-0.204	-0.619	9.835E-01
IPR014014	RNA helicase, DEAD-box type, Q motif	23	-0.569	-1.886	2.785E-02	0.670	2.040	4.650E-03
IPR001353	Proteasome, subunit alpha/beta	14	-0.635	-1.860	3.594E-02	-0.418	-1.076	7.106E-01
IPR019781	WD40 repeat, subgroup	103	-0.393	-1.842	4.190E-02	0.233	0.965	8.687E-01
IPR015815	3-hydroxyacid dehydrogenase/reductase	10	-0.596	-1.535	2.884E-01	-0.780	-1.847	8.142E-03
IPR016161	Aldehyde/histidinol dehydrogenase	21	-0.449	-1.440	3.935E-01	-0.741	-2.040	4.810E-04
IPR015590	Aldehyde dehydrogenase domain	21	-0.413	-1.349	4.911E-01	-0.750	-2.103	2.750E-04
IPR008927	6-phosphogluconate dehydrogenase, C-terminal-like	31	-0.344	-1.220	5.993E-01	-0.594	-1.840	8.589E-03
IPR012338	Beta-lactamase-type transpeptidase fold	7	-0.489	-1.149	6.303E-01	-0.820	-1.727	3.812E-02

IPR000425	Major intrinsic protein	9	-0.390	-0.991	7.736E-01	0.874	2.078	3.904E-03
IPR000120	Amidase	20	-0.301	-0.982	7.798E-01	-0.640	-1.792	1.609E-02
IPR004136	2-nitropropane dioxygenase, NPD	5	-0.433	-0.884	8.545E-01	-0.907	-1.704	4.553E-02
IPR006140	D-isomer specific 2-hydroxyacid dehydrogenase, NAD-binding	13	-0.255	-0.717	9.610E-01	-0.675	-1.709	4.349E-02
**KEGG pathway (q-value < 0.05)**								
ko05012	Parkinson's disease	53	-0.592	-2.457	6.850E-04	0.137	0.505	1.000E+00
ko05010	Alzheimer's disease	53	-0.552	-2.242	1.367E-03	0.121	0.449	9.986E-01
ko05016	Huntington's disease	69	-0.505	-2.155	4.106E-03	0.260	1.005	8.700E-01
ko00970	Aminoacyl-tRNA biosynthesis	13	-0.734	-2.105	4.071E-03	0.234	0.599	9.952E-01
ko00240	Pyrimidine metabolism	36	-0.566	-2.088	5.397E-03	0.255	0.863	9.170E-01
ko00591	Linoleic acid metabolism	35	-0.556	-2.068	6.218E-03	0.371	1.277	7.197E-01
ko04260	Cardiac muscle contraction	12	-0.718	-2.009	1.233E-02	0.333	0.847	9.328E-01
ko03020	RNA polymerase	20	-0.634	-2.001	1.303E-02	0.662	1.962	1.722E-02
ko00650	Butanoate metabolism	72	-0.436	-1.918	2.177E-02	-0.411	-1.509	1.993E-01
ko00363	Bisphenol degradation	43	-0.490	-1.889	2.778E-02	0.333	1.185	7.491E-01
ko03420	Nucleotide excision repair	23	-0.564	-1.852	3.903E-02	-0.425	-1.213	5.602E-01
ko03040	Spliceosome	16	-0.608	-1.849	3.907E-02	0.319	0.906	8.917E-01
ko00360	Phenylalanine metabolism	26	-0.350	-1.211	5.885E-01	-0.772	-2.286	0.000E+00
ko00380	Tryptophan metabolism	26	-0.264	-0.914	8.342E-01	-0.752	-2.264	0.000E+00
ko00280	Valine, leucine and isoleucine degradation	27	0.237	0.709	1.000E+00	-0.744	-2.222	0.000E+00
ko00627	Aminobenzoate degradation	45	0.371	1.214	6.982E-01	-0.647	-2.159	0.000E+00
ko00350	Tyrosine metabolism	34	-0.262	-0.963	7.876E-01	-0.675	-2.082	2.330E-04
ko00410	beta-Alanine metabolism	27	-0.257	-0.905	8.393E-01	-0.699	-2.054	4.760E-04
ko00960	Tropane, piperidine and pyridine alkaloid biosynthesis	17	0.369	0.994	8.877E-01	-0.741	-1.989	1.018E-03
ko00626	Naphthalene degradation	45	0.340	1.125	7.765E-01	-0.595	-1.971	1.441E-03
ko00643	Styrene degradation	10	-0.387	-1.007	7.595E-01	-0.818	-1.948	2.291E-03
ko00281	Geraniol degradation	12	0.454	1.126	7.803E-01	-0.805	-1.947	2.231E-03
ko00362	Benzoate degradation	25	0.206	0.609	1.000E+00	-0.660	-1.946	2.220E-03
ko00950	Isoquinoline alkaloid biosynthesis	15	0.284	0.735	1.000E+00	-0.738	-1.909	3.565E-03
ko00310	Lysine degradation	18	-0.502	-1.554	2.785E-01	-0.695	-1.887	4.812E-03
ko00903	Limonene and pinene degradation	42	0.274	0.899	9.647E-01	-0.576	-1.872	5.913E-03
ko03320	PPAR signaling pathway	14	0.387	0.977	8.833E-01	-0.712	-1.833	9.350E-03
ko04113	Meiosis - yeast	35	-0.164	-0.594	9.812E-01	-0.570	-1.807	1.346E-02
ko00071	Fatty acid metabolism	9	0.621	1.417	3.881E-01	-0.794	-1.804	1.373E-02
ko00940	Phenylpropanoid biosynthesis	10	0.617	1.453	3.429E-01	-0.756	-1.727	3.885E-02
ko00640	Propanoate metabolism	32	0.225	0.689	1.000E+00	-0.558	-1.720	3.969E-02
ko04146	Peroxisome	23	0.419	1.197	7.181E-01	-0.577	-1.694	4.915E-02
ko03030	DNA replication	9	-0.660	-1.648	1.610E-01	-0.732	-1.694	4.851E-02
**COG category (q-value < 0.05)**								
COGJ	Translation, ribosomal structure and biogenesis	319	-0.510	-2.731	0.000E+00	0.245	1.190	7.557E-01
COGE	Amino acid transport and metabolism	447	-0.245	-1.329	5.086E-01	-0.426	-1.927	2.675E-03
COGZ	Cytoskeleton	50	0.236	0.802	1.000E+00	-0.540	-1.853	7.153E-03
**MFS and APC transporter (see Figure S1)**								
MFS core	MFS core gene	100	0.376	1.402	4.082E-01	-0.406	-1.54	1.637E-01
MFS shared	MFS share gene	102	0.514	1.95	6.208E-03	-0.401	-1.538	1.646E-01
MFS uniq	MFS uniq gene	51	0.535	1.82	2.033E-02	-0.38	-1.302	4.716E-01
MFS_SP core	MFS_SP core gene	21	0.485	1.374	4.409E-01	-0.569	-1.581	1.307E-01
MFS_SP shared	MFS_SP share gene	24	0.492	1.453	3.346E-01	-0.628	-1.857	7.153E-03
MFS_SP uniq	MFS_SP uniq gene	12	0.577	1.407	3.974E-01	-0.426	-1.046	7.352E-01
MFS_DHA1 core	MFS_DHA1 core gene	22	-0.335	-1.121	6.480E-01	0.241	0.714	9.977E-01
MFS_DHA1 shared	MFS_DHA1 share gene	24	0.472	1.391	4.100E-01	0.317	0.982	8.761E-01
MFS_DHA1 uniq	MFS_DHA1 uniq gene	6	0.46	0.922	9.365E-01	-0.427	-0.853	9.244E-01
MFS_ACS core	MFS_ACS core gene	16	0.611	1.64	1.071E-01	-0.565	-1.507	2.017E-01
MFS_ACS shared	MFS_ACS share gene	38	0.657	2.09	7.790E-04	-0.483	-1.548	1.638E-01
MFS_ACS uniq	MFS_ACS uniq gene	21	0.639	1.796	2.564E-02	-0.439	-1.236	5.342E-01
APC core	APC core gene	21	0.434	1.214	6.645E-01	-0.351	-0.992	8.025E-01
APC shared	APC share gene	14	0.642	1.647	1.035E-01	-0.514	-1.314	4.684E-01
APC uniq	APC uniq gene	17	0.582	1.541	2.216E-01	-0.549	-1.491	2.150E-01
APC_ACT core	APC_ACT core gene	6	0.499	1.003	8.630E-01	-0.567	-1.11	6.563E-01
APC_ACT shared	APC_ACT share gene	8	0.593	1.307	5.395E-01	-0.329	-0.707	9.716E-01
APC_ACT uniq	APC_ACT uniq gene	7	0.798	1.664	8.943E-02	-0.563	-1.196	5.801E-01
APC_YAT core	APC_YAT core gene	12	0.397	0.972	8.851E-01	-0.358	-0.893	8.908E-01
APC_YAT shared	APC_YAT share gene	5	0.746	1.401	3.971E-01	-0.681	-1.317	4.653E-01
APC_YAT uniq	APC_YAT uniq gene	10	0.483	1.134	7.469E-01	-0.543	-1.23	5.431E-01
**Cell wall and pathway gene (see Tables 1, S2 and S3)**								
Chitin_syn	Chitin synthase	7	0.876	1.872	1.081E-02	-0.528	-1.115	6.539E-01
Chitin_syn_reg	Chitin synthesis regulation	8	0.674	1.454	3.348E-01	-0.301	-0.65	9.806E-01
Chitin_degrad	Chitin degradation	8	-0.465	-1.132	6.377E-01	-0.519	-1.136	6.286E-01

Glucan_beta_pro	Glucan 1,3-beta processing	27	0.541	1.595	1.548E-01	-0.388	-1.159	6.096E-01
Melanin_biosyn	Melanin biosynthesis	21	0.658	1.836	1.672E-02	-0.352	-0.997	7.962E-01
CWI	Cell wall integrity pathway	12	0.731	1.813	2.152E-02	-0.325	-0.801	9.490E-01
HOG	HOG pathway	6	0.592	1.202	6.876E-01	0.516	1.089	8.472E-01
Calcineurin	Calcineurin pathway genes	6	0.622	1.23	6.533E-01	-0.517	-1.036	7.422E-01
UDP-GlcNAc_syn	UDP-GlcNAc synthesis pathway	4	0.659	1.188	6.989E-01	-0.557	-1.004	7.897E-01
pH_sig	pH signaling pathway	8	0.416	0.902	9.446E-01	0.423	0.961	8.749E-01
Motor_prot	Motor protein	17	0.618	1.699	6.160E-02	-0.704	-1.882	5.022E-03
Calcium_trp	Calcium transporter	15	0.698	1.833	1.660E-02	0.457	1.259	7.203E-01
Light_sensing	Light sensing gene	11	0.365	0.871	9.617E-01	0.378	0.95	8.688E-01

***** Enrichment scores (ES) and normalized enrichment scores (NES) that are greater than 0 indicates the gene families are enriched under stress conditions (low pH or with radiation); negative ES and NES scores indicates enrichment under normal conditions (pH 6 or without radiation).

Significant enrichments (q-value < 0.05) are highlighted in blue (pH experiment) and red (radiation experiment).
